# Bioorthogonal Sonodynamic Plug‐and‐Play Targeting Chimeras (SDPTAC) for Precise Targeted Protein Degradation

**DOI:** 10.1002/advs.202520975

**Published:** 2025-12-22

**Authors:** Yuhan Bao, Yaojin Zhu, Xinhao Wei, Yuxin Fang, Jiayi Zhu, Fei Gao, Guoqiang Dong, Shipeng He, Chunquan Sheng

**Affiliations:** ^1^ Institute of Translational Medicine or School of Medicine Shanghai University Shanghai P. R. China; ^2^ Department The Center for Basic Research and Innovation of Medicine and Pharmacy (MOE) School of Pharmacy Second Military Medical University (Naval Medical University) Shanghai P. R. China

**Keywords:** bioorthogonal chemistry, SDPTAC, sonodynamic therapy, targeted protein degradation

## Abstract

Targeted protein degradation (TPD) offers powerful therapeutic opportunities but is limited by poor tissue penetration and E3 ligase dependence. Herein, we develop Sonodynamic Plug‐and‐Play Targeting Chimeras (SDPTAC), an ultrasound (US)‐activated, bioorthogonal strategy for in situ protein degradation. SDPTAC assembles via an inverse electron‐demand Diels–Alder (IEDDA) click reaction between a sonosensitizer and tetrazine‐tagged ligands, generating reactive oxygen species (ROS) upon US to degrade bound proteins. This modular platform enabled efficient degradation of nuclear (bromodomain‐containing protein 4, BRD4), cytosolic (nicotinamide phosphoribosyl transferase, NAMPT), and membrane (discoidin domain receptor 1, DDR1) targets, suppressed oncogenic signaling, and achieved nearly complete tumor growth inhibition in vivo with negligible toxicity. SDPTAC thus establishes a versatile, deep‐penetrating, and clinically translatable approach for noninvasive protein modulation.

## Graphical abstract

Bioorthogonal SDPTAC harnesses US‐responsive **Ce6‐TCO** and Tz ligands via IEDDA click chemistry to assemble degraders in situ, generating ROS that selectively eliminate nuclear, cytosolic, and membrane proteins, thereby suppressing deep‐seated tumors in vivo.

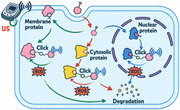

## 1 Introduction

Targeted protein degradation (TPD) has emerged as a cutting‐edge strategy in modern drug discovery [1, 2]. By leveraging the cell's endogenous proteolytic machinery, including the ubiquitin–proteasome system (UPS) [3, 4] and the autophagy–lysosome pathway [5, 6]. TPD selectively eliminates disease‐causing proteins to achieve therapeutic effects. Among the various TPD technologies, proteolysis‐targeting chimeras (PROTACs) have gained particular attention [7]. These heterobifunctional molecules simultaneously bind a protein of interest (POI) and an E3 ubiquitin ligase, promoting ubiquitination and subsequent proteasomal degradation of the POI. In contrast to traditional “occupancy‐driven” inhibitors, PROTACs function via an “event‐driven” catalytic mechanism, resulting in improved potency, reduced dosage requirements, and diminished resistance risks [8]. Currently, over 60 PROTACs have advanced into clinical trials, underscoring their promising therapeutic potential [2]. However, the PROTAC technology still faces several challenges [9, 10]. First, the large molecular weight of PROTACs often leads to poor cell membrane permeability and limited intracellular activity. Second, the non‐specific E3 ubiquitin ligase recruitment may induce off‐target effects, causing potential systemic toxicity and affecting normal tissue function. Furthermore, existing TPD methods primarily rely on chemically induced proximity, resulting in limited spatiotemporal control over the protein degradation process.

Photodegradation‐targeting chimeras (PDTACs) represent a promising complement to existing TPD strategies [11]. leveraging light‐activated photosensitizers to generate reactive oxygen species (ROS), particularly singlet oxygen (^1^O_2_). This process induces selective oxidation of critical amino acid residues and disrupts the structural integrity of POIs, leading to protein inactivation and degradation [12]. Liu et al., [11] provided the proof of concept by conjugating photosensitizer verteporfin to a glutathione peroxidase 4 (GPX4)‐targeting peptide. Upon near‐infrared (NIR) light irradiation, selective degradation of GPX4 was successfully achieved. Unlike conventional PROTACs, PDTACs achieve protein degradation through a physically triggered and E3‐ligase‐independent mechanism, offering improved spatiotemporal control and tissue selectivity, thereby minimizing damage to normal tissues [13, 14]. However, the limited tissue penetration depth of visible or NIR light (typically < 1 cm) severely constrains PDTACs’ utility in deep‐seated lesions, thus significantly impeding their clinical translation [15].

To overcome this fundamental limitation, sonodynamic therapy (SDT) has emerged as a promising alternative. By using ultrasound (US) to activate sonosensitizers, SDT generates ROS in deep tissues, achieving penetration depths exceeding 10 cm [16, 17]. Compared with photodynamic therapy (PDT), SDT offers superior tissue penetration, precise spatial control, and better clinical feasibility [18, 19]. Nevertheless, SDT also faces intrinsic limitations that restrict its application in TPD. Conventional sonosensitizers, such as porphyrins and their derivatives, typically exhibit large molecular size and limited tumor selectivity, resulting in inefficient cellular uptake and nonspecific subcellular localization [20]. These drawbacks reduce degradation precision and increase the risk of off‐target oxidative damage. To address these issues, bioorthogonal chemistry provides an attractive solution. By dividing functional components into smaller, cell‐permeable fragments that selectively react via bioorthogonal click chemistry inside cells, this approach minimizes molecular bulk, enhances intracellular accessibility, and enables spatiotemporally controlled in situ assembly of active degraders at the target site [21]. While bioorthogonal chemistry has been integrated with photoactivation and chemotherapy [22]. its combination with US‐mediated sonodynamic processes remains unexplored in the TPD field [23]. Such integration holds great potential to overcome the penetration and selectivity limitations of current degradation strategies.

**FIGURE 1:**
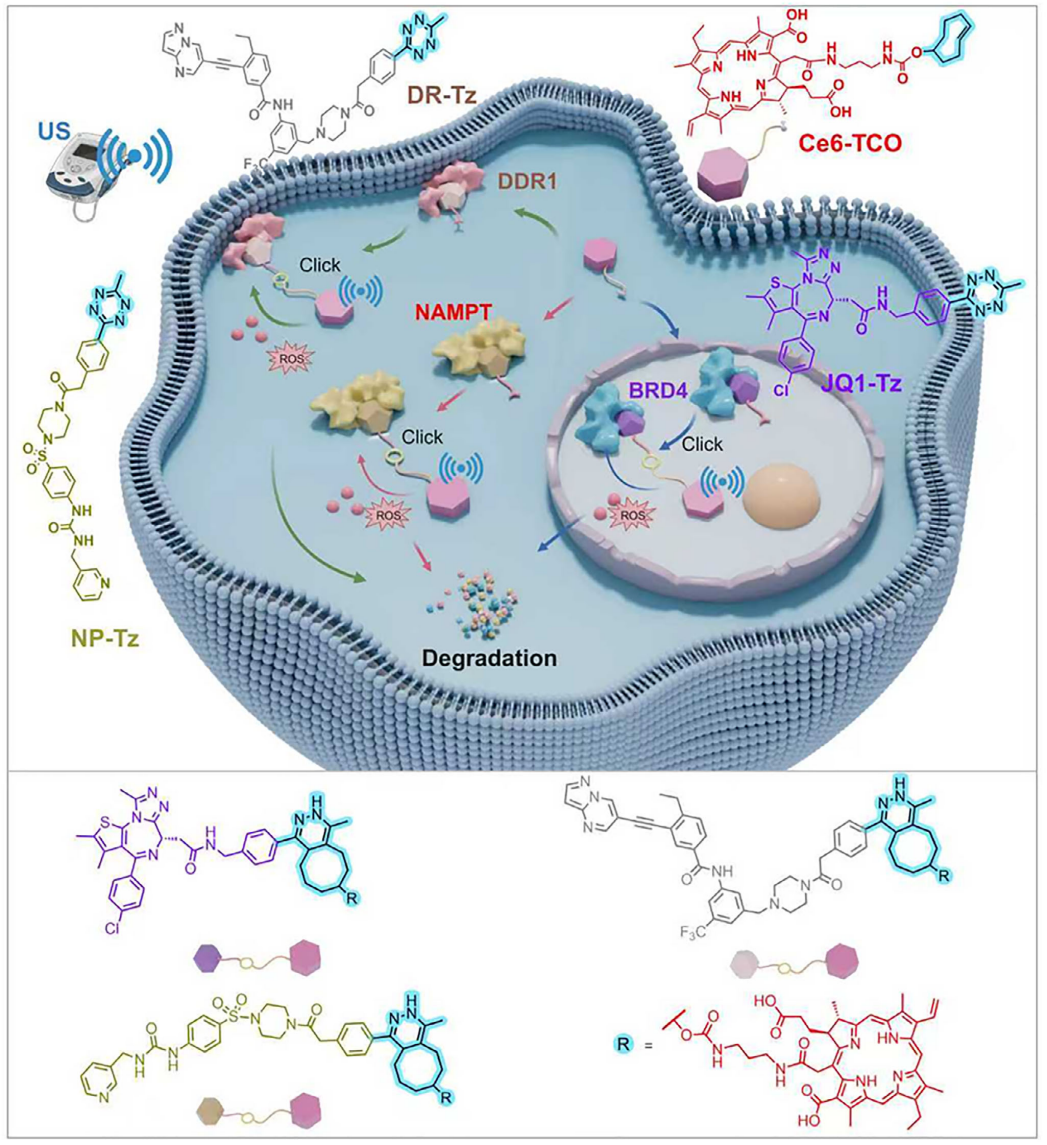
The principle of SPOTAC strategy. A TCO group is introduced at the side chain of sonosensitizer **Ce6**, while a **Tz** moiety is incorporated at the solvent‐exposed region of the POI ligand. After self‐assembly via the IEDDA reaction, the SDPTAC is formed within cells. Upon US activation, SDPTAC further triggers the generation of ROS, thereby enabling precise protein degradation.

Previously, we have developed a platform for precise modulation of protein degradation [24, 25, 26, 27]. To further improve the specificity and penetration of TPD, herein we developed a novel bioorthogonal, sonodynamic‐based strategy termed “Sonodynamic Plug‐and‐Play Targeting Chimeras” (SDPTAC). This strategy integrates US‐triggered sonodynamic degradation with bioorthogonal click chemistry to enable in situ assembly and activation of degradation‐inducing molecules at the target protein site (Figure 1). As a proof of concept, we demonstrated SDPTAC‐mediated degradation of nuclear oncogenic target bromodomain‐containing protein 4 (BRD4) both in vitro and in vivo. This approach was successfully extended to cytoplasmic protein nicotinamide phosphoribosyl transferase (NAMPT) and membrane protein discoidin domain receptor 1 (DDR1), achieving robust degradation with remarkable subcellular specificity and spatiotemporal control (Figure 1). Its modular and streamlined design ensures excellent cell membrane permeability and broad target adaptability, establishing SDPTAC as a versatile platform for the targeted degradation of disease‐associated proteins.

## 2 Results and Discussion

### 2.1 Design and Synthesis of SDPTAC Components

SDPTAC employs a bifunctional molecular system based on the inverse electron‐demand Diels–Alder (IEDDA) click reaction with high velocity and selectivity [28]. The system consists of a *trans*‐cyclooctene (TCO) conjugated sonosensitizer (chlorin e6, **Ce6**), and a target protein ligand linked to the tetrazine (Tz) moiety. Upon binding of the ligand‐Tz conjugate to the target protein, the **Ce6‐TCO** component is recruited, triggering a rapid IEDDA reaction to assemble the functional SDPTAC molecule in situ (Figure 1). Under US stimulation, SDPTAC generates ROS, particularly ^1^O_2_, inducing oxidative damage and subsequent degradation of the target protein. Therefore, SDPTAC is able to assemble an US‐responsive degrader directly on the protein target without perturbing the cellular environment.

**SCHEME 1:**
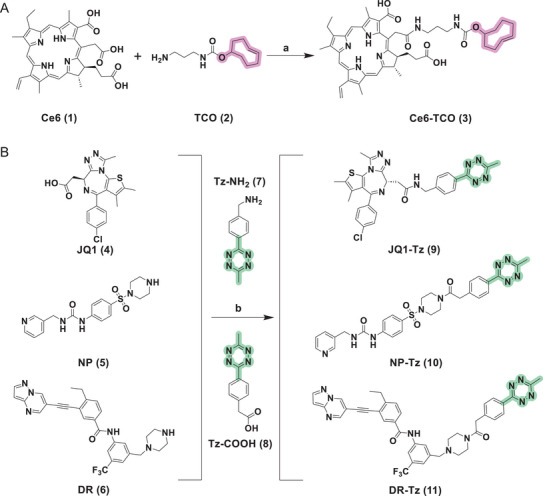
Synthesis of SDPTAC components (a) 1‐Ethyl‐3‐(3‐dimethylaminopropyl)carbodiimide hydrochloride (EDCI), 1‐Hydroxybenzotriazole (HOBt), N,N‐Diisopropylethylamine (DIPEA), r.t.; (b) 2‐(7‐Aza‐1H‐Benzotriazole‐1‐yl)‐1,1,3,3‐Tetramethyluronium Hexafluorophosphate (HATU), DIPEA, r.t.; Design and PD‐L1 degradation activity of HerTACs based on HER2‐targeted linear peptides.

Specifically, **Ce6** (**1**) was selected as the sonodynamic core and subsequently installed with a TCO **(2)** handle to furnish an acoustically activatable module, **Ce6‐TCO** (**3**). To endow the system with target specificity, we grafted Tz moieties onto three clinically valuable protein ligands, ensuring that each tag occupies a solvent‐exposed position. Once the ligand is bound to the target, it can rapidly couple with **Ce6‐TCO** for in situ SDPTAC assembly and activation (Scheme 1). As a conceptual validation study, BRD4 was used as a template protein for the SDPTAC design. **JQ1** (**4**) is a well‐characterized inhibitor of epigenetic regulator BRD4 [29], whose *tert*‐butyl group projects toward the solvent when complexed with BRD4 [30]. Thus, this site was derivatized with Tz to give **JQ1‐Tz** (**9**). The resulting conjugate binds BRD4 and then undergoes IEDDA ligation with **Ce6‐TCO** (Scheme 1), enabling US‐triggered and ROS‐mediated degradation of the nuclear protein. NAMPT is a key metabolic enzyme implicated in tumor progression [31]. The Tz handle was introduced at the solvent‐exposed terminus of NAMPT inhibitor **NP** (**5**) (Scheme S1), affording **NP‐Tz** (**10**) and thus allowing selective recognition and degradation of cytosolic NAMPT. DDR1 is a clinically relevant membrane tyrosine kinase [32]. DDR1 inhibitor **DR** (**6**, Scheme S2) was conjugated with Tz to generate **DR‐Tz** (**11**), which subsequently recruits **Ce6‐TCO** to the cell surface and directs US induced degradation of membrane protein DDR1. Together, the **Ce6‐TCO** module and the three Tz‐tagged ligands constitute a modular “plug and play” toolkit that can be rapidly reconfigured to degrade nuclear, cytoplasmic, or membrane proteins through bioorthogonal conjugation and sonodynamic activation.

### 2.2 Stability of SDPTAC Precursors and Optimization of US Parameters

**FIGURE 2:**
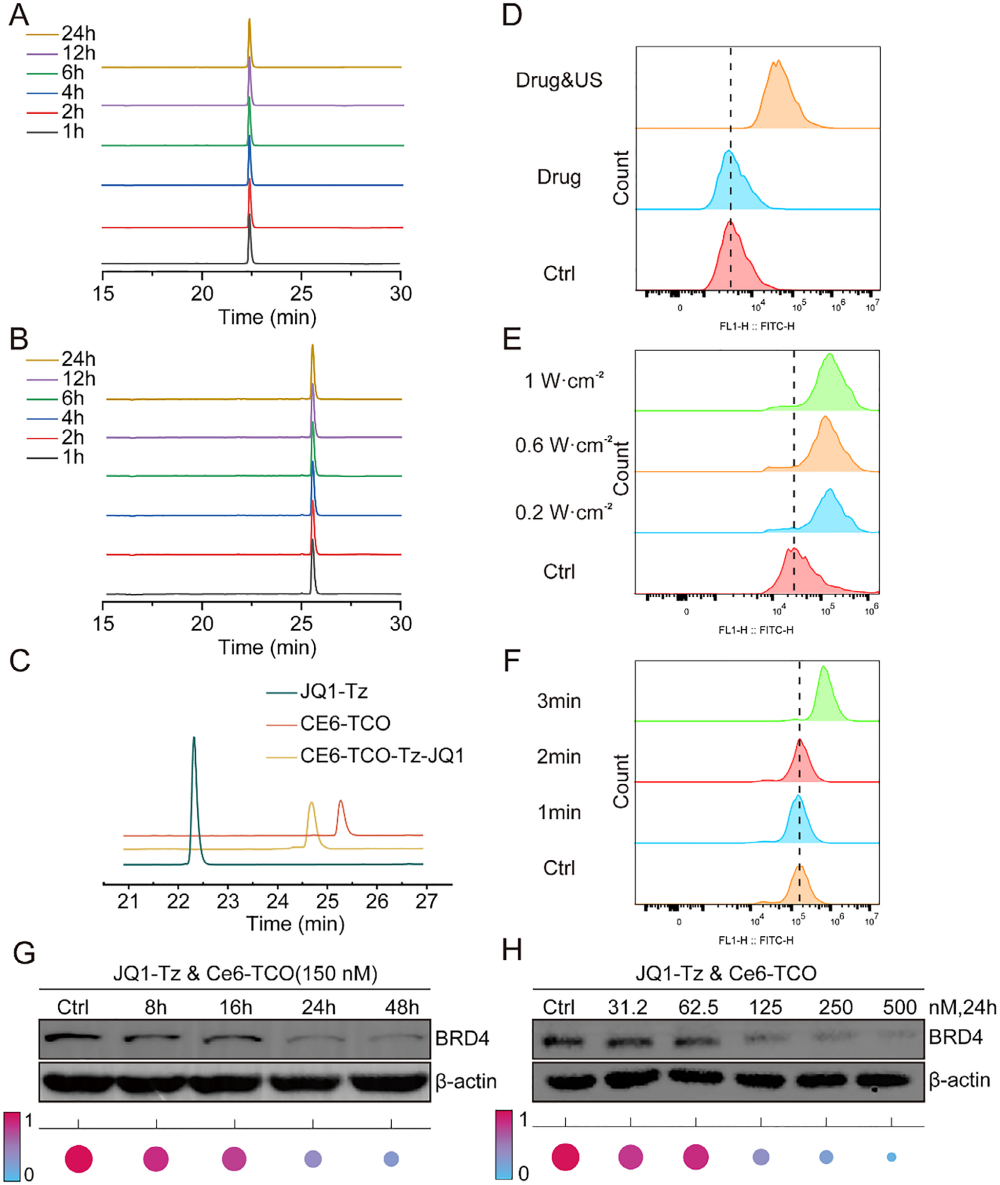
The structural stability of SDPTAC and the US conditions for the degradation activity. (A) Stability of **Ce6‐TCO** in DMEM, n = 3. (B) Stability of **JQ1‐Tz** in DMEM, n = 3. (C) Bioorthogonal efficiency between **Ce6‐TCO** and **JQ1‐Tz** within 30 min in DMEM, n = 3. (D) ROS generation efficiency in MDA‐MB‐231 cells stimulated by **Ce6‐TCO**+**JQ1‐Tz** with US (drug & US) and **Ce6‐TCO**+**JQ1‐Tz** without US (drug), respectively. (E) Effect of different US power levels on the ROS generation efficiency in MDA‐MB‐231 cells, n = 3. (F) Effect of different US durations on the ROS generation efficiency in MDA‐MB‐231 cells, n = 3. (G) BRD4 protein degradation activity at different time points after simultaneous addition of **JQ1‐Tz** (150 nM) and **Ce6‐TCO** (150 nM) in MDA‐MB‐231 cells, n = 3. (H) BRD4 protein degradation activity at 24 h after treatment with varying concentrations of **JQ1‐Tz** and **Ce6‐TCO** in MDA‐MB‐231 cells, n = 3.

The first step was to verify whether the two reactive components of SDPTAC (**JQ1‐Tz** and **Ce6‐TCO**) remain chemically intact under the cell‐culture conditions. High‐performance liquid chromatography (HPLC) analysis showed no detectable decomposition for either compound after 24 h incubation at 1 mM in Dulbecco's Modified Eagle Medium (DMEM) (Figure 2A,B), confirming excellent intrinsic stability. We next examined whether the IEDDA click reaction between **JQ1‐Tz** and **Ce6‐TCO** proceeds efficiently in a biological milieu. Equimolar mixing of the two species in DMEM for 30 min generated a single new HPLC peak corresponding to the conjugate, demonstrating a fast and high‐yield bioorthogonal ligation (Figure 2C).

To determine whether the assembled chimera could produce ROS under US stimulation, cells were incubated sequentially with **JQ1‐Tz** and **Ce6‐TCO**, stained with a ROS‐sensitive probe, and analyzed by flow cytometry. Baseline samples without US displayed negligible fluorescence, whereas insonation induced a robust intracellular ROS burst (Figures 2D; S1). Comparing two acoustic power densities revealed that both 0.6 W cm^−^^2^ and 1.0 W cm^−^^2^ (1 MHz, 50 % duty cycle) elicited strong ROS signals, while the higher intensity caused marked cytotoxicity (Figures 2E; S1). Consequently, 0.6 W cm^−^^2^ was selected as the optimal power. A time‐course study revealed that a 3 min exposure produced the maximal ROS output with minimal cellular stress (Figures 2F; S1), and these conditions were adopted for all subsequent experiments.

Under the optimized protocol (0.6 W cm^−^^2^, 3 min), we assessed target degradation in MDA‐MB‐231 cells. Cells were pre‐treated with **JQ1‐Tz** for 24 h, exposed to **Ce6‐TCO**, irradiated, and harvested at various time points. Western blotting showed a progressive decline in BRD4 abundance, culminating in near‐complete depletion after 24 h (Figures 2G; S2). Furthermore, we established the dose–response relationship for the two components. Cells received equimolar **JQ1‐Tz**/**Ce6‐TCO** (range: 31.2–500 nM), followed by US and a 24 h incubation. BRD4 levels were decreased in a concentration‐dependent manner, with clear degradation observable at 125 nM for each reagent (Figures 2H; S3). These data confirm that SDPTAC combines the advantages of high chemical stability, rapid intracellular assembly, efficient US‐triggered ROS generation, and potent catalytic protein degradation under physiologically compatible acoustic parameters.

### 2.3 Cellular Degradation Activity and Sequence Dependence

**FIGURE 3:**
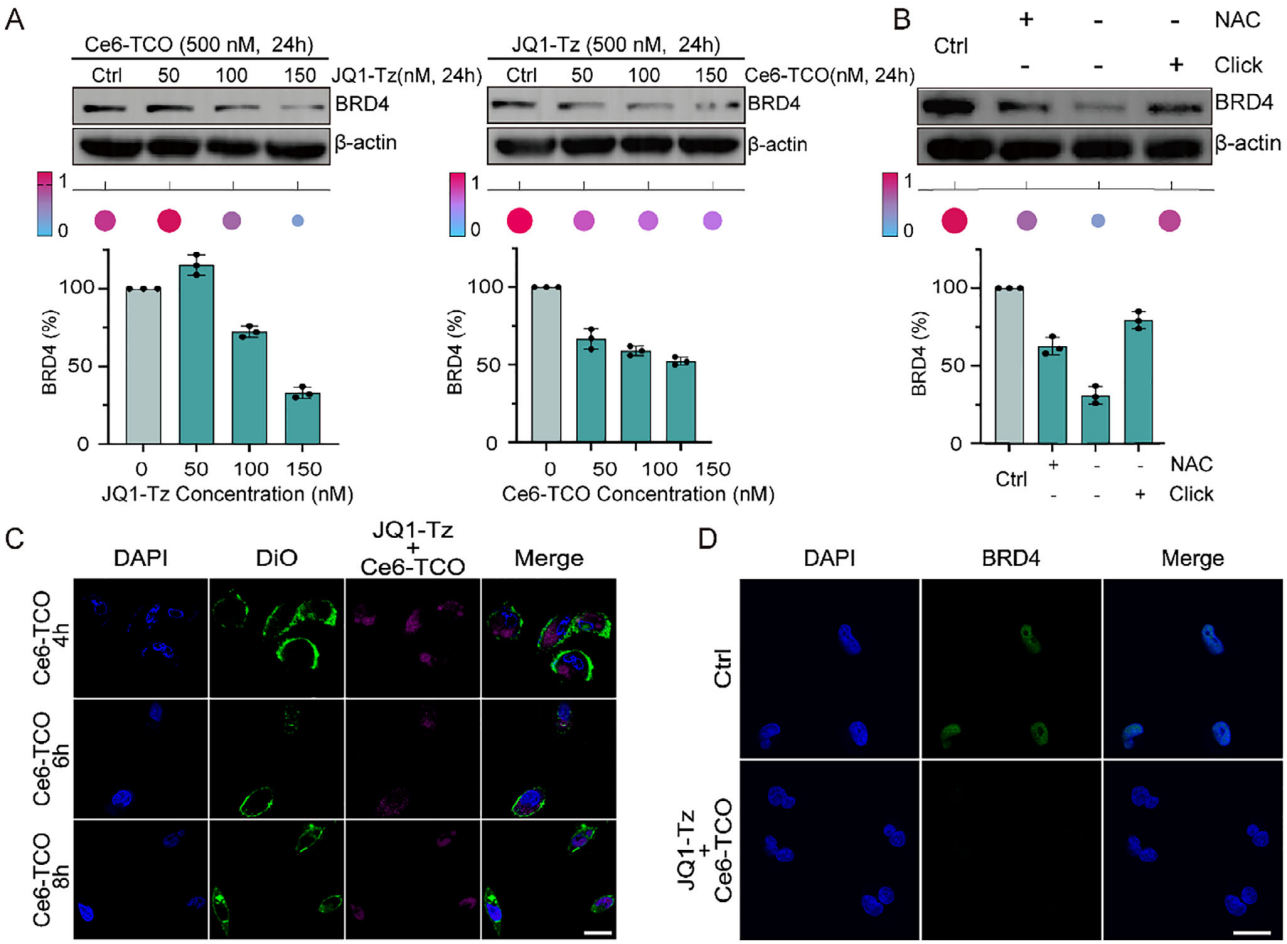
SDPTAC‐mediated BRD4 degradation activity and sequence dependence in MDA‐MB‐231 Cells. (A) Changes in degradation activity when **JQ1‐Tz** and **Ce6‐TCO** were pre‐incubated separately, n = 3, ^*^ *p* < 0.05, ^**^ *p* < 0.01. (B) Investigation of the mechanism by which SDPTAC exerts its effect, n = 3. Investigation of the mechanism by which SDPTAC exerts its effect, n = 3. Lane 1: untreated control; Lane 2: intracellular bioorthogonal assembly with NAC (ROS scavenger); Lane 3: intracellular bioorthogonal assembly without NAC; Lane 4: extracellular bioorthogonal assembly without NAC. (C) Confocal fluorescence images show that after pre‐treatment with **JQ1‐Tz**, **Ce6‐TCO** gradually localizes to the nucleus over time. Scale bar = 20 µm. (D) Immunofluorescence images showing BRD4 protein degradation in cells treated with SDPTAC (500 nM **JQ1‐Tz** + 50 nM **Ce6‐TCO** + US) for 48 h. Scale bar = 20 µm.

To dissect the catalytic efficiency and mechanistic requirements of SDPTAC, we first performed a series of Western blot assays under two dosing paradigms (Figures 3A; S4). When cells were pre‐incubated with **Ce6‐TCO** (500 nM, 24 h) and subsequently exposed to escalating concentrations of **JQ1‐Tz**, a clear reduction of BRD4 was detected at 100 nM **JQ1‐Tz**. Conversely, pre‐binding of BRD4 with **JQ1‐Tz** (500 nM, 24 h) followed by graded **Ce6‐TCO** resulted in protein loss only at 50 nM **Ce6‐TCO**. This two‐fold difference demonstrates that engagement of the ligand to BRD4 before recruiting the sonosensitiser markedly lowers the **Ce6‐TCO** threshold, underscoring the importance of dosing sequence for maximal degradation potency.

Control experiments confirmed the intracellular nature of the process (Figures 3B; S4). Either pre‐reacting 150 nM **JQ1‐Tz** with 150 nM **Ce6‐TCO** extracellularly (thus preventing in‐cell assembly) or co‐treating cells with the ROS scavenger *N*‐acetyl‐*L*‐cysteine (NAC) abolished BRD4 depletion, indicating that (i) IEDDA conjugation must occur inside the cell and (ii) ROS generation is indispensable for proteolysis. The cell viability assay results (Figure S5) demonstrated that SDPTAC induced a dose‐dependent reduction in cell viability in the MDA‐MB‐231 cancer cell line. In contrast, in the absence of US activation, SDPTAC exerts minimal effects on the viability of normal MCF‐10A cells across the tested concentrations. These findings indicate that SDPTAC selectively exerts cytotoxic effects in cancer cells while sparing normal cells, highlighting its potential for selective cancer therapy. Spatiotemporal tracking by confocal microscopy further delineated the trafficking route (Figures 3C; S6). Cells were labelled with DIO (3, 3′‐dioctadecyloxacarbocyanine perchlorate, membrane, green) and DAPI (4′,6‐Diamidino‐2‐phenylindole dihydrochloride, nucleus, blue), pre‐treated with **JQ1‐Tz** for 24 h, and then pulsed with **Ce6‐TCO**. Fluorescence progressively concentrated in the nucleus over 8 h, demonstrating that **Ce6‐TCO** follows its tethered counterpart into the nuclear compartment after intracellular click assembly.

Finally, immunofluorescence imaging corroborated functional degradation (Figure 3D). Cells receiving 500 nM **JQ1‐Tz**, 50 nM **Ce6‐TCO**, US (0.6 W cm^−2^, 3 min), and a 48 h chase displayed near‐complete disappearance of BRD4 signal, in sharp contrast to untreated controls. These data collectively establish that SDPTAC mediates efficient, ROS‐dependent, and sequence‐sensitive BRD4 degradation through intracellular IEDDA assembly and US activation.

### 2.4 Proteomic Level Analysis of SDPTAC

**FIGURE 4:**
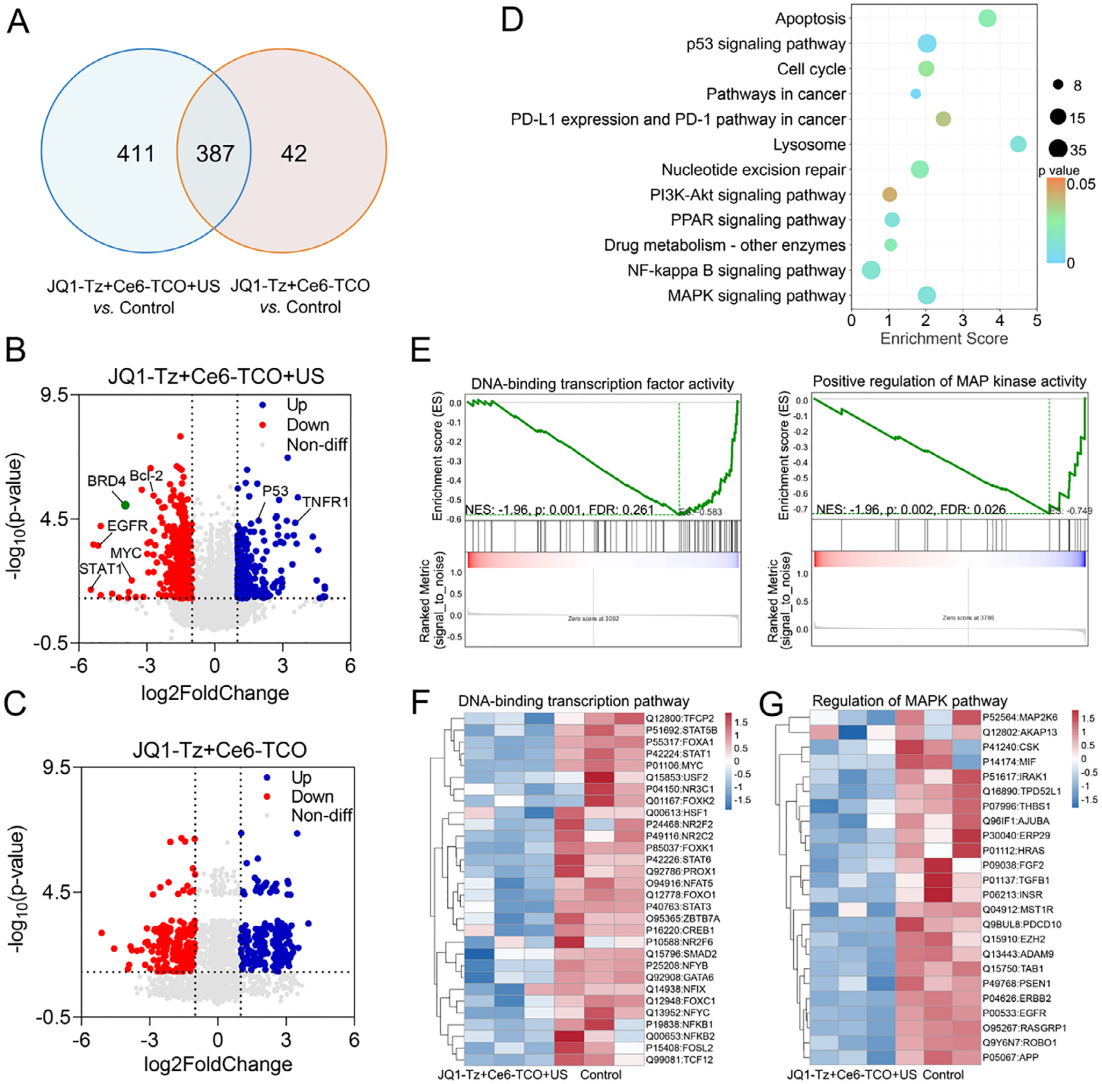
Proteome‐wide analysis of SDPTAC treatment in MDA‐MB‐231 cells. (A) Venn diagram showing the overlap of differentially expressed proteins between **JQ1‐Tz** + **Ce6‐TCO**+US‐treated and **JQ1‐Tz** + **Ce6‐TCO**‐treated control groups. (B,C) Volcano plots illustrating differentially expressed proteins in (B) **JQ1‐Tz** + **Ce6‐TCO** + US‐treated cells and (C) **JQ1‐Tz** + **Ce6‐TCO**‐treated controls (n = 3; fold change > 1.5; *p* < 0.05). Red, blue, and gray dots represent significantly downregulated, upregulated, and unchanged proteins, respectively. (D) KEGG pathway enrichment analysis showing altered signaling pathways upon **JQ1‐Tz** + **Ce6‐TCO** + US treatment. (E) GSEA indicates suppression of DNA‐binding transcription factor and MAPK regulatory pathways following **JQ1‐Tz** + **Ce6‐TCO** + US treatment. (F,G) Heatmaps of representative differentially expressed proteins in the (F) DNA replication pathway and (G) MAPK pathway between **JQ1‐Tz** + **Ce6‐TCO** + US‐treated and PBS‐treated control cells (n = 3; *p* < 0.05).

To further elucidate the protein degradation mechanism of the SDPTAC strategy, we performed a global proteomic analysis in MDA‐MB‐231 cells. **JQ1‐Tz** + **Ce6‐TCO** + US treatment perturbed the expression of 798 proteins, substantially more than the 429 proteins affected by **JQ1‐Tz** + **Ce6‐TCO** alone, with 387 proteins overlapping between the two groups (Figure 4A). Pathway‐level analysis revealed that SPOTAC profoundly reshaped cellular signaling networks. Volcano plot analysis indicated that **JQ1‐Tz** + **Ce6‐TCO**+US treatment resulted in 496 downregulated and 302 upregulated proteins, among which BRD4 was significantly downregulated (Figure 4B). In contrast, no differential expression of BRD4 was observed in the **JQ1‐Tz** + **Ce6‐TCO** group (Figure 4C), demonstrating the specific BRD4‐degrading activity of SPOTAC. KEGG enrichment analysis showed that the affected proteins were primarily involved in apoptosis, p53 signaling, cell cycle regulation, and mitogen‐activated protein kinase (MAPK) pathways (Figure 4D). Gene set enrichment analysis (GSEA) further revealed that **JQ1‐Tz** + **Ce6‐TCO** + US treatment suppressed critical biological pathways, including DNA‐binding transcription and MAPK regulation (Figure 4E). Notably, MYC and STAT proteins were downregulated within the DNA‐binding transcription pathway (Figure 4F), while macrophage migration inhibitory factor (MIF) and epidermal growth factor receptor (EGFR) were markedly decreased in the MAPK signaling pathway (Figure 4G). Collectively, these findings indicate that SDPTAC not only enables highly selective degradation of BRD4 but also triggers coordinated remodeling of multiple oncogenic signaling pathways, thereby exerting potent inhibitory effects on tumor cell activity. Importantly, although proteome‐wide profiling identified a substantial number of differentially expressed proteins, these alterations do not reflect off‐target degradation. BRD4 reduction is observed exclusively under the complete SDPTAC condition, confirming the specificity of the targeted degradation event. Consistent with this, pathway‐enrichment analyses show that the majority of dysregulated proteins fall within BRD4‐governed transcriptional networks, supporting that their changes arise from secondary regulatory cascades rather than direct proteolysis. In parallel, ROS generated upon sonodynamic activation mainly contributes to stress‐ and apoptosis‐related responses without inducing non‐specific protein degradation. Therefore, the extensive proteomic perturbation represents a systems‐level consequence of precise BRD4 degradation, rather than unintended off‐target effects. Given its defined BRD4 degradation capacity and potent antiproliferative effects, we further evaluated the in vivo antitumor mechanism and efficacy of the SDPTAC strategy.

### 2.5 In Vivo Antitumor Efficacy of the SDPTAC System in a Breast Cancer Model

**FIGURE 5:**
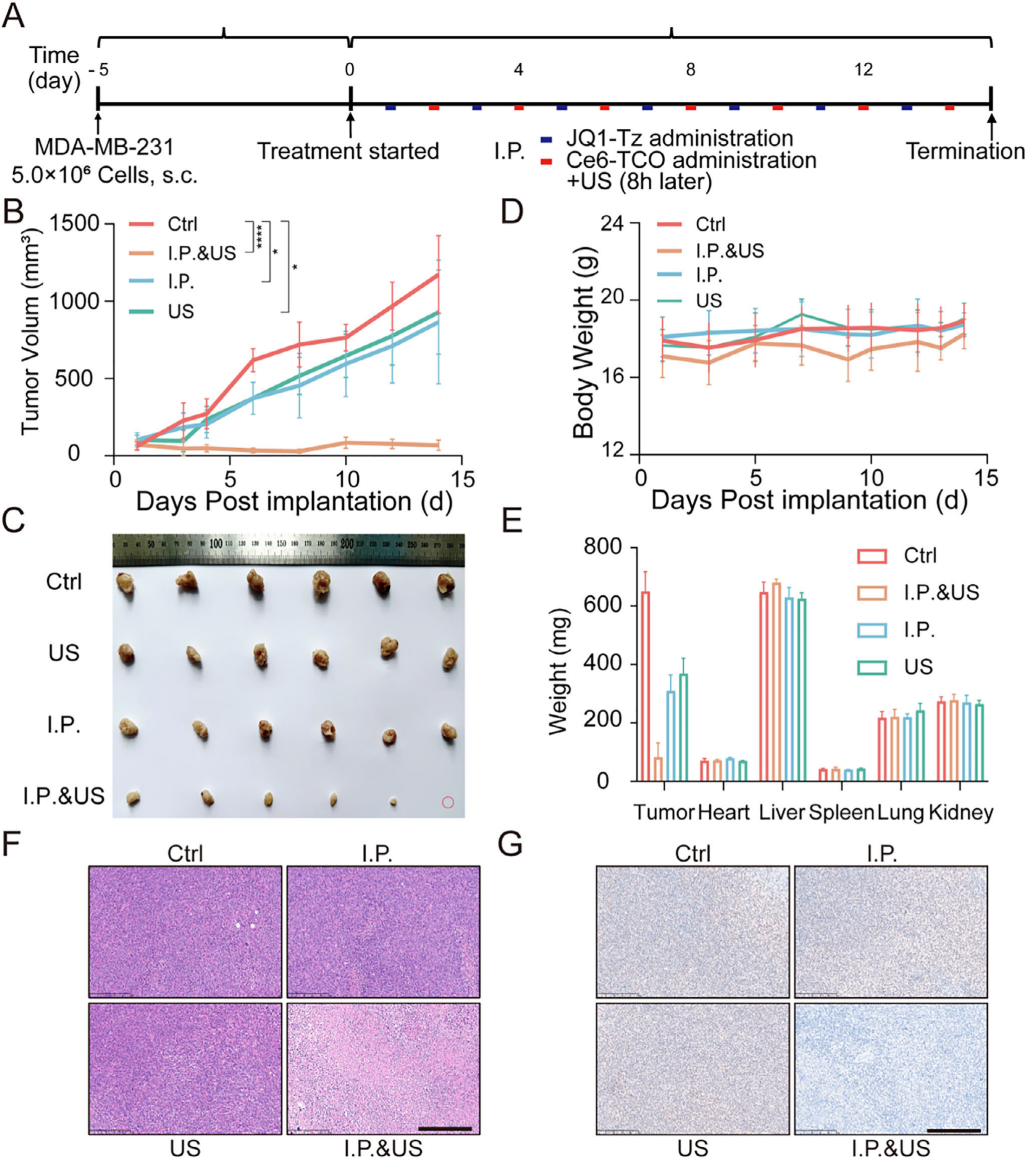
In vivo antitumor activity of SDPTAC. (A) Schematic diagram of the in vivo experimental protocol. Mice were treated via intraperitoneal injection (**JQ1‐Tz** at 10 mg kg^−1^ first, followed by **Ce6‐TCO** at 10 mg kg^−1^ after 24 h; US (0.6 W cm^−2^, 3 min) treatment was administered 8 h after **Ce6‐TCO** injection). The treatment was administered every other day for 14 days, n = 6. (B) Tumor volume changes over time in each treatment group, n = 6, ^*^ *p*< 0.05, ^****^ *p*< 0.0001. “i.p.” indicates intraperitoneal injection of **JQ1‐TCO** first, followed by injection of **Ce6‐Tz**. (C) Final tumor volumes of each treatment group at the end of the experiment, n = 6. (D) Body weight changes over time in each treatment group, n = 6. (E) Organ weights at the end of treatment, n = 6. (F) BRD4 protein staining in tumor tissues from each treatment group. Scale bar = 250 µm. (G) Ki‐67 staining in tumor tissues from each treatment group. Scale bar = 250 µm.

To determine whether SDPTAC retains activity in living organisms, we assessed its therapeutic performance in a human breast cancer xenograft model. Female BALB/c nude mice were inoculated subcutaneously with MDA‐MB‐231 cells, and treatment was initiated when tumors reached ∼100 mm^3^ (experimental timeline, Figure 5A). Animals first received an intraperitoneal (i.p.) injection of **JQ1‐Tz** (10 mg kg^−1^). After 24 h, they were given **Ce6‐TCO** (10 mg kg^−1^, i.p.), and a local US pulse (0.6 W cm^−^^2^, 3 min, 1 MHz) was applied 8 h after the **Ce6‐TCO** injection. This three‐step cycle was repeated every other day for a total of 14 days. Three control arms were designed: (i) vehicle only, (ii) **JQ1‐Tz** + **Ce6‐TCO** without US, and (iii) US alone. Tumor growth curves (Figure 5B,C) showed that the full “SDPTAC + US” regimen produced a pronounced and sustained inhibition of tumor progression, yielding a tumor‐growth inhibition (TGI) index of 94.2 %. In contrast, neither the dual‐drug arm lacking US nor the US‐only arm displayed statistically significant tumor suppression, underscoring the necessity of both bioorthogonal assembly and acoustic activation for therapeutic efficacy.

Throughout the study, body‐mass trajectories and post‐mortem organ weights of treated mice remained indistinguishable from the blank controls (Figure 5D,E), indicating minimal systemic toxicity. Immunohistochemical analysis corroborated the antitumor effect. Ki‐67 staining revealed a marked reduction in proliferating cells in the “SDPTAC + US” tumor group, whereas the other groups showed no significant change (Figure 5F). Notably, BRD4 immunoreactivity was profoundly diminished only in tumors exposed to the complete SDPTAC protocol (Figure 5G), confirming that in situ bioorthogonal assembly and US‐triggered ROS generation trigger target‐specific protein degradation in vivo.

Collectively, these findings demonstrate that SDPTAC achieves potent, BRD4‐dependent antitumor activity in a deep‐seated breast‐cancer model while maintaining a favorable safety profile, highlighting its promise as an US‐activated protein‐degradation therapy for solid tumors.

### 2.6 Broad Applicability of SDPTACs to Cytosolic and Membrane Proteins

**FIGURE 6:**
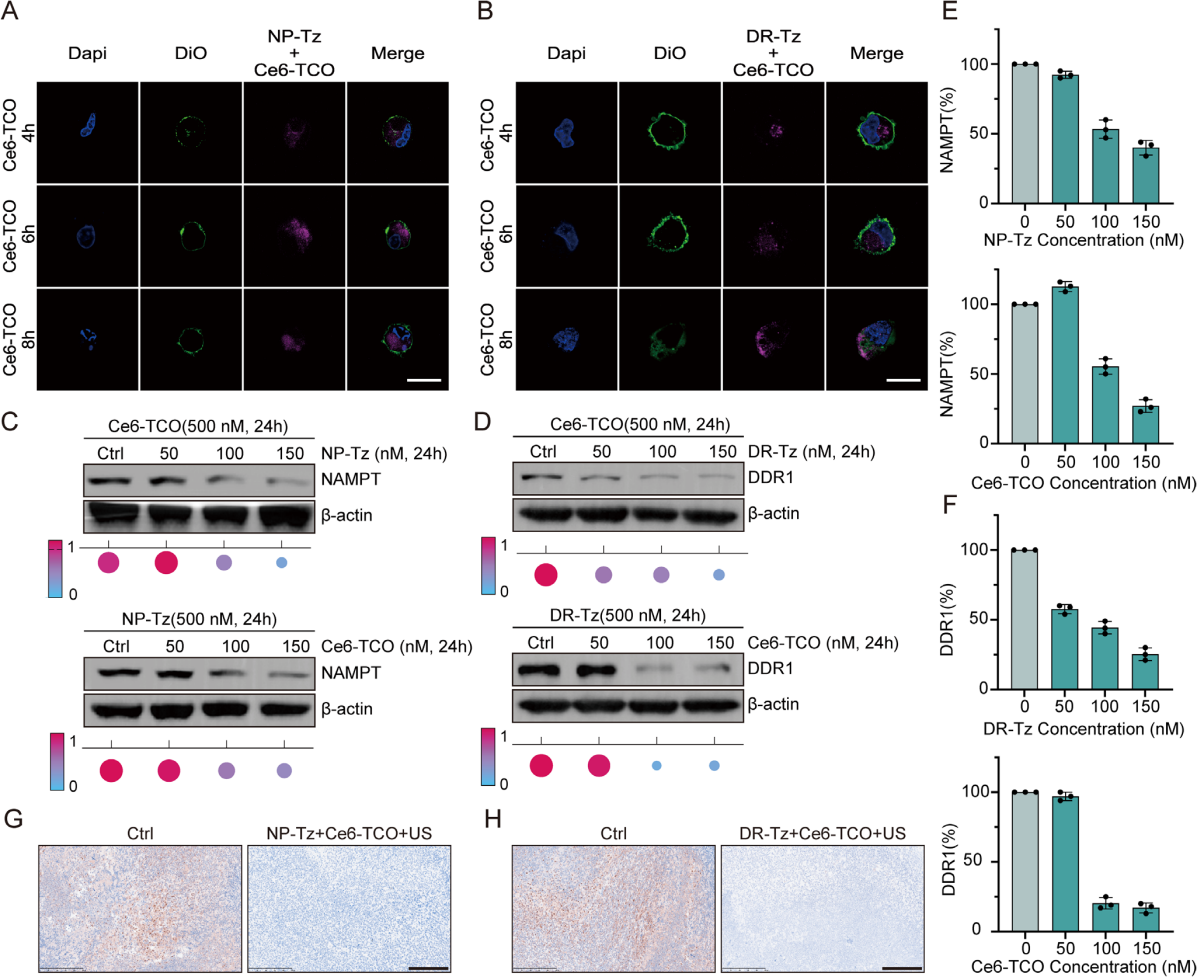
The scope of SDPTAC‐mediated degradation of cytoplasmic and membrane proteins. (A) Confocal fluorescence images show that after pre‐treatment with **NP‐Tz** (500 nM), **Ce6‐TCO** (500 nM) gradually localizes to the cytoplasm over time in HCT116 cancer cells. Scale bar = 20 µm. (B) Confocal fluorescence images show that after pre‐treatment with **DR‐Tz** (500 nM), **Ce6‐TCO** (500 nM) gradually localizes to the cell membrane over time in NCI‐H23 cancer cells. Scale bar = 20 µm. (C) Study of SDPTAC‐mediated degradation activity of NAMPT in the cytoplasm, n = 3. (D) Study of SDPTAC‐mediated degradation activity of DDR1 protein on the cell membrane, n = 3. (E) Quantitative analysis of NAMPT degradation in HCT116 cancer cells, n = 3, ^*^ *p*< 0.05, ^**^ *p*< 0.01. (F) Quantitative analysis of DDR1 degradation in NCI‐H23 cancer cells. n = 3, ^*^ *p*< 0.05, ^**^ *p*< 0.01, ^***^ *p*< 0.001. (G) NAMPT protein degradation in HCT116 xenograft tumor tissues. Scale bar = 250 µm. (H) DDR1 protein degradation in NCI‐H23 xenograft tumor tissues. Scale bar = 250 µm.

To further validate the versatility of the SDPTAC platform across different subcellular compartments, we extended the strategy to degrade cytosolic and membrane‐bound proteins. Two Tz‐conjugated ligands were synthesized: NP‐Tz targeting NAMPT and DR‐Tz targeting DDR1. Upon undergoing bioorthogonal IEDDA reactions with the Ce6‐TCO sonosensitizer, these ligands enabled localized ROS generation and selective degradation of the respective targets. Confocal laser scanning microscopy revealed that upon 24 h preincubation of cells with NP‐Tz, subsequent addition of Ce6‐TCO and an 8 h incubation led to the colocalization of the fluorescence signal within the cytoplasm, indicating successful intracellular bioorthogonal conjugation (Figures 6A,B; S6). A similar pattern was observed in cells treated with DR‐Tz followed by Ce6‐TCO, where the assembled chimera localized predominantly at the plasma membrane, confirming effective spatial targeting via SDPTAC.

To assess the degradation efficacy of SDPTAC against NAMPT and DDR1, Western blot analyses were conducted. For cytosolic target NAMPT, when cells were pretreated with 500 nM Ce6‐TCO for 24 h followed by escalating concentrations of NP‐Tz, notable protein degradation was observed at a NP‐Tz concentration of 100 nM. Conversely, when NP‐Tz was administered first (500 nM, 24 h), effective NAMPT degradation was also observed at 100 nM Ce6‐TCO (Figures 6C,E; S7), indicating that for cytosolic targets, the sequence of reagent addition does not critically affect degradation efficacy. For membrane‐localized DDR1, a different pattern emerged. Pretreatment with Ce6‐TCO followed by a gradient of DR‐Tz led to significant DDR1 degradation at 50 nM of DR‐Tz. However, when the administration sequence was reversed (500 nM DR‐Tz followed by Ce6‐TCO), degradation occurred at a higher Ce6‐TCO concentration of 100 nM (Figures 6D,F; S8). The result indicates that pre‐treatment with Ce6‐TCO leads to a higher local concentration at the membrane, thereby markedly enhancing the membrane‐proximal recruitment of subsequently added DR‐Tz and facilitating more efficient ROS‐mediated DDR1 degradation. To further confirm the applicability of SDPTAC in vivo, we evaluated NAMPT and DDR1 degradation in tumor‐bearing mice following three intraperitoneal administrations of NP‐Tz or DR‐Tz, Ce6‐TCO, and US irradiation. Immunoblotting of tumor lysates revealed marked degradation of both NAMPT and DDR1 proteins (Figure 6G,H), demonstrating the robust protein knockdown capabilities of SDPTAC in live animal models.

Collectively, these results highlight the broad utility of SDPTAC in selectively degrading proteins located in distinct subcellular compartments, including the cytosol, nucleus, and plasma membrane, through modular ligand design and US‐triggered ROS generation. This versatility positions SDPTAC as a powerful and adaptable platform for spatially precise, multi‐level targeted protein degradation.

## 3 Conclusion

In summary, we have established SDPTAC as a versatile platform that couples bioorthogonal IEDDA chemistry with US‐triggered sonodynamic therapy to achieve spatiotemporally precise protein degradation. By uniting a universally applicable Ce6‐TCO sonosensitizer with Tz‐tagged ligands, we created a modular “plug‐and‐play” kit that can be rapidly re‐engineered to degrade nuclear (BRD4), cytosolic (NAMPT), and membrane (DDR1) proteins. Mechanistic studies confirmed that intracellular IEDDA ligation and ROS production are both indispensable for protein degradation. In an orthotopic MDA‐MB‐231 breast cancer model, BRD4 SDPTAC achieved 94.2 % tumor growth inhibition with negligible systemic toxicity. These findings highlight SDPTAC's versatility and efficacy across diverse protein targets and cellular localizations. By leveraging US for precise activation, SDPTAC overcomes the penetration constraints of light‐based systems, significantly enhancing the spatiotemporal control of TPD. Together, SDPTAC serves as a powerful, deep‐penetrating, and broadly adaptable degradation technology, opening a path toward non‐invasive, US‐activated therapies for solid tumors and other protein‐driven diseases.

## 4 Experimental Section

### 4.1 Synthesis and Structural Characterization of Intermediates and Target Compounds

All starting materials were commercially available and analytically pure. ^1^H‐NMR and ^13^C‐NMR spectra were recorded on a Bruker AVANCE II 600 spectrometer (Bruker Company, Germany), using TMS as an internal standard and DMSO‐*d*_6_ as solvents. Chemical shifts (*δ* values) and coupling constants (*J* values) are given in ppm and Hz, respectively. The mass spectra were recorded on an Esquire 3000 LC‐MS mass spectrometer. Silica gel thin‐layer chromatography was performed on precoated plates GF‐254 (Qingdao Haiyang Chemical, China). Silica gel column chromatography was performed with Silica gel 60G (Qingdao Haiyang Chemical, China). Purity of the compound was analyzed by HPLC (Agilent Eclipse Plus C18 column, 5 µM, 4.6 × 250 mm) with a H_2_O/MeCN gradient containing 0.1 % trifluoroacetic acid and a UV detector set at the wavelength of 254 nm, and final compounds exhibited the purity greater than 95 %.

### 4.2 Assessment of Medium Stability

The compounds were diluted to 1.0 mM and incubated with DMEM medium supplemented with 10 % fetal bovine serum (FBS). At designated time points, 20 µL aliquots were collected and analyzed by HPLC.

### 4.3 Flow Cytometry Analysis of Intracellular ROS Levels

MDA‐MB‐231 cells were trypsinized, resuspended in complete medium, and seeded into 6‐well plates at a density of 1.5 × 10⁵ cells per well (2 mL medium per well). After overnight incubation to allow cell attachment, the medium was removed, and cells were gently washed with PBS. Cells were then treated with 500 nM **Ce6‐TCO** for 8 h, followed by ultrasound irradiation under the indicated conditions. After irradiation, cells were incubated for an additional 2 h. To assess intracellular ROS levels, 1 mL of 10 µM DCFH‐DA (Beyotime, S0033S) was added to each well and incubated at 37°C for 20 min. Subsequently, cells were washed three times with PBS, digested with trypsin, and the digestion was quenched by adding an equal volume of complete medium. The cell suspension was collected into 1.5 mL tubes, centrifuged at 1000 rpm for 5 min, washed once with PBS, resuspended in PBS, and analyzed using a flow cytometer (Beckman Coulter, model A00‐1‐1102). Fluorescence intensity was quantified using FlowJo software (v10.8.1).

### 4.4 Western Blot Analysis of Protein Degradation

MDA‐MB‐231, HCT‐116, and NCI‐H23 cells were seeded in 6‐well transparent culture plates (Corning, USA) at 1.5 × 10⁵ cells/well and cultured overnight at 37°C/5 % CO_2_. Attached cells were washed with ice‐cold PBS and treated with compounds (**JQ1‐Tz**, **NP‐Tz**, **DR‐Tz**) for 24 h. After removal of medium and three PBS washes, **Ce6‐TCO** was added and incubated for 8 h prior to ultrasound treatment, followed by continued incubation for a total of 24 h. After incubation, cells were washed with PBS, and RIPA lysis buffer (Beyotime, P0013B) containing protease and phosphatase inhibitors (Beyotime, P1045) was added, and the mixture was incubated for 15 min. The cells were collected into a centrifuge tube, vortexed every 5 min for a total of three times, and then centrifuged at 12 000 rpm for 15 min at 4°C. The supernatant was collected, and protein quantification was performed using a BCA protein assay kit (Beyotime, P0010). The samples were mixed with loading buffer (Epizyme, LT101) and boiled. Proteins were separated by 10 % sodium dodecyl sulfate‐polyacrylamide gel electrophoresis (SDS‐PAGE) and transferred to a 0.45 µm polyvinylidene fluoride (PVDF) membrane (Millipore, IPFL00010). The membrane was blocked with a protein‐free rapid blocking buffer (Epizyme, PS108P) for 30 min. The anti‐BRD4 primary antibody (Abcam, 128874), the anti‐Visfatin primary antibody (Abcam, ab236874), the anti‐DDR1 primary antibody (CST, 5583T), and the anti‐β‐actin antibody (Proteintech, 66009‐1‐Ig) were added and incubated overnight at 4°C. After recovering the primary antibody, the membrane was washed with TBST. A secondary antibody (Proteintech, SA00001) was added and incubated at room temperature for 1 h. Protein bands were visualized using an ECL detection kit (Epizyme, SQ101L) and analyzed with ImageJ. Data were presented as mean ± SD, analyzed with GraphPad Prism 9.4.

### 4.5 ROS Scavenging Assay

MDA‐MB‐231 cells were seeded in 6‐well clear flat‐bottom plates (Corning, USA) at a density of 1.5 × 10⁵ cells per well and cultured overnight at 37°C in a humidified atmosphere containing 5 % CO_2_. After cell attachment, the monolayers were gently washed once with ice‐cold PBS and treated with **JQ1‐Tz** for 24 h. The medium was then removed, and cells were washed three times with PBS, followed by incubation with **Ce6‐TCO** for 6 h. Subsequently, 10 mM N‐acetylcysteine (NAC) was added and incubated for 2 h prior to ultrasound irradiation. After irradiation, cells were further incubated until the total treatment duration reached 24 h. Protein expression levels were finally analyzed by Western blotting.

### 4.6 Orthogonal in vitro Experiment

**JQ1‐Tz** and **CE6‐TCO** were individually diluted to 500 nM in DMEM medium, mixed, and incubated on an orbital shaker at 80 rpm for 1 h. The mixture was then administered to cells. After 8 h of treatment, cells were exposed to ultrasound irradiation and further incubated until 24 h post‐treatment initiation.

### 4.7 Immunofluorescence Analysis of Spatiotemporal Location

MDA‐MB‐231, HCT‐116, and NCI‐H23 cells were seeded in confocal dishes (MatTek, USA) at 1 × 10⁵ cells/dish and cultured overnight at 37°C/5 % CO_2_. Attached cells were treated with **JQ1‐Tz**, **NP‐Tz**, and **DR‐Tz** (500 nM) for 24 h. After PBS (pH 7.4) washing, cells were incubated with **Ce6‐TCO** for 4, 6, or 8 h, washed thrice, and stained with 5 µM DiO (Beyotime, C1038) for 15 min in the dark. Following three PBS washes, cells were fixed with 4 % paraformaldehyde (Beyotime, P0099‐100 mL) for 15 min at RT, washed, and nuclei‐stained with 1 µg/mL DAPI (Beyotime, P0131) for 15 min. After final washes, samples were mounted in anti‐fade medium and imaged under a confocal microscope (Olympus, FV3000) using 63 × oil immersion.

### 4.8 Immunofluorescence Analysis of Protein Degradation

MDA‐MB‐231 cells were seeded at 4 × 10^4^ cells/well into confocal dishes, **JQ1‐Tz** (500 nM) was added, and incubated for 24 h. After removal of medium and three PBS washes, **Ce6‐TCO** was added and incubated for 8 h prior to ultrasound treatment, followed by continued incubation for a total of 24 h. Cells were washed with PBS, fixed with 4 % paraformaldehyde (Beyotime, P0099‐100 mL), blocked with 10 % goat serum (BOSTER, AR1009), incubated with primary antibodies overnight at 4°C, followed by fluorescent secondary antibodies (HUABIO, HA1122). Nuclei were stained with DAPI. Images were obtained by confocal microscopy.

### 4.9 Flow Cytometry Analysis of ROS Level

MDA‐MB‐231, HCT‐116, and NCI‐H23 cells were seeded in 6‐well transparent culture plates (Corning, USA) at 1.5 × 10⁵ cells/well and cultured overnight at 37°C/5 % CO_2_. Attached cells were washed with ice‐cold PBS and treated with compounds (**JQ1‐Tz**, **NP‐Tz**, **DR‐Tz**) for 24 h. After removal of medium and three PBS washes, **Ce6‐TCO** was added and incubated for 8 h prior to ultrasound treatment, followed by continued incubation for a total of 24 h. After incubation, the cells were digested and harvested. A total of 1 mL of Foxp3 Fixation/Permeabilization working solution (Thermo Fisher, 00–5523) was added to each tube and vortexed briefly. The solution was incubated for 30–60 min at 2°C–8°C or room temperature, protected from light. A total of 2 mL of 1X Permeabilization Buffer (Thermo Fisher, 88‐8824‐00) was added to each tube, then centrifuged at 500 × g for 5 min at room temperature. This wash step was repeated twice. The pellet was resuspended in the remaining volume of 1X Permeabilization Buffer. A total of 2 µL of 2 % goat serum blocking solution was directly added to the cells and incubated for 15 min at room temperature. Without washing, fluorochrome‐conjugated antibodies targeting intracellular antigens were added and incubated for ≥ 30 min at room temperature in the dark. A total of 2 mL of 1× Permeabilization Buffer was added to each tube and centrifuged at 500 × g for 5 min. The supernatant was discarded. Repeat the washing procedure (adding 2 mL buffer + centrifugation) twice. Resuspend stained cells in an appropriate volume of Flow Cytometry Staining Buffer (Thermo Fisher, 00–4222) and analyze by flow cytometry.

### 4.10 Proteome Assay

MDA‐MB‐231 cells in good condition were digested, and 9 × 10^7^ cells were seeded in nine cell‐culture dishes for 24 h. Then, **JQ1‐Tz** + **Ce6‐TCO** + **US**‐treated and **JQ1‐Tz** + **Ce6‐TCO‐**treated control groups were incubated with cells, respectively. Repeat 3 groups for each compound. After incubation, cells were washed with PBS and then lysed with RIPA lysis buffer on ice for 30 min. Total cell protein was obtained from the supernatant collected by centrifuging the cell lysate (12 000 g, 4°C). Subsequently, the cell lysate (100–200 µg) was sent to OEBiotech company for proteomic assay. Briefly, cellular samples were processed to extract total proteins, a portion of which was utilized for protein concentration determination and SDS‐PAGE analysis. Another portion was subjected to trypsin digestion and labeling, followed by equal mixing of the labeled samples for chromatographic separation. Subsequently, the samples were subjected to LC‐MS/MS analysis, and the acquired data were subjected to comprehensive data analysis.

### 4.11 Bioinformatical Analysis

Following protein identification and quantification using Proteome Discovery v1.4 software, we obtained the expression profiles of proteins in each sample. The first and foremost step involved checking data quality through Pearson correlation analysis. In our study, each experimental group was replicated three times, and data were processed by filtering out outliers using Mean Absolute Differences (MAD) and imputing missing values through a random forest‐based algorithm. After consolidating the data, we employed linear models (limma v3.52.4 R package) to analyze significantly differentially expressed proteins between the two groups (The above experimental procedures were conducted by OEBiotech company). Volcano plots, expression pattern clustering heatmaps, Venn analysis, and Gene Set Enrichment Analysis (GSEA) were employed for differential comparison group data. To gain a deeper understanding of differentially expressed proteins in the target KEGG pathway, we focused on the MAPK signaling pathway and integrated it with proteins of interest. Lastly, we conducted enrichment analysis for the proteins of interest using R packages cluster Profiler v4.4.4 and org.Hs.eg.db v3.15.0.

### 4.12 In vivo Therapeutic Efficacy

All the animal protocols were assessed and approved by the Committee on Ethics of Medicine, Navy Medical University (SMMU82030105). BALB/C nude female mice (certificate SCXK‐2021‐0013, weighing 17–20 g) were obtained from Changzhou Cavens Experimental Animal Co., Ltd. MDA MB 231 cells (6 × 10⁶ cells per mouse) were subcutaneously injected into the right flank. When the tumor volume reached approximately 120 ± 10 mm^3^, the mice were randomly divided into four groups (n = 6 per group): (1) **JQ1‐Tz** + **Ce6‐TCO**; (2) **JQ1‐Tz + Ce6‐TCO** + Ultrasound; (3) Ultrasound control; and (4) PBS control. Treatment groups received intraperitoneal injections of **JQ1‐Tz** (10 mg/kg in PBS) and an equivalent dose of **Ce6‐TCO** every other day. For the ultrasound treatment group, tumor sites were subjected to ultrasound irradiation (1 MHz, 1.0 W/cm^2^, 50 % duty cycle, 2 min) 8 h after **Ce6‐TCO** administration. The ultrasound control group received the same ultrasound exposure every 48 h, while control mice were administered an equal volume of PBS (100 µL per 20 g body weight). Tumor length (A) and width (B) were measured every two days, and tumor volumes were calculated using the formula *V = AB^2^/2*. Body weight was recorded simultaneously. On day 16, the mice were euthanized, and tumors were excised, photographed, and weighed. Major organs (heart, liver, spleen, lungs, and kidneys) were collected, weighed, and fixed in 4 % paraformaldehyde (PFA) for histological analysis. Data are presented as mean ± SD and analyzed using one‐way ANOVA followed by Tukey's post hoc test in GraphPad Prism 9.0. Statistical significance was defined as *p* < 0.05.

### 4.13 Hematoxylin‐Eosin Staining and Histology

After 14 days post‐treatment, the heart, liver, spleen, lung, kidney, and tumors of all treatment groups were dissected and fixed with 4 %paraformaldehyde. The tissue of organs was sliced and stained by Bios Biological Company.

### 4.14 Statistical Analysis

All data are presented as mean ± standard deviation (SD), and each experiment was repeated at least three times. Statistical significance was evaluated using Student's *t*‐test or one‐way analysis of variance (ANOVA), as appropriate. A *p*‐value of < 0.05 was considered statistically significant. All analyses were performed using GraphPad Prism software.

## Conflicts of Interest

The authors declare no conflict of interest.

## Supporting information




**Supporting File**: advs73519‐sup‐0001‐SuppMat.docx.

## Data Availability

The data that support the findings of this study are available in the supplementary material of this article.
