# Supplementary material for: Bioorthogonal Sonodynamic Plug‐and‐Play Targeting Chimeras (SDPTAC) for Precise Targeted Protein Degradation
Source: Adv Sci (Weinh). 2025 Dec 22;13(10):e20975. doi: 10.1002/advs.202520975 (PMC12915185; doi:10.1002/advs.202520975)
Supplement: Supplementary file 1 — Supporting File: advs73519‐sup‐0001‐SuppMat.docx. [file ADVS-13-e20975-s001.docx]

**Supporting information**

**Bioorthogonal Sonodynamic Plug-and-Play Targeting Chimeras (SDPTAC) for Precise Targeted Protein Degradation**

Yuhan Bao^†1^, Yaojin Zhu^†1^, Xinhao Wei^†1^, Yuxin Fang^2^, Jiayi Zhu^1^, Fei Gao^1^, Guoqiang Dong^*2^, Shipeng He^*1^ and Chunquan Sheng^*2^

*^1^ School of Medicine or Institute of Translational Medicine, Shanghai University, 99 Shangda Road, Shanghai 200444, P.R. China.*

*^2^ The Center for Basic Research and Innovation of Medicine and Pharmacy (MOE), School of Pharmacy, Second Military Medical University (Naval Medical University), 325 Guohe Road, Shanghai 200433, P.R. China*

^*^Correspondence:

[gdong@smmu.edu.cn](mailto:gdong@smmu.edu.cn); heshipeng@shu.edu.cn; shengcq@hotmail.com.

^†^These authors contributed equally to this work.

Table of Contents

[Figure S1. Quantitative analysis of Flow cytometry of Relative Level of ROS in MDA-MB-231 cells S1](#_Toc215686800)

[Figure S2. Western blot analysis of BRD4 protein degradation in MDA-MB-231 cells treated with **JQ1-Tz**+**Ce6-TCO**+US at different time point S1](#_Toc215686801)

[Figure S3. Western blot analysis of BRD4 protein degradation in MDA-MB-231 cells treated with **JQ1-Tz**+**Ce6-TCO**+US of different concentration S1](#_Toc215686802)

[Figure S4. Uncropped blot corresponding to **Figure 3**. S2](#_Toc215686803)

[Figure S5. Cytotoxicity assays using human breast cancer cells MDA-MB-231 and normal human breast epithelial cells MCF-10A, respectively S2](#_Toc215686804)

[Figure S6. Three-dimensional fluorescence images showing the spatial accumulation of **Ce6-TCO**. S3](#_Toc215686805)

[Figure S7. Uncropped blot corresponding to **Figure 6**. S3](#_Toc215686806)

[Figure S8. Uncropped blot corresponding to **Figure 6**. S4](#_Toc215686807)

[Materials and Methods S5](#_Toc215686808)

[The spectra of the synthesized compounds S23](#_Toc215686809)


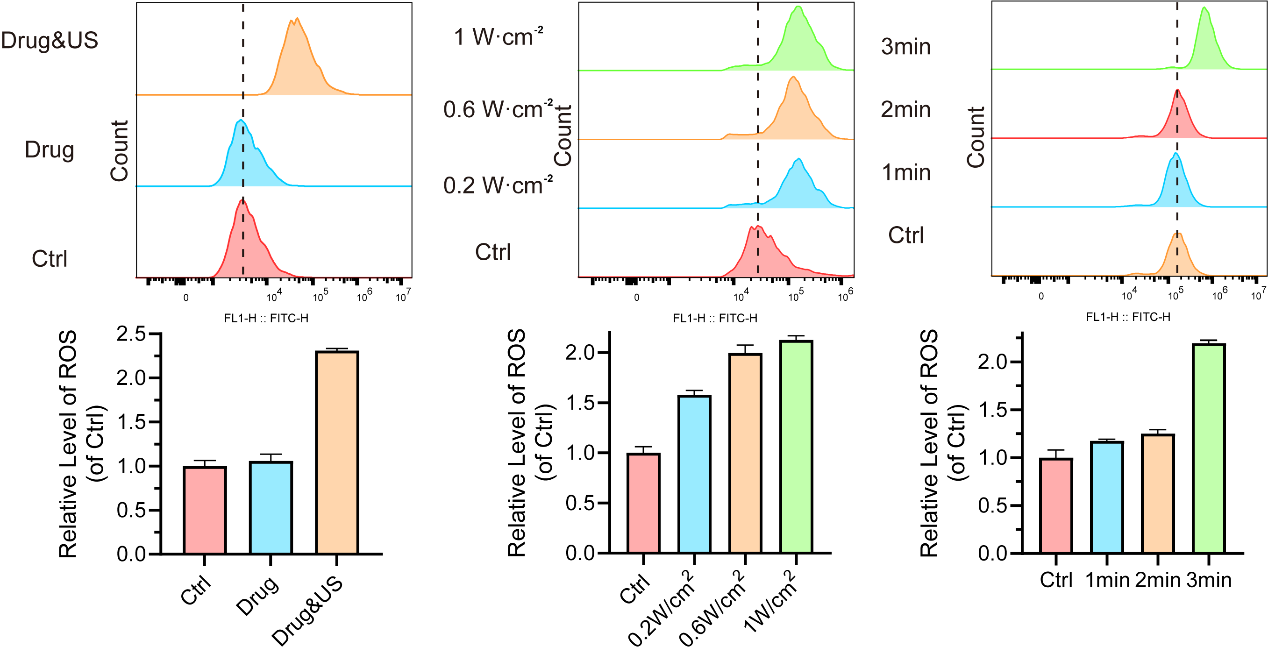


**Figure S1.** Quantitative analysis of Flow cytometry of Relative Level of ROS in MDA-MB-231 cells treated with **Ce6-TCO** at different power or processing time, n = 3.


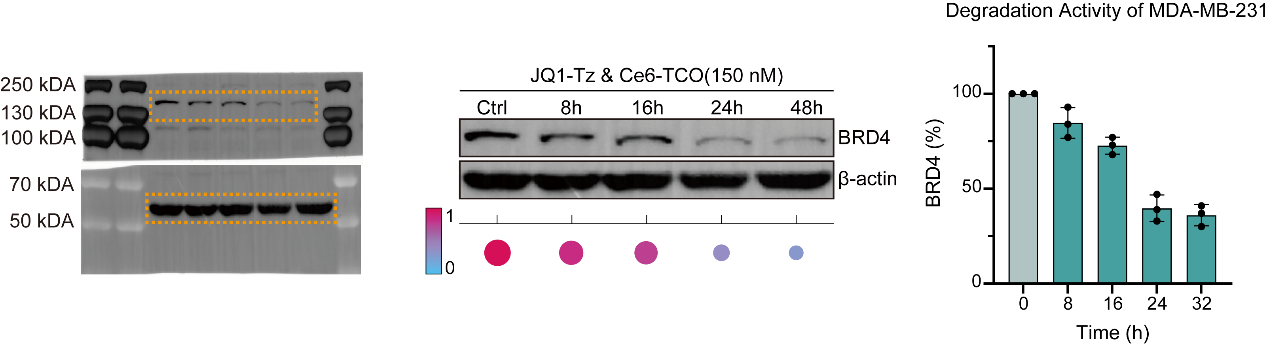


**Figure S2.** Western blot and quantitative analysis of BRD4 protein degradation in MDA-MB-231 cells treated with **JQ1-Tz**+**Ce6-TCO**+US at different time point, n = 3.


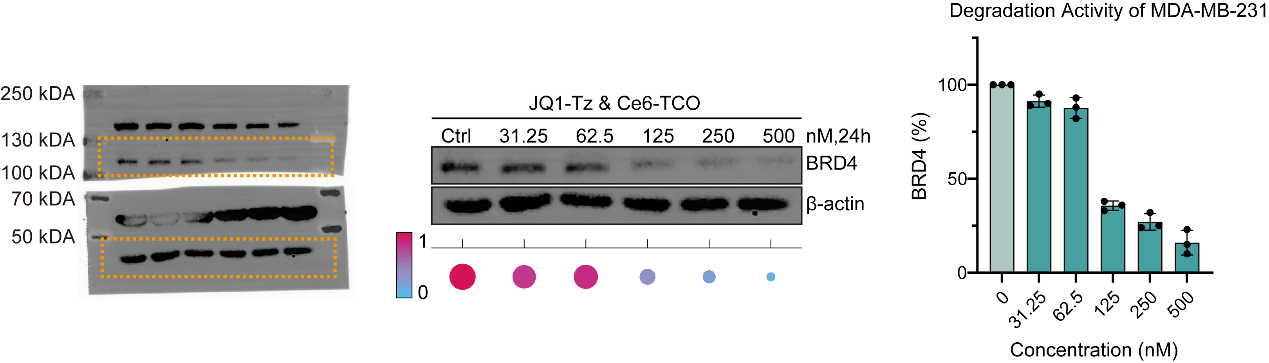


**Figure S3.** Western blot and quantitative analysis of BRD4 protein degradation in MDA-MB-231 cells treated with **JQ1-Tz**+**Ce6-TCO**+US of different concentration, n = 3.


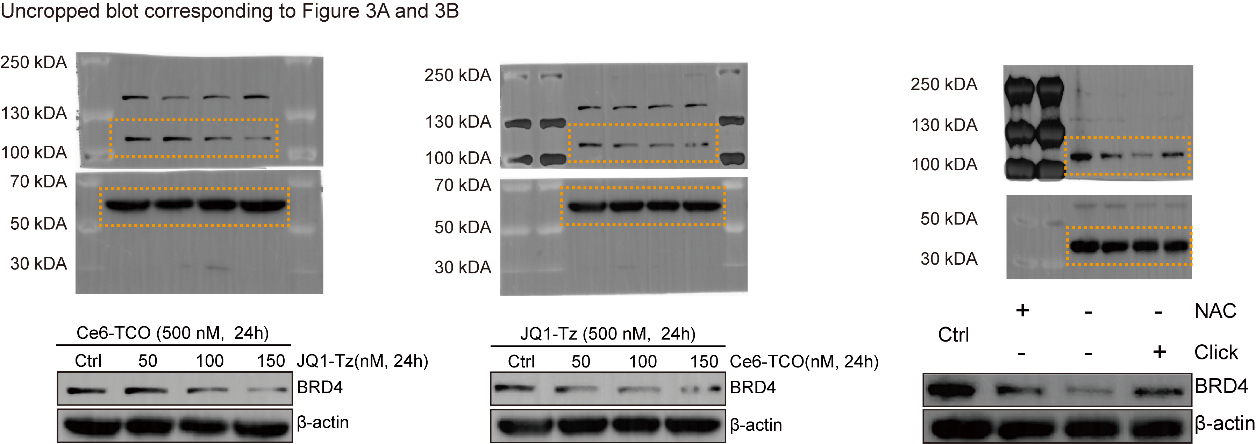


**Figure S4.** Uncropped blot corresponding to **Figure 3**.


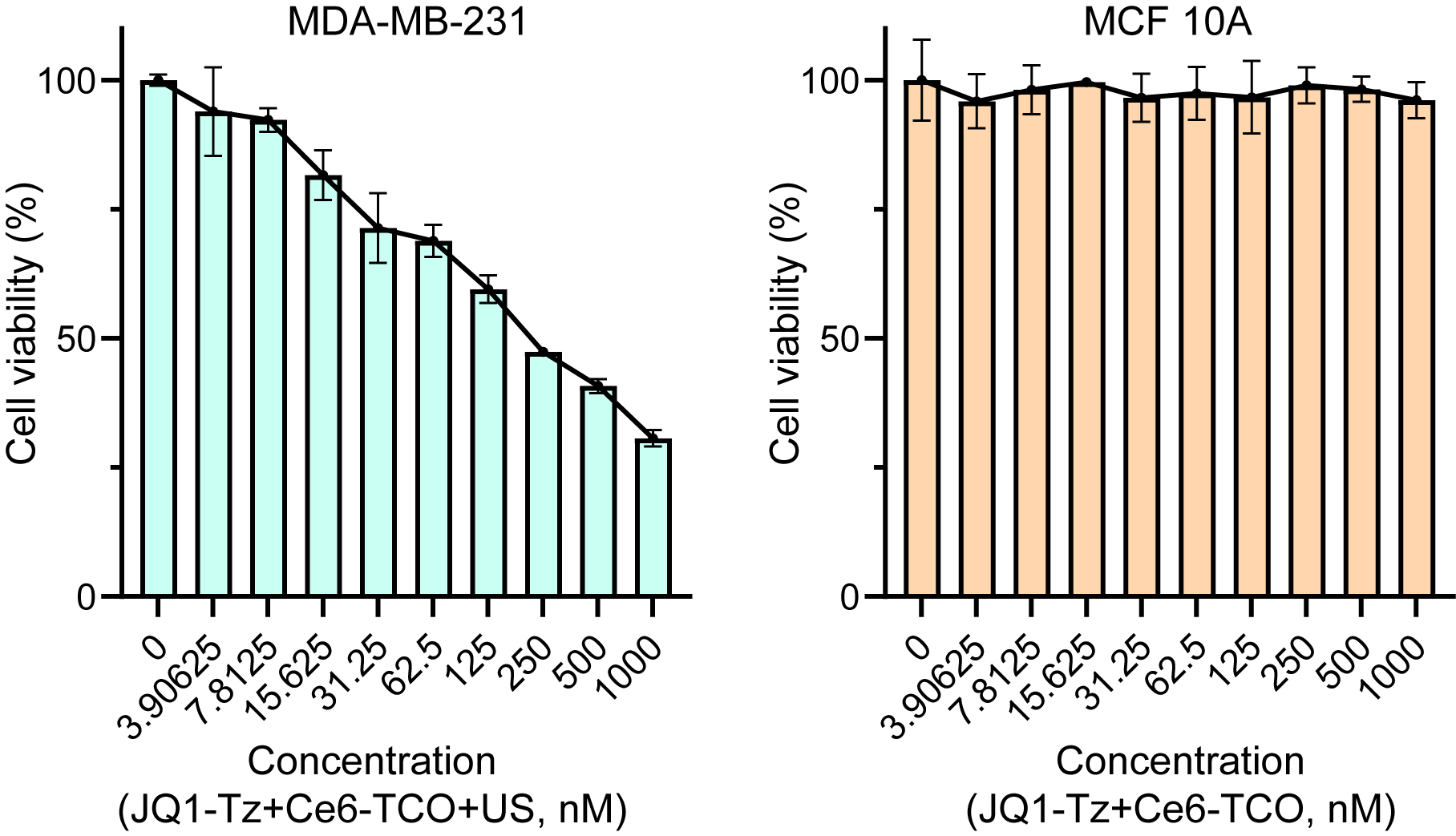


**Figure S5.** Cytotoxicity assays using human breast cancer cells MDA-MB-231 and normal human breast epithelial cells MCF-10A, respectively, n = 3.

**
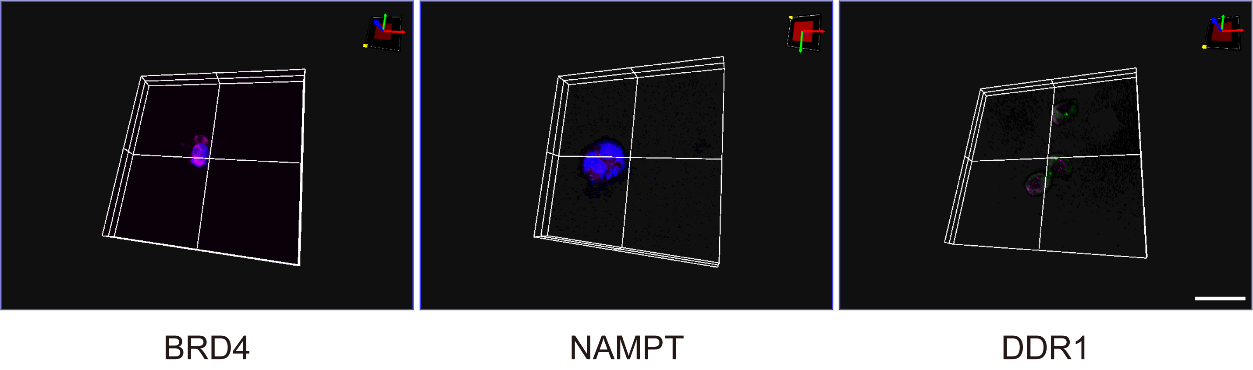
**

**Figure S6.** Three-dimensional fluorescence images showing the spatial accumulation of **Ce6-TCO** in MDA-MB-231, HCT-116, and NCI-H23 cells. Cells were pretreated with **JQ1-Tz**, **NP-Tz**, or **DR-Tz** for 24 h, followed by Ce6-TCO incubation for 8 h. Scale bar = 20 μm.


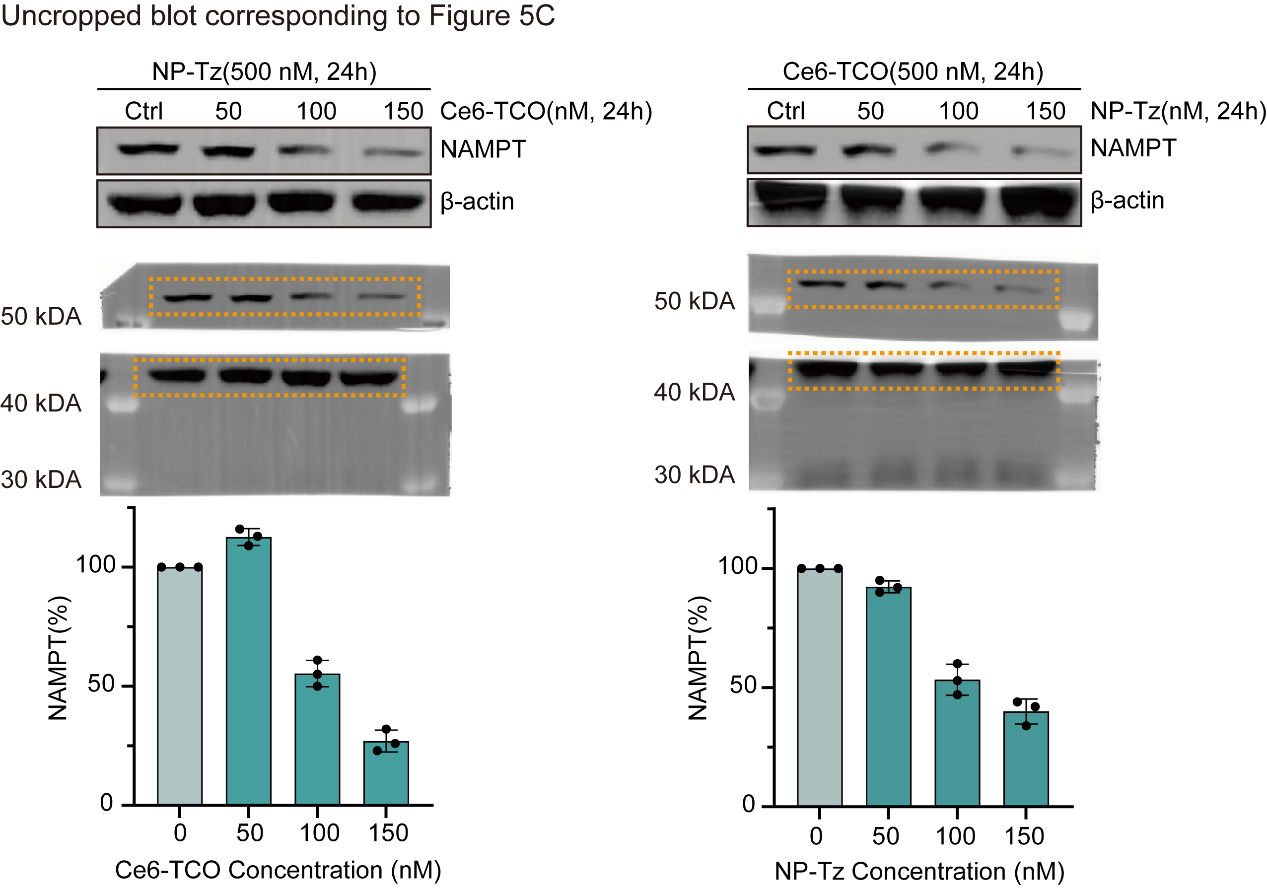


**Figure S7.** Uncropped blot corresponding to **Figure 6**.


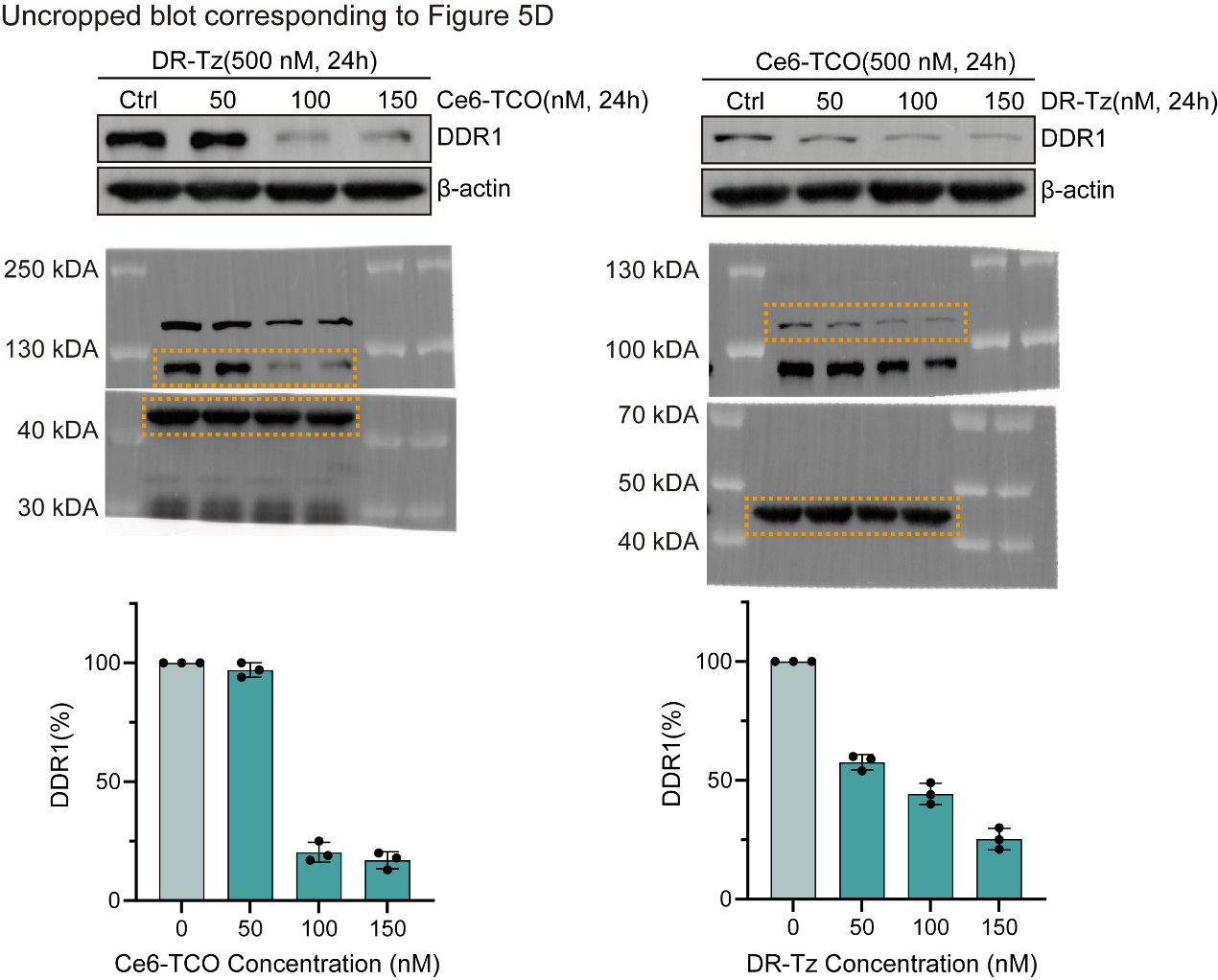


**Figure S8.** Uncropped blot corresponding to **Figure 6**.

**Materials and Methods**

**Chemistry**

**Scheme S1**. Synthesis of compound **5**.

**Reagents and conditions:** (a) TEA, DCM, r.t.; (b) Pd/C, H_2_, DCM, r.t.; (c) triphosgene, 3-(aminomethyl) pyridine, TEA, DCM, r.t.; (d) 1M HCl/EA solution, r.t..

**Scheme S2** Synthesis of compound **6**

**Reagents and conditions:** (a) borane tetrahydrofuran complex, THF, 65°C; carbon tetrabromide, triphenylphosphine, DCM, r.t.; (c) *tert*-butyl-1-piperazinecarboxylate, TEA, DCM, r.t.; (d) Pd/C, H_2_, DCM, r.t.; (e) i trimethylsilyl acetylene, PdCl_2_(PPh_3_)_2_, CuI, TEA, CH_3_CN, 60°C; ii K_2_CO_3_, MeOH, r.t., (two steps); (f) 6-bromopyrazolo[1,5-*a*] pyrimidine, CuI, PdCl_2_(PPh_3_)_2_, DIPEA, DMF, 80℃; (g) NaOH, MeOH, H_2_O, 50 °C; (h) HATU, DIPEA, DMF, 80 °C.

**NMR (^1^H-NMR and ^13^C-NMR) and HRMS data**

*(7S,8S)-5-(carboxymethyl)-7-(3-((3-(((((E)-cyclooct-4-en-1-yl)oxy)carbonyl)amino)propyl)amino)-3-oxopropyl)-18-ethyl-2,8,12,17-tetramethyl-13-vinyl-7H,8H porphyrin-3-carboxylic acid* (**3**)*.* Compound **Ce6** (**1**, 100 mg, 0.168 mmol) was dissolved in 10 ml DMF, and then compound TCO (**2**, 37.9 mg, 0.168 mmol), EDCI (38.6 mg, 0.201 mmol), HOBT (29.5 mg, 0.218 mmol), and DIPEA (65.0 mg, 0.503 mmol) were added. The reaction was carried out under N_2_ protection at room temperature for 4 h, and the reaction progress was monitored by HPLC. After the reaction was completed, the sample was purified by HPLC, and the product (**3**) was obtained in 110.5 mg. Black solid (yield 81.9%). ^1^H NMR (600 MHz, DMSO-*d*_6_) δ 9.95 (s, 1H), 9.84 (s, 1H), 9.23 (s, 1H), 8.34 (dd, *J* = 17.8, 11.6 Hz, 1H), 7.76 (s, 1H), 6.82 (d, *J* = 5.9 Hz, 1H), 6.46 (d, *J* = 17.8 Hz, 1H), 6.21 (d, *J* = 11.6 Hz, 1H), 5.55 (t, *J* = 8.5 Hz, 1H), 5.52 – 5.45 (m, 1H), 5.32 (d, *J* = 18.2 Hz, 1H), 5.17 (d, *J* = 18.2 Hz, 1H), 4.61 (q, *J* = 7.3 Hz, 1H), 4.42 (d, *J* = 9.8 Hz, 1H), 4.40 – 4.30 (m, 1H), 3.86 (dp, *J* = 13.6, 6.5, 5.8 Hz, 2H), 3.58 (s, 3H), 3.53 (s, 3H), 3.33 (s, 3H), 3.13 – 3.05 (m, 1H), 3.06 – 2.97 (m, 1H), 2.93 (q, *J* = 7.0 Hz, 2H), 2.22 – 2.08 (m, 3H), 2.04 – 1.87 (m, 3H), 1.70 (d, *J* = 7.2 Hz, 3H), 1.65 (d, *J* = 14.6 Hz, 1H), 1.61 (t, *J* = 7.6 Hz, 5H), 1.52 (t, *J* = 6.4 Hz, 2H), 1.47 (d, *J* = 16.8 Hz, 1H), 1.41 – 1.32 (m, 2H), 1.31 – 1.13 (m, 2H), -2.05 (s, 2H); ^13^C NMR (151 MHz, DMSO-*d*_6_) δ 174.2, 172.3, 171.0, 169.4, 167.9, 155.8, 143.8, 139.6, 136.5, 135.2, 134.5, 131.2, 129.9, 129.5(3), 129.4(2), 129.2(2), 126.9, 122.7, 104.9, 100.8, 97.7, 95.1, 74.2, 52.9, 48.3, 37.6, 36.6, 33.6, 33.6, 31.0, 29.8, 29.6, 25.0, 25.0, 24.4, 24.4, 23.0, 21.8, 18.9, 17.4, 12.2, 11.0. HRMS (ESI^+^): m/z calcd. for C_46_H_56_N_6_O_7_ [M+H]^+^ 805.4284, found 805.4277. Retention time 31.60 min, Purity > 95%.

*1-(4-(Piperazin-1-ylsulfonyl)phenyl)-3-(pyridin-3-ylmethyl)urea* (**5**). Compound **S5** (1.00 g, 2.10 mmol) was dissolved in 10 ml of 1M HCl/EA solution and reacted at room temperature for 2 h. The reaction was monitored by TLC. After the reaction was completed, the sample was washed with saturated sodium bicarbonate solution and then concentrated under reduced pressure to afford a white solid (**5**) without purification. Compound 5 can then be directly utilized in the subsequent step. MS (ESI^+^): m/z calcd. for C_17_H_21_N_5_O_3_S [M+H]^+^ 376.14, found 376.20.

The synthesis of compound **6** was carried out in reference to the method described in *J. Med. Chem*. **2024**, 67(10), 8043-8059.

*4-Ethyl-N-(3-(piperazin-1-ylmethyl)-5-(trifluoromethyl)phenyl)-3-(pyrazolo[1,5-a] pyrimidin-6-ylethynyl)benzamide* (**6**)*.* White solid, yield 54.5%. ^1^H NMR (600 MHz, DMSO-*d*_6_) δ 10.75 (s, 1H), 9.45 (s, 1H), 9.22 (s, 1H), 8.64 (s, 1H), 8.28 (s, 1H), 8.21 (s, 1H), 8.18 (d, *J* = 4.2 Hz, 2H), 7.96 (d, *J* = 8.0 Hz, 1H), 7.56 (s, 1H), 7.49 (d, *J* = 8.1 Hz, 1H), 6.79 (s, 1H), 4.22 (s, 2H), 3.32 (t, *J* = 5.4 Hz, 4H), 3.18 (t, *J* = 5.4 Hz, 4H), 2.91 (q, *J* = 7.5 Hz, 2H), 1.25 (t, *J* = 7.5 Hz, 3H); ^13^C NMR (151 MHz, DMSO-*d*_6_) δ 165.3, 151.1, 150.3, 147.2, 146.1, 140.5, 138.3, 134.1, 132.3, 131.5, 130.2, 129.0, 128.9, 126.1, 124.1, 122.3, 121.4, 117.4, 105.1, 97.7, 90.8, 86.7, 59.6, 48.5(2), 41.6(2), 27.4, 14.8. MS (ESI^+^): m/z calcd. for C_29_H_27_F_3_N_6_O [M+H]^+^ 533.23, found 533.15.

*(S)-2-(4-(4-chlorophenyl)-2,3,9-trimethyl-6H-thieno[3,2-f][1,2,4]triazolo[4,3-a] [1,4]diazepin-6-yl)-N-(4-(6-methyl-1,2,4,5-tetrazin-3-yl)benzyl)acetamide* (**9**). Compound JQ1-COOH (**4**, 50.0 mg, 0.125 mmol) was dissolved in 10 ml of DCM, and compound TZ-NH_2_ (**7**, 25.1 mg, 0.125 mmol), EDCI (28.8 mg, 0.150 mmol), HOBT (22.0 mg, 0.163 mmol), and DIPEA (48.5 mg, 0.375 mmol) were added. The reaction was carried out under N_2_ protection at room temperature for 4 h, and the progress was monitored by TLC. After the reaction was completed, the mixture was washed with saturated sodium bicarbonate solution and then with saturated brine, dried over anhydrous sodium sulfate, and concentrated. The product was purified by silica gel column chromatography (DCM/MeOH = 20/1), yielding 65.64 mg of the product (**9**) (yield 90.1%). ^1^H NMR (600 MHz, Chloroform-*d*) δ 8.49 (d, *J* = 8.4 Hz, 2H), 7.54 (t, *J* = 6.1 Hz, 1H), 7.51 (d, *J* = 8.0 Hz, 2H), 7.31 (dt, *J* = 8.8, 7.5 Hz, 4H), 4.76 (td, *J* = 7.0, 1.3 Hz, 1H), 4.69 (dd, *J* = 15.3, 6.4 Hz, 1H), 4.51 (dd, *J* = 15.3, 5.6 Hz, 1H), 3.63 – 3.50 (m, 2H), 3.09 (s, 3H), 2.78 (s, 3H), 1.68 (s, 3H); ^13^C NMR (151 MHz, Chloroform-*d*) δ 170.3, 167.3, 165.3, 164.0, 155.1, 150.5, 142.9, 137.8, 135.4, 132.7, 131.6, 131.2, 131.2, 131.1, 130.1, 129.0, 128.6, 128.3, 54.0, 43.6, 37.9, 21.3, 14.5, 13.3, 11.5. HRMS (ESI^+^): m/z calcd. for C_29_H_26_ClN_9_OS [M+H]^+^ 584.1743, found 584.1736. Retention time 29.10 min, Purity > 95%.

*1-(4-((4-(2-(4-(6-methyl-1,2,4,5-tetrazin-3-yl)phenyl)acetyl)piperazin-1-yl)sulfonyl) phenyl)-3-(pyridin-3-ylmethyl)urea* (**10**)*.* Compound **5** (78.8 mg, 0.210 mmol), compound TZ-COOH (**8**, 45.5 mg, 0.210 mmol), EDCI (48.4 mg, 0.253 mmol), HOBT (37.0 mg, 0.274 mmol), and DIPEA (108 mg, 0.842 mmol) were added in 20 ml of DCM. The reaction was conducted under N_2_ protection at room temperature for 4 h, and the progress was monitored by TLC. After the reaction was complete, the mixture was washed with saturated sodium bicarbonate solution and then with saturated brine, dried over anhydrous sodium sulfate, and evaporated. The product was purified by silica gel column chromatography (DCM/MeOH = 20/1) to yield 106 mg of the product (**10**) (yield 85.7%). ^1^H NMR (600 MHz, DMSO-*d*_6_) δ 9.53 (s, 1H), 8.75 (d, *J* = 2.1 Hz, 1H), 8.70 (d, *J* = 5.3 Hz, 1H), 8.35 (d, *J* = 8.0 Hz, 2H), 8.23 (d, *J* = 8.0 Hz, 1H), 7.81 (dd, *J* = 8.0, 5.3 Hz, 1H), 7.64 (d, *J* = 8.6 Hz, 2H), 7.58 (d, *J* = 8.7 Hz, 2H), 7.42 (d, *J* = 8.0 Hz, 2H), 7.25 (t, *J* = 6.0 Hz, 1H), 4.45 (d, *J* = 5.8 Hz, 2H), 3.81 (s, 2H), 3.62 (t, *J* = 5.0 Hz, 2H), 3.56 (t, *J* = 5.2 Hz, 2H), 2.98 (s, 3H), 2.84 (q, *J* = 4.8 Hz, 4H); ^13^C NMR (151 MHz, DMSO-*d*_6_) δ 168.5, 167.0, 163.2, 154.9, 145.0, 143.7, 141.0, 140.6, 138.6, 130.4(2), 130.0, 128.9(2), 127.2(2), 126.0, 125.6, 125.6, 117.3(2), 46.0, 45.8, 44.5, 40.5, 40.3, 20.8(2). HRMS (ESI^+^): m/z calcd. for C_28_H_29_N_9_O_4_S [M+H]^+^ 588.2136, found 588.2122. Retention time 17.10 min, Purity > 95%.

*4-ethyl-N-(3-((4-(2-(4-(6-methyl-1,2,4,5-tetrazin-3-yl)phenyl)acetyl)piperazin-1yl) methyl)-5-(trifluoromethyl)phenyl)-3-(pyrazolo[1,5-a]pyrimidin-6-ylethynyl) Benzamide* (**11**)*.* Compound **6** (100 mg, 0.188 mmol) was added to a 10 mL DCM solution, followed by the addition of compound TZ-COOH (**8**, 40.6 mg, 0.188 mmol), EDCI (43.2 mg, 0.225 mmol), HOBT (33.0 mg, 0.244 mmol), and DIPEA (94.7 mg, 0.733 mmol). The reaction was carried out under N_2_ protection at room temperature for 4 h, and the progress was monitored by TLC. After the reaction was completed, the mixture was washed with saturated sodium bicarbonate solution and then with saturated brine. The organic phase was dried over anhydrous sodium sulfate and concentrated. The product was purified by silica gel column chromatography (DCM/MeOH = 20/1) to afford 115 mg of the product (**11**) (yield 82.3%). ^1^H NMR (600 MHz, DMSO-*d*_6_) δ 10.79 (s, 1H), 9.57 (s, 1H), 8.70 (t, *J* = 1.5 Hz, 1H), 8.42 (d, *J* = 8.3 Hz, 2H), 8.35 (d, *J* = 12.1 Hz, 2H), 8.20 (d, *J* = 15.6 Hz, 2H), 8.00 (d, *J* = 8.3 Hz, 1H), 7.66 (s, 1H), 7.57 (d, *J* = 8.1 Hz, 1H), 7.50 (d, *J* = 7.9 Hz, 2H), 6.85 (d, *J* = 2.3 Hz, 1H), 4.47 (s, 2H), 3.93 (s, 2H), 3.27 (d, *J* = 183.1 Hz, 7H), 2.99 (d, *J* = 1.2 Hz, 3H), 2.98 – 2.93 (m, 2H), 1.30 (td, *J* = 7.5, 1.2 Hz, 3H); ^13^C NMR (151 MHz, MeOD-*d*) δ 170.1, 167.4, 166.2, 163.7, 150.8, 150.6, 146.8, 145.9, 140.4, 139.4, 137.3, 131.9, 131.8, 131.3, 130.9, 130.5, 129.8(2), 128.4, 128.2, 127.7(2), 126.5, 123.6, 122.8, 121.7, 118.5, 105.1, 96.3, 89.7, 86.8, 59.5, 51.1, 48.2, 42.6, 39.2, 38.6, 27.4, 19.7, 14.1. HRMS (ESI^+^): m/z calcd. for C_40_H_35_F_3_N_10_O_2_ [M+H]^+^ 745.2970, found 745.2971. Retention time 29.40 min, Purity > 95%.

*tert-Butyl 4-((4-nitrophenyl)sulfonyl)piperazine-1-carboxylate* (**S3**). Compound **S1** (5.00 g, 22.6 mmol) and Compound **S2** (4.20 g, 22.6 mmol) were dissolved in 70 mL of DCM. Then the TEA (5 mL) was added at room temperature and the reaction was carried out for 0.5 h, monitored by TLC. After the reaction was completed, 1 M dilute hydrochloric acid was added to adjust the pH to weakly acidic, and the mixture was extracted with DCM. The extract was concentrated to give 8.02 g of white solid (**S3**) (yield 95.6%). ^1^H NMR (600 MHz, DMSO-*d*_6_) δ 8.43 (d, *J* = 8.5 Hz, 2H), 8.00 (d, *J* = 8.6 Hz, 2H), 3.40 (t, *J* = 5.0 Hz, 4H), 2.95 (t, *J* = 5.0 Hz, 4H), 1.33 (s, 9H); ^13^C NMR (151 MHz, DMSO-*d*_6_) δ 153.3, 149.7, 140.6, 129.1(2), 124.8(2), 79.3, 45.6(4), 27.9(3).

*tert-Butyl 4-((4-aminophenyl)sulfonyl)piperazine-1-carboxylate* (**S4**). Compound **S3** (4.00 g, 10.8 mmol) was dissolved in 50 ml of DCM/MeOH (4/1), and 10% Pd/C (1.15 g, 1.08 mmol) was added. The reaction was carried out under H_2_ protection at room temperature overnight. TLC indicated the reaction was complete. The mixture was filtered, and the filtrate was concentrated to afford 3.50 g white solid (**S4**) (yield 95.1%). ^1^H NMR (600 MHz, DMSO-*d*_6_) δ 7.33 (d, *J* = 8.3 Hz, 2H), 6.65 (d, *J* = 8.4 Hz, 2H), 6.12 (s, 2H), 3.36 (t, *J* = 5.1 Hz, 4H), 2.71 (t, *J* = 5.0 Hz, 4H), 1.34 (s, 9H); ^13^C NMR (151 MHz, DMSO-*d*_6_) δ 153.4, 153.4, 129.6(2), 118.8, 112.7(2), 79.3, 45.7(4), 27.9(3). MS (ESI^+^): m/z calcd. for C_15_H_23_N_3_O_4_S [M+Na]^+^ 364.13, found 364.20.

*tert-Butyl 4-((4-(3-(pyridin-3-ylmethyl)ureido)phenyl)sulfonyl)piperazine-1-carboxylate* (**S5**). Triphosgene (289 mg, 0.970 mmol) was dissolved in 5 mL DCM and stirred at 0°C. Compound **S4** (827 mg, 2.43 mmol) was dissolved in 7 mL DCM, and TEA (0.440 mL, 3.15 mmol) was added. The solution was then added to the DCM solution of phosgene trioxide and stirred at room temperature for 1 h. 3-Aminomethylpyridine (262 mg, 2.43 mmol) was dissolved in 5 mL DCM, and TEA (0.44 mL, 3.15 mmol) was added. The solution was then added to the above reaction mixture and stirred at room temperature for 2 h. The reaction was monitored by TLC. After the reaction was completed, the solvent was evaporated, and the product was purified by column chromatography with MeOH/DCM (1/20) to obtain 1.13 g white solid (**S5**) (yield 98.2%). ^1^H NMR (600 MHz, DMSO-*d*_6_) δ 9.65 (s, 1H), 8.81 (s, 1H), 8.76 (d, *J* = 5.5 Hz, 1H), 8.35 (dd, *J* = 8.0, 1.9 Hz, 1H), 7.91 (dd, *J* = 8.1, 5.5 Hz, 1H), 7.66 (d, *J* = 8.7 Hz, 2H), 7.58 (d, *J* = 8.6 Hz, 2H), 7.41 (dd, *J* = 5.5, 1H), 4.48 (d, *J* = 5.8 Hz, 2H), 3.37 (t, *J* = 5.1 Hz, 4H), 2.77 (t, *J* = 5.1 Hz, 4H), 1.33 (s, 9H); ^13^C NMR (151 MHz, DMSO-*d*_6_) δ 155.0, 153.4, 145.0, 142.5, 142.2, 139.4, 128.9(2), 126.2, 126.2, 126.0, 117.3(2), 75.7, 45.8(4), 40.2, 27.9(3). MS (ESI^+^): m/z calcd. for C_22_H_29_N_5_O_5_S [M+H]^+^ 476.20, found 476.20.

*(3-Nitro-5-(trifluoromethyl)phenyl)methanol* (**S7**). Yellow oil, yield 95.6%. ^1^H NMR (600 MHz, DMSO-*d*_6_) δ 7.56 (s, 1H), 7.44 (s, 1H), 7.23 (s, 1H), 4.85 (t, *J* = 5.7 Hz, 1H), 3.84 (d, *J* = 5.7 Hz, 2H); ^13^C NMR (151 MHz, DMSO-*d*_6_) δ 148.1, 147.3, 130.2, 128.7, 124.5, 123.2, 118.5, 61.3.

*1-(Bromomethyl)-3-nitro-5-(trifluoromethyl)benzene* (**S8**). Yellow oil, yield 65.2%. ^1^H NMR (600 MHz, DMSO-*d*_6_) δ 8.63 (s, 1H), 8.36 (s, 1H), 8.30 (s, 1H), 4.91 (s, 2H). ^13^C NMR (151 MHz, DMSO-*d*_6_) δ 148.2, 142.4, 132.0, 130.8, 127.8, 122.6, 119.8, 30.8.

*tert-Butyl 4-(3-nitro-5-(trifluoromethyl)benzyl)piperazine-1-carboxylate* (**S9**). White solid, yield 81.3%. ^1^H NMR (600 MHz, DMSO-*d*_6_) δ 8.45 (s, 1H), 8.36 (s, 1H), 8.13 (s, 1H), 3.73 (s, 2H), 3.33 (t, *J* = 6.5 Hz, 4H), 2.37 (t, *J* = 5.0 Hz, 4H), 1.38 (s, 9H); ^13^C NMR (151 MHz, DMSO-*d*_6_) δ 154.2, 148.7, 143.3, 131.8, 130.7, 127.5, 123.5, 119.5, 79.2, 60.4, 52.6(4), 28.4(3).

*tert-Butyl 4-(3-amino-5-(trifluoromethyl)benzyl)piperazine-1-carboxylate* (**S10**). White solid, yield 86.7%. ^1^H NMR (600 MHz, DMSO-*d*_6_) δ 7.19 (s, 1H), 7.18 (s, 1H), 7.12 (s, 1H), 4.34 (s, 2H), 4.03 (s, 4H), 3.68 (s, 2H), 3.01 (s, 4H), 1.41 (s, 9H). MS (ESI^+^): m/z calcd. for C_17_H_24_F_3_N_3_O_2_ [M+H]^+^ 360.19, found 360.20.

*Methyl 4-ethyl-3-ethynylbenzoate* (**S12**)*.* Yellow oil, yield 52.4%. ^1^H NMR (600 MHz, DMSO-*d*_6_) δ 7.94 (s, 1H), 7.89 (dd, *J* = 8.0, 1.8 Hz, 1H), 7.45 (d, *J* = 8.1 Hz, 1H), 4.48 (s, 1H), 3.84 (s, 3H), 2.81 (q, *J* = 7.5 Hz, 2H), 1.19 (t, *J* = 7.6 Hz, 3H); ^13^C NMR (151 MHz, DMSO-*d*_6_) δ 167.5, 153.0, 134.5, 131.3, 130.4, 129.2, 123.1, 86.8, 82.4, 53.9, 28.7, 16.0. MS (ESI^+^): m/z calcd. for C_12_H_12_O_2_ [M+H]^+^ 189.09, found 189.00.

*Methyl 4-ethyl-3-(pyrazolo[1,5-a]pyrimidin-6-ylethynyl)benzoate* (**S13**)*.* Yellow solid, yield 43.7%. ^1^H NMR (600 MHz, DMSO-*d*_6_) δ 9.56 (s, 1H), 8.69 (s, 1H), 8.32 (d, *J* = 2.2 Hz, 1H), 8.09 (d, *J* = 1.8 Hz, 1H), 7.91 (dd, *J* = 8.1, 1.8 Hz, 1H), 7.50 (d, *J* = 8.1 Hz, 1H), 6.82 (d, *J* = 1.3 Hz, 1H), 3.86 (s, 3H), 2.93 (q, *J* = 7.6 Hz, 2H), 1.26 (t, *J* = 7.5 Hz, 3H); ^13^C NMR (151 MHz, DMSO) δ 165.4, 151.0, 150.9, 146.6, 146.3, 138.3, 132.6, 129.8, 128.8, 127.7, 121.4, 104.7, 97.4, 90.0, 87.4, 66.4, 26.8, 14.9. MS (ESI^+^): m/z calcd. for C_18_H_15_N_3_O_2_ [M+H]^+^ 306.12, found 306.10.

*4-Ethyl-3-(pyrazolo[1,5-a]pyrimidin-6-ylethynyl)benzoic acid* (**S14**)*.* White solid, yield 84.5%. ^1^H NMR (600 MHz, DMSO-*d*_6_) δ 9.54 (d, *J* = 2.4 Hz, 1H), 8.69 (d, *J* = 2.1 Hz, 1H), 8.32 (d, *J* = 2.3 Hz, 1H), 8.07 (s, 1H), 7.87 (d, *J* = 9.2 Hz, 1H), 7.35 (d, *J* = 7.9 Hz, 1H), 6.82 (d, *J* = 2.6 Hz, 1H), 2.89 (q, *J* = 7.5 Hz, 2H), 1.26 (t, *J* = 7.6 Hz, 3H); ^13^C NMR (151 MHz, DMSO-*d*_6_) δ 151.5(2), 148.3, 147.0, 146.7, 138.4(2), 133.4, 130.6, 128.3, 120.7, 106.3, 98.4, 91.7, 87.0, 27.5, 15.2. MS (ESI^+^): m/z calcd. for C_17_H_13_N_3_O_2_ [M+H]^+^ 292.11, found 292.05.

**Cell Recovery, Culture, Passage, Treatment.** MDA-MB-231 cells were cultured in DMEM basal medium (Gibco, C11965500BT), while HCT-116 and NCI-H23 cells were maintained in RPMI-1640 medium (Gibco, C11875500BT). All culture media were supplemented with 10% fetal bovine serum (FBS, Gibco, 10099-141C) and 1% penicillin–streptomycin (Gibco, 15140122). Cells were incubated at 37 °C in a humidified atmosphere containing 5% CO₂. When the cells reached approximately 90% confluence and exhibited optimal morphology, they were passaged. Briefly, the culture medium was removed, cells were washed with PBS, and then digested with 1 mL of trypsin (Gibco, 25200072). Digestion was terminated by adding an equal volume of complete medium, followed by centrifugation at 1000 rpm for 5 min. The supernatant was discarded, and the cell pellet was resuspended in fresh medium and seeded into new culture vessels. For compound treatment, cells were collected, counted using a hemocytometer, and seeded at the desired density. All test compounds were dissolved in DMSO (Beyotime, ST038-100 mL) prior to use.

**Assessment of Medium Stability.** The compounds were diluted to 1.0 mM and incubated with DMEM medium supplemented with 10% fetal bovine serum (FBS). At designated time points, 20 μL aliquots were collected and analyzed by HPLC.

**Flow Cytometry Analysis of Intracellular ROS Levels.** MDA-MB-231 cells were trypsinized, resuspended in complete medium, and seeded into 6-well plates at a density of 1.5 × 10⁵ cells per well (2 mL medium per well). After overnight incubation to allow cell attachment, the medium was removed, and cells were gently washed with PBS. Cells were then treated with 500 nM **Ce6-TCO** for 8 h, followed by ultrasound irradiation under the indicated conditions. After irradiation, cells were incubated for an additional 2 h. To assess intracellular ROS levels, 1 mL of 10 μM DCFH-DA (Beyotime, S0033S) was added to each well and incubated at 37 °C for 20 min. Subsequently, cells were washed three times with PBS, digested with trypsin, and the digestion was quenched by adding an equal volume of complete medium. The cell suspension was collected into 1.5 mL tubes, centrifuged at 1000 rpm for 5 min, washed once with PBS, resuspended in PBS, and analyzed using a flow cytometer (Beckman Coulter, model A00-1-1102). Fluorescence intensity was quantified using FlowJo software (v10.8.1).

**Western Blot Analysis of Protein Degradation.** MDA-MB-231, HCT-116 and NCI-H23 cells were seeded in 6-well transparent culture plates (Corning, USA) at 1.5×10⁵ cells/well and cultured overnight at 37°C/5% CO₂. Attached cells were washed with ice-cold PBS and treated with compounds (**JQ1-Tz**, **NP-Tz**, **DR-Tz**) for 24h. After removal of medium and three PBS washes, **Ce6-TCO** was added and incubated for 8h prior to ultrasound treatment, followed by continued incubation for a total of 24h. After incubation, cells were washed with PBS, RIPA lysis buffer (Beyotime, P0013B) containing protease and phosphatase inhibitors (Beyotime, P1045) was added, and the mixture was incubated for 15 min. The cells were collected into a centrifuge tube, vortexed every 5 min for a total of three times, and then centrifuged at 12,000 rpm for 15 min at 4°C. The supernatant was collected, and protein quantification was performed using a BCA protein assay kit (Beyotime, P0010). The samples were mixed with loading buffer (Epizyme, LT101) and boiled. Proteins were separated by 10% sodium dodecyl sulfate-polyacrylamide gel electrophoresis (SDS-PAGE) and transferred to a 0.45 μm polyvinylidene fluoride (PVDF) membrane (Millipore, IPFL00010). The membrane was blocked with a protein-free rapid blocking buffer (Epizyme, PS108P) for 30 min. The anti-BRD4 primary antibody (Abcam, 128874), the anti-Visfatin primary antibody (Abcam, ab236874) , anti-DDR1 primary antibody (CST, 5583T) and anti-β-actin antibody (Proteintech, 66009-1-Ig) were added and incubated overnight at 4°C. After recovering the primary antibody, the membrane was washed with TBST. A secondary antibody (Proteintech, SA00001) was added and incubated at room temperature for 1 h. Protein bands were visualized using an ECL detection kit (Epizyme, SQ101L) and analyzed with ImageJ. Data were presented as mean ± SD, analyzed with GraphPad Prism 9.4.

**ROS Scavenging Assay.** MDA-MB-231 cells were seeded in 6-well clear flat-bottom plates (Corning, USA) at a density of 1.5 × 10⁵ cells per well and cultured overnight at 37 °C in a humidified atmosphere containing 5% CO₂. After cell attachment, the monolayers were gently washed once with ice-cold PBS and treated with **JQ1-Tz** for 24 h. The medium was then removed, and cells were washed three times with PBS, followed by incubation with **Ce6-TCO** for 6 h. Subsequently, 10 mM N-acetylcysteine (NAC) was added and incubated for 2 h prior to ultrasound irradiation. After irradiation, cells were further incubated until the total treatment duration reached 24 h. Protein expression levels were finally analyzed by Western blotting.

**Orthogonal *in vitro* experiment. JQ1-Tz** and **Ce6-TCO** were individually diluted to 500 nM in DMEM medium, mixed, and incubated on an orbital shaker at 80 rpm for 1 h. The mixture was then administered to cells. After 8 h of treatment, cells were exposed to ultrasound irradiation and further incubated until 24 h post-treatment initiation. Protein expression was assessed by Western blot analysis.

**Cell viability assay (cancer cells).** Triple-negative breast cancer cells MDA-MB-231 in logarithmic growth phase were seeded in 96-well plates at a density of 3×10³ cells per well. After overnight incubation at 37 °C with 5% CO₂, cells were treated with complete medium containing **JQ1-Tz** (500nM) for 24h. After removal of medium and three PBS washes, **Ce6-TCO** with concentration gradient was added and incubated for 8h prior to ultrasound treatment, followed by continued incubation for a total of 24h. Subsequently, the medium was replaced with fresh medium containing 10% CCK-8 reagent and further incubated for 45 minutes. The optical density (OD) at 450 nm was measured using a Cytation5 microplate reader (Bio-Tek, Vermont, USA). Finally, the half-maximal inhibitory concentration (IC₅₀) values were calculated using Prism GraphPad software with nonlinear regression and normalized dose-response fitting.

**Cell viability assay (normal cells).** Normal human mammary epithelial cells MCF 10A in logarithmic growth phase were seeded in 96-well plates at a density of 3×10³ cells per well. After overnight incubation at 37 °C with 5% CO₂, cells were treated with complete medium containing **JQ1-Tz** (500nM) for 24h. After removal of medium and three PBS washes, **Ce6-TCO** with concentration gradient was added and incubated for 24h. Subsequently, the medium was replaced with fresh medium containing 10% CCK-8 reagent and further incubated for 45 minutes. The optical density (OD) at 450 nm was measured using a Cytation5 microplate reader (Bio-Tek, Vermont, USA). Finally, the half-maximal inhibitory concentration (IC₅₀) values were calculated using Prism GraphPad software with nonlinear regression and normalized dose-response fitting.

**Immunofluorescence Analysis of Spatiotemporal location.** MDA-MB-231, HCT-116 and NCI-H23 cells were seeded in confocal dishes (MatTek, USA) at 1×10⁵ cells/dish and cultured overnight at 37°C/5% CO₂. Attached cells were treated with **JQ1-Tz**, **NP-Tz** and **DR-Tz** (500nM) for 24 h. After PBS (pH 7.4) washing, cells were incubated with **Ce6-TCO** for 4, 6, or 8 h, washed thrice, and stained with 5 μM DiO (Beyotime, C1038) for 15 min in the dark. Following three PBS washes, cells were fixed with 4% paraformaldehyde (Beyotime, P0099-100ml) for 15 min at RT, washed, and nuclei-stained with 1 μg/mL DAPI (Beyotime, P0131) for 15 min. After final washes, samples were mounted in anti-fade medium and imaged under a confocal microscope (Olympus, FV3000) using 63× oil immersion.

**Immunofluorescence Analysis of Protein Degradation.** MDA-MB-231 cells were seeded at 4×10^4^ cells/well into confocal dishes, **JQ1-Tz** (500 nM) was added and incubated for 24 h. After removal of medium and three PBS washes, **Ce6-TCO** was added and incubated for 8 h prior to ultrasound treatment, followed by continued incubation for a total of 24 h. cells were washed with PBS, fixed with 4% paraformaldehyde (Beyotime, P0099-100ml), blocked with 10% goat serum (BOSTER, AR1009), incubated with primary antibodies overnight at 4°C, followed by fluorescent secondary antibodies (HUABIO, HA1122). Nuclei were stained with DAPI. Images were obtained by confocal microscopy.

**Flow Cytometry Analysis of ROS level.** MDA-MB-231, HCT-116 and NCI-H23 cells were seeded in 6-well transparent culture plates (Corning, USA) at 1.5×10⁵ cells/well and cultured overnight at 37°C/5% CO₂. Attached cells were washed with ice-cold PBS and treated with compounds (**JQ1-Tz**, **NP-Tz**, **DR-Tz**) for 24 h. After removal of medium and three PBS washes, **Ce6-TCO** was added and incubated for 8 h prior to ultrasound treatment, followed by continued incubation for a total of 24 h. After incubation, Digest and harvest the cells, Add 1 mL of Foxp3 Fixation/Permeabilization working solution (Thermo Fisher, 00-5523) to each tube and vortex briefly. Incubate for 30-60 minutes at 2-8°C or room temperature protected from light. Add 2 mL of 1X Permeabilization Buffer (Thermo Fisher, 88-8824-00) to each tube, then centrifuge at 500 × g for 5 min at room temperature. Repeat this wash step twice. Resuspend the pellet in the remaining volume of 1X Permeabilization Buffer. Directly add 2 μL of 2% goat serum blocking solution to the cells and incubate for 15 min at room temperature. Without washing, add fluorochrome-conjugated antibodies targeting intracellular antigens and incubate for ≥ 30 min at room temperature in the dark. Add 2 mL of 1× Permeabilization Buffer to each tube and centrifuge at 500 × g for 5 min. Discard the supernatant. Repeat the washing procedure (adding 2 mL buffer + centrifugation) twice. Resuspend stained cells in an appropriate volume of Flow Cytometry Staining Buffer (Thermo Fisher, 00-4222) and analyze by flow cytometry.

**Proteome assay.**MDA-MB-231 cells in good condition were digested and 9 × 10^7^ cells were seeded in nine cell-culture dishes for 24 h. Then, **JQ1-Tz**+**Ce6-TCO**+**US**-treated and **JQ1-Tz**+**Ce6-TCO-**treated control groups were incubated with cells, respectively. Repeat 3 groups for each compound. After incubation, cells were washed with PBS and then lysed by RIPA lysis buffer on the ice for 30 min. Total cell protein was obtained from the supernatant collected by centrifuging the cell lysate (12000 g, 4 °C). Subsequently, the cell lysate (100-200 µg) was sent to OEBiotech company for proteomic assay. Briefly, cellular samples were processed to extract total proteins, a portion of which was utilized for protein concentration determination and SDS-PAGE analysis. Another portion was subjected to trypsin digestion and labeling, followed by equal mixing of the labeled samples for chromatographic separation. Subsequently, the samples were subjected to LC-MS/MS analysis, and the acquired data were subjected to comprehensive data analysis.

**Bioinformatical analysis**. Following protein identification and quantification using Proteome Discovery v1.4 software, we obtained the expression profiles of proteins in each sample. The first and foremost step involved checking data quality through Pearson correlation analysis. In our study, each experimental group was replicated three times, and data were processed by filtering out outliers using Mean Absolute Differences (MAD) and imputing missing values through a random forest-based algorithm. After consolidating the data, we employed linear models (limma v3.52.4 R package) to analyze significantly differentially expressed proteins between the two groups (The above experimental procedures were conducted by OEBiotech company). Volcano plots, expression pattern clustering heatmaps, Venn analysis, and Gene set enrichment analysis (GSEA) were employed for differential comparison group data. To gain a deeper understanding of differentially expressed proteins in the target KEGG pathway, we focused on the MAPK signaling pathway and integrated it with proteins of interest. Lastly, we conducted enrichment analysis for the proteins of interest using R packages cluster Profiler v4.4.4 and org.Hs.eg.db v3.15.0.

***In Vivo* Therapeutic Efficacy.** All the animal protocols were assessed and approved by the Committee on Ethics of Medicine, Navy Medical University (SMMU82030105). BALB/C nude female mice (certificate SCXK-2021-0013, weighing 17−20 g) were obtained from Changzhou Cavens Experimental Animal Co., Ltd. MDA MB 231 cells (6 × 10⁶ cells per mice) were subcutaneously injected into the right flank. When the tumor volume reached approximately 120 ± 10 mm³, the mice were randomly divided into four groups (n = 6 per group): (1) **JQ1-Tz** + **Ce6-TCO**; (2) **JQ1-Tz + Ce6-TCO** + Ultrasound; (3) Ultrasound control; and (4) PBS control. Treatment groups received intraperitoneal injections of **JQ1-Tz** (10 mg/kg in PBS) and an equivalent dose of **Ce6-TCO** every other day. All drugs were dissolved in PBS and 10% TWEEN 80 was added to enhance solubility. For the ultrasound treatment group, tumor sites were subjected to ultrasound irradiation (1 MHz, 1.0 W/cm², 50% duty cycle, 2 min) 8 h after **Ce6-TCO** administration. The ultrasound control group received the same ultrasound exposure every 48 h, while control mice were administered an equal volume of PBS (100 μL per 20 g body weight). Tumor length (A) and width (B) were measured every two days, and tumor volumes were calculated using the formula *V = AB²/2*. Body weight was recorded simultaneously. On day 21, the mice were euthanized, and tumors were excised, photographed, and weighed. Major organs (heart, liver, spleen, lungs, and kidneys) were collected, weighed, and fixed in 4% paraformaldehyde (PFA) for histological analysis. Data are presented as mean ± SD and analyzed using one-way ANOVA followed by Tukey’s post hoc test in GraphPad Prism 9.0. Statistical significance was defined as *P* < 0.05.

**Hematoxylin-Eosin Staining and Histology**. After 14 days post-treatment, the heart, liver, spleen, lung, kidney, and tumors of all treatment groups were dissected and fixed with 4%paraformaldehyde. The tissue of organs was sliced and stained by Bios Biological Company.

**Statistical analysis.** Statistical Analysis. All data are presented as mean ± standard deviation (SD), and each experiment was repeated at least three times. Statistical significance was evaluated using Student’s t-test or one-way analysis of variance (ANOVA), as appropriate. A P value of < 0.05 was considered statistically significant. All analyses were performed using GraphPad Prism software.

**The spectra of the synthesized compounds**

Compound **3**


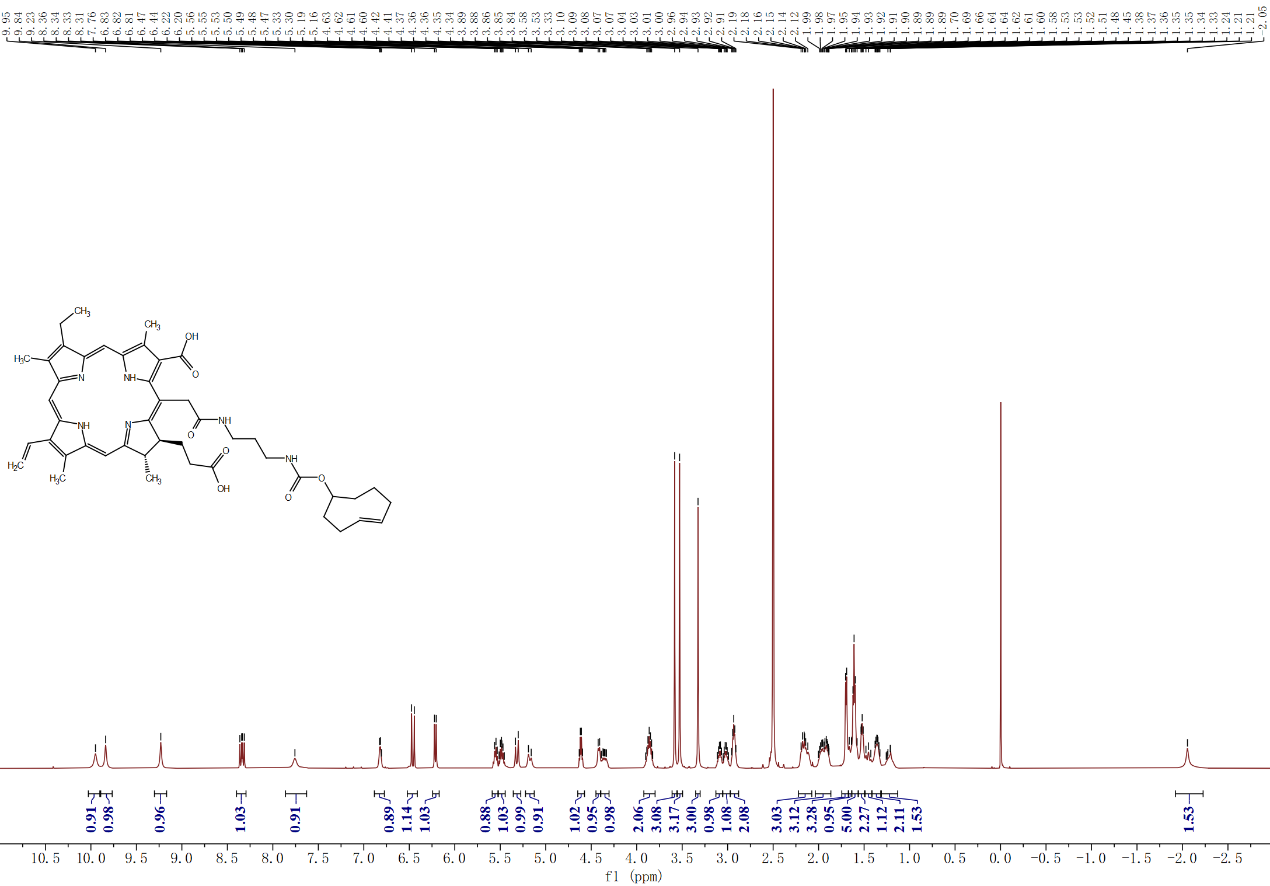


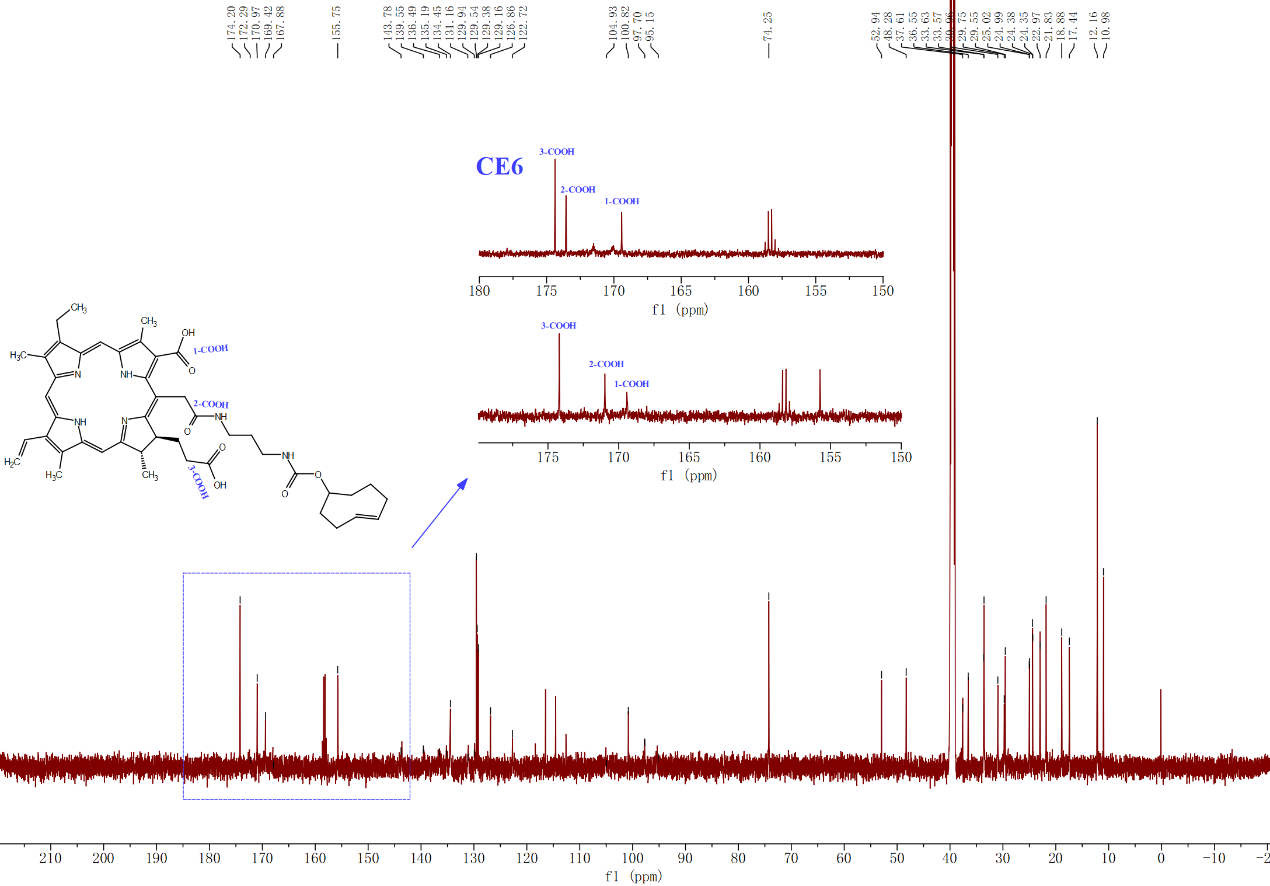

Compound **6**

**
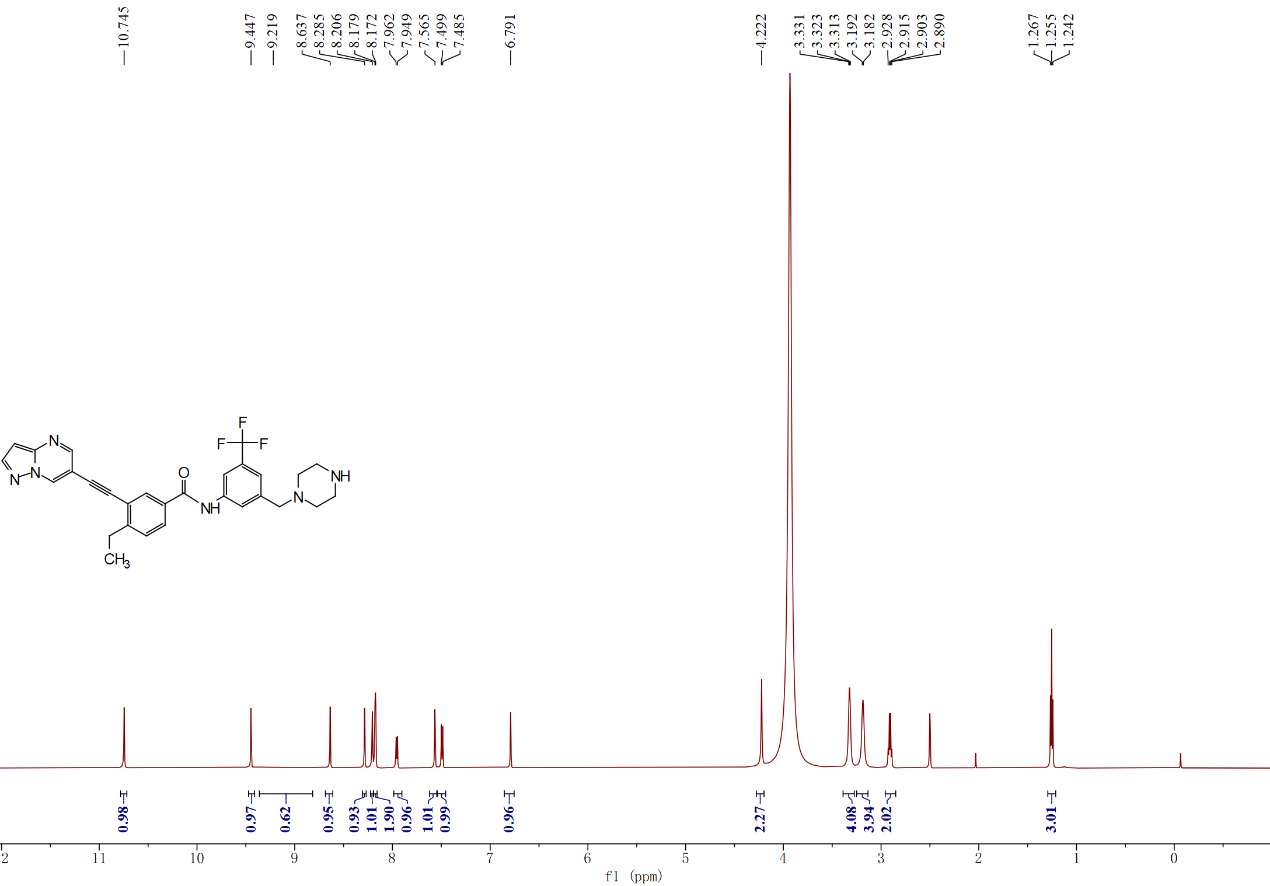
**

**
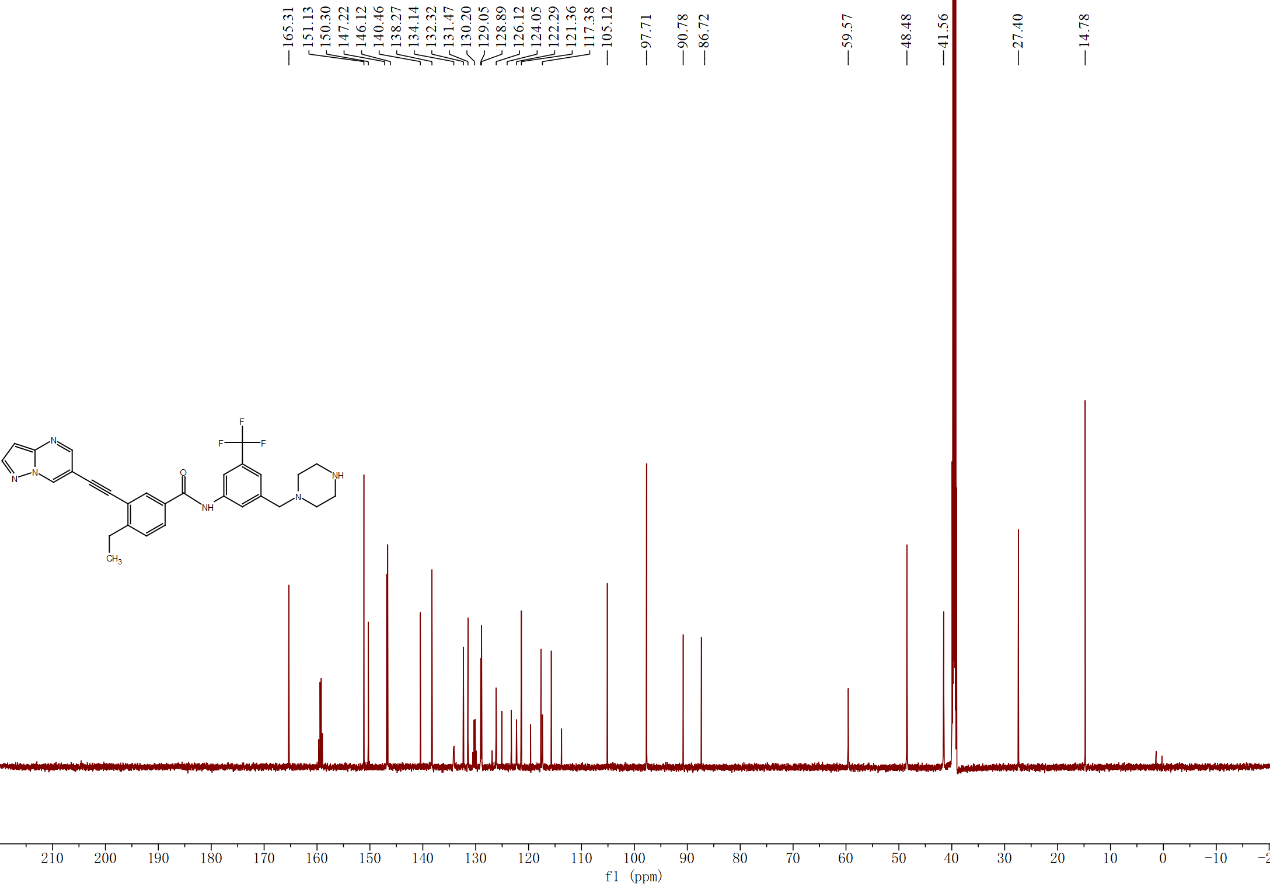
**

Compound **9**

**
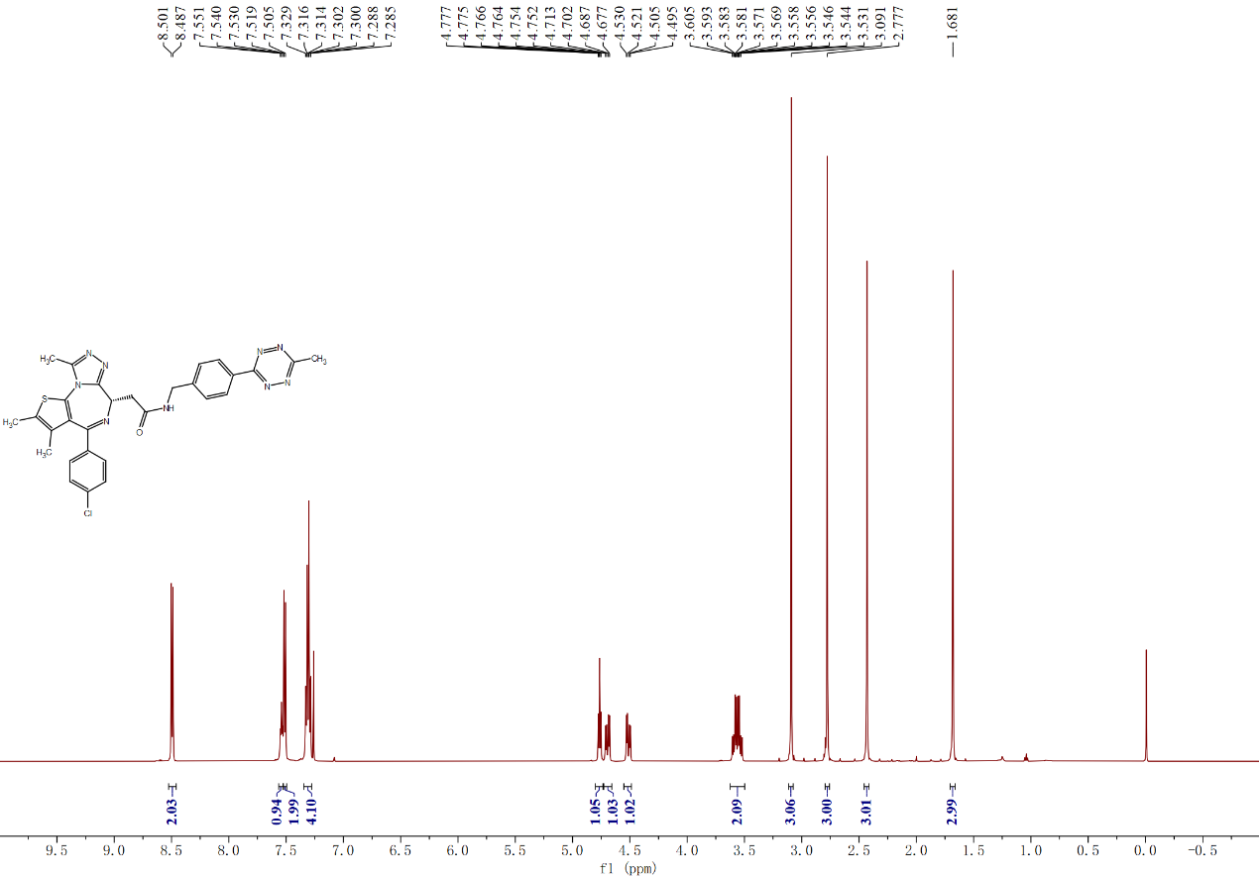
**

**
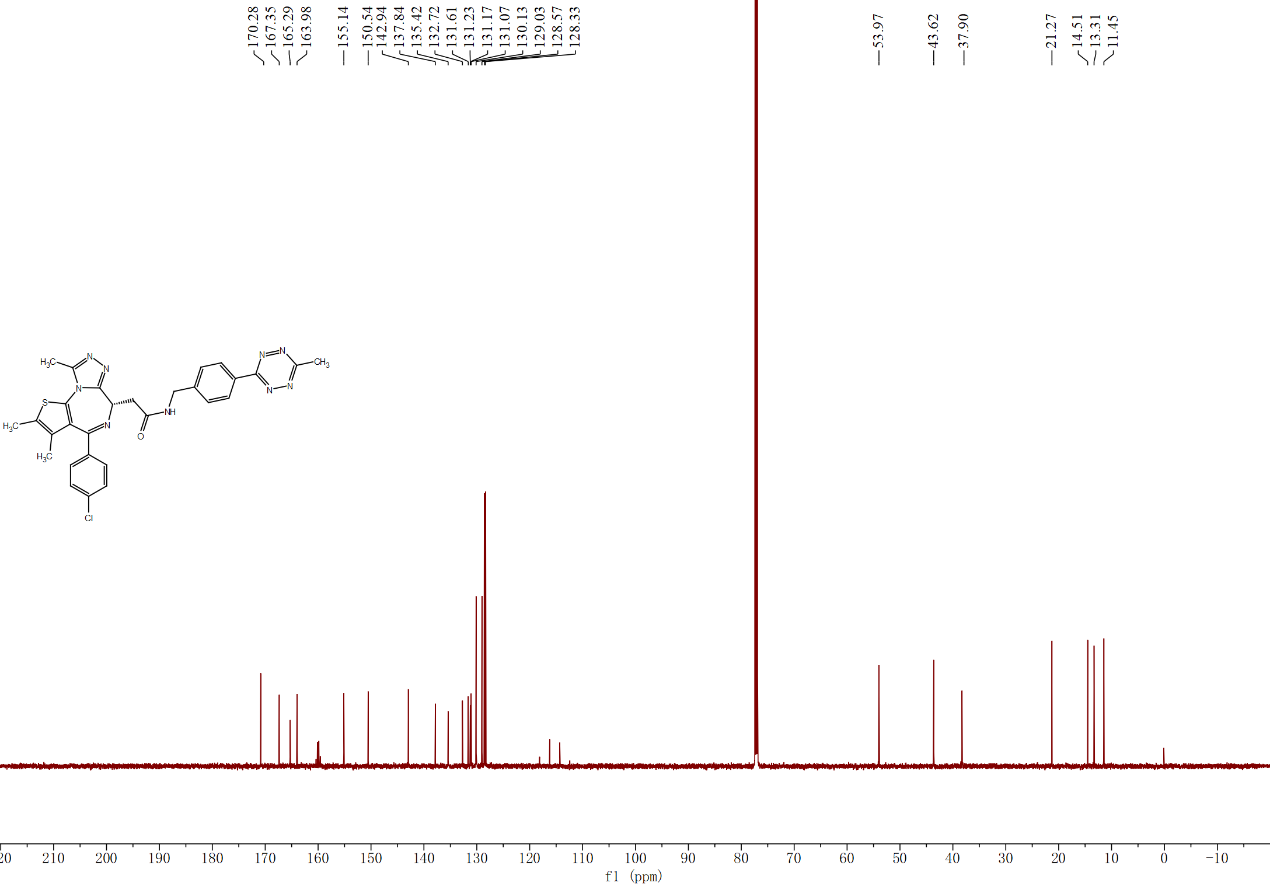
**

Compound **10**

**
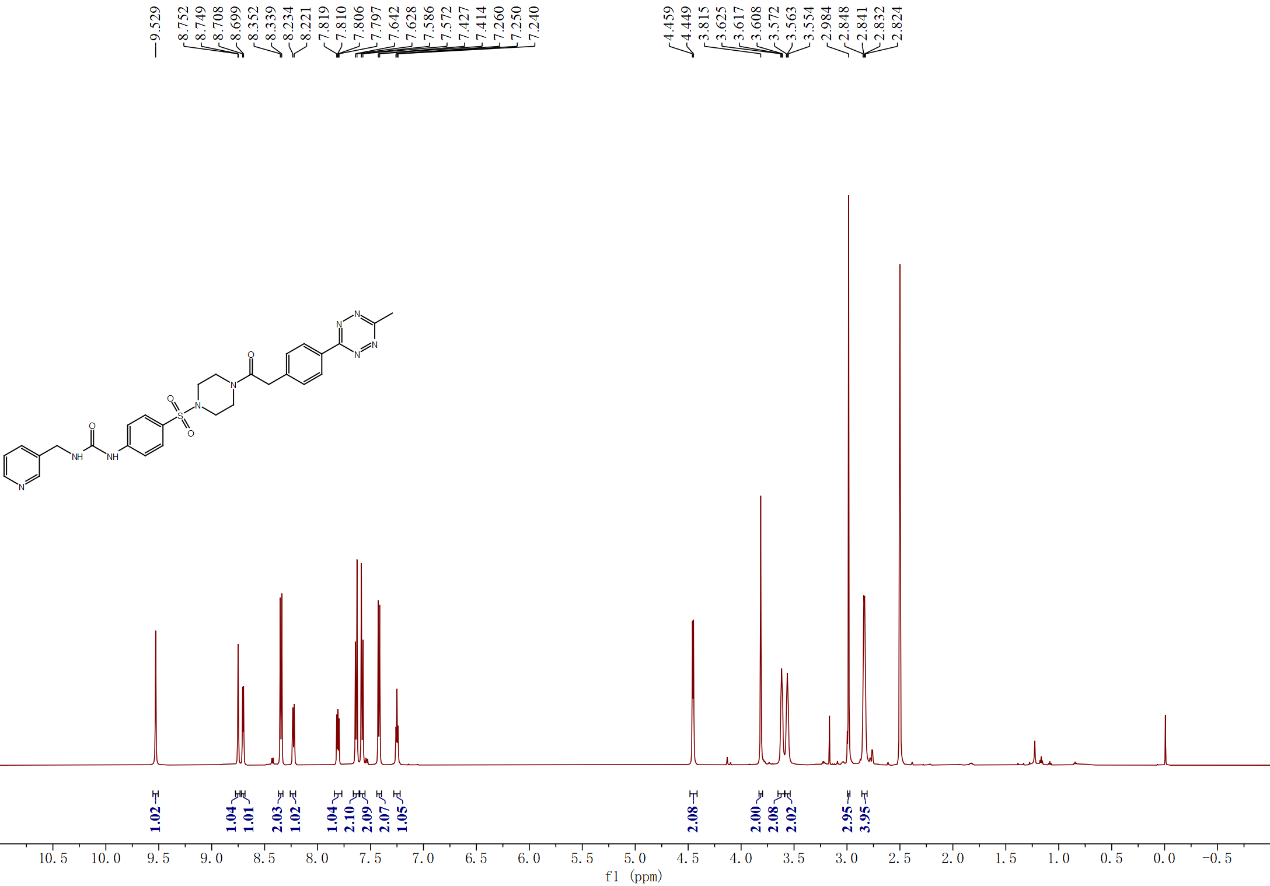
**

**
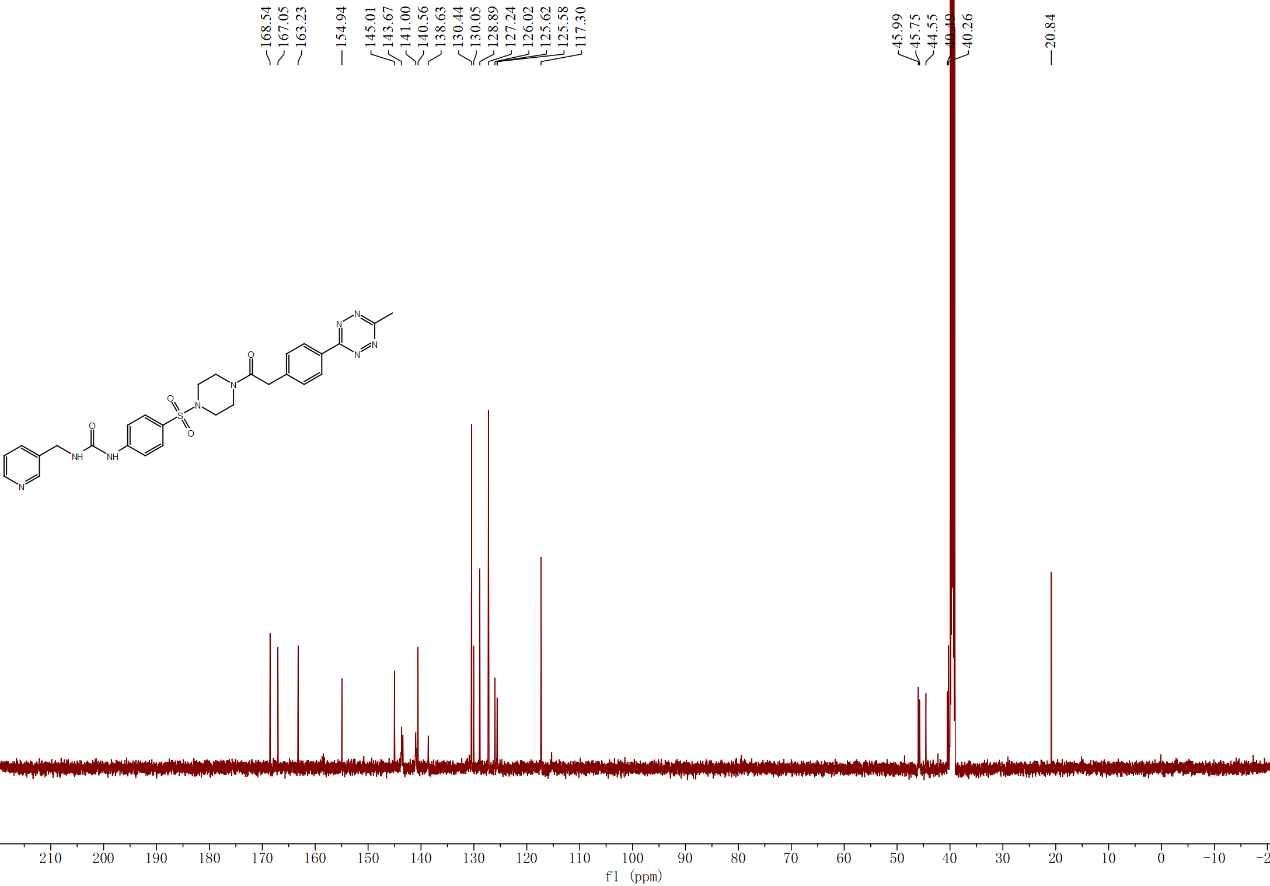
**

Compound **11**

**
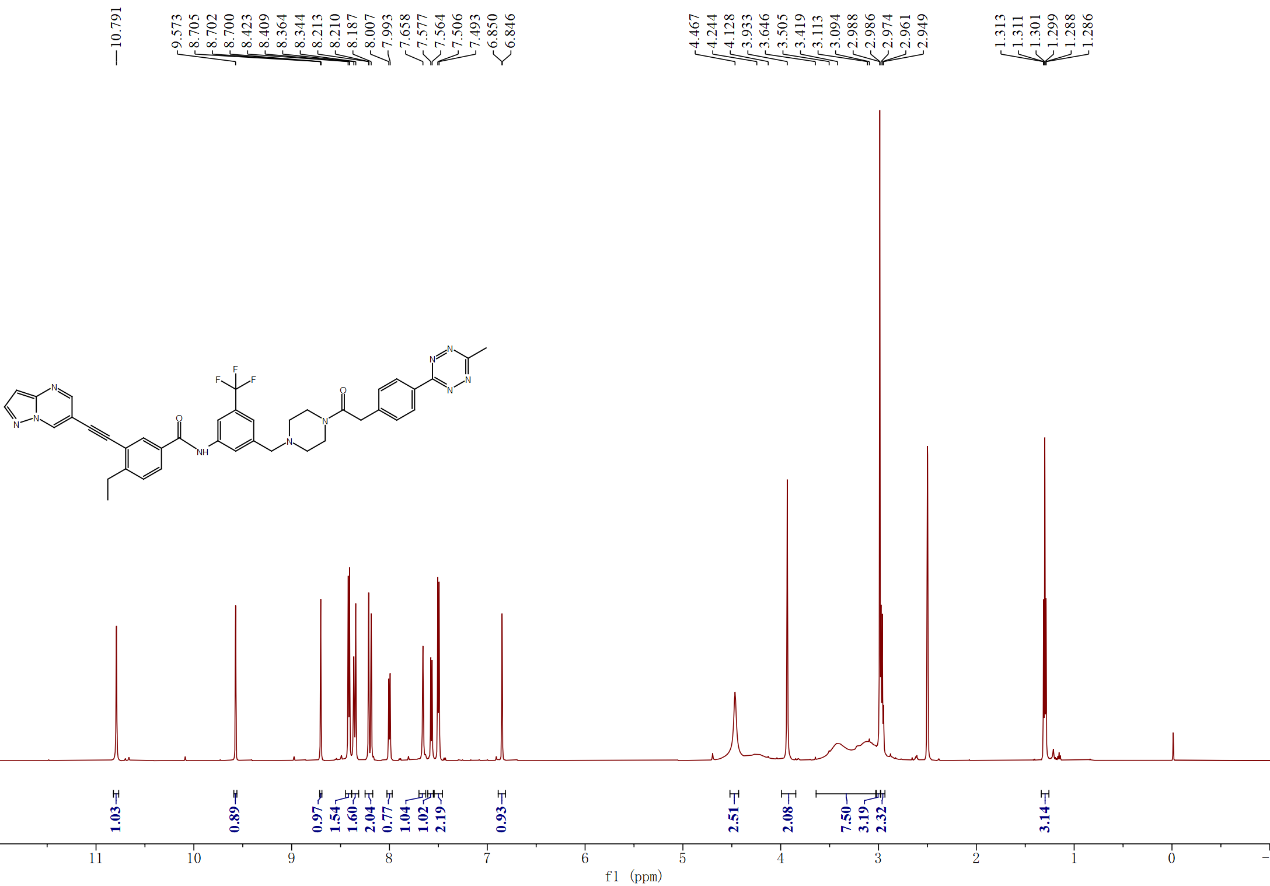
**

**
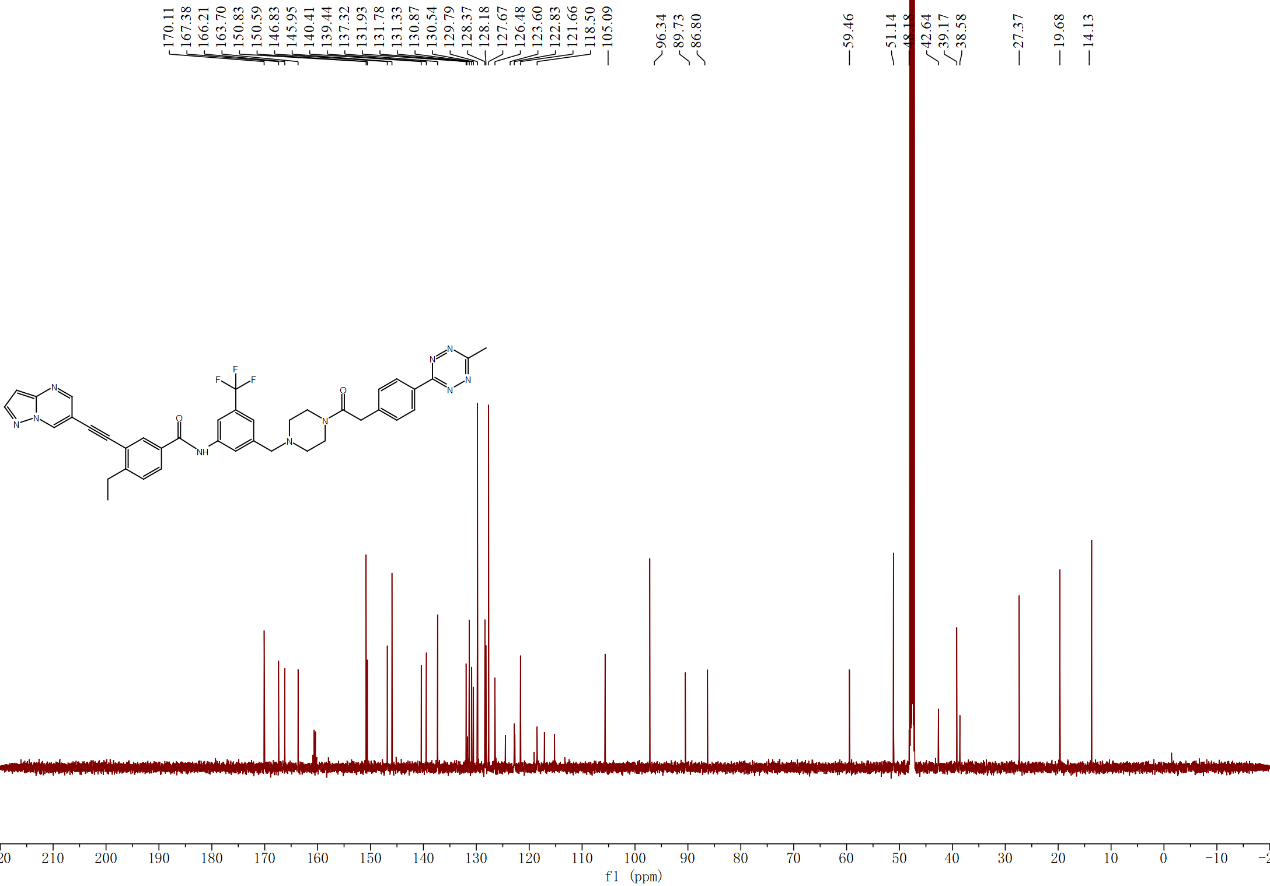
**

Compound **S3**

**
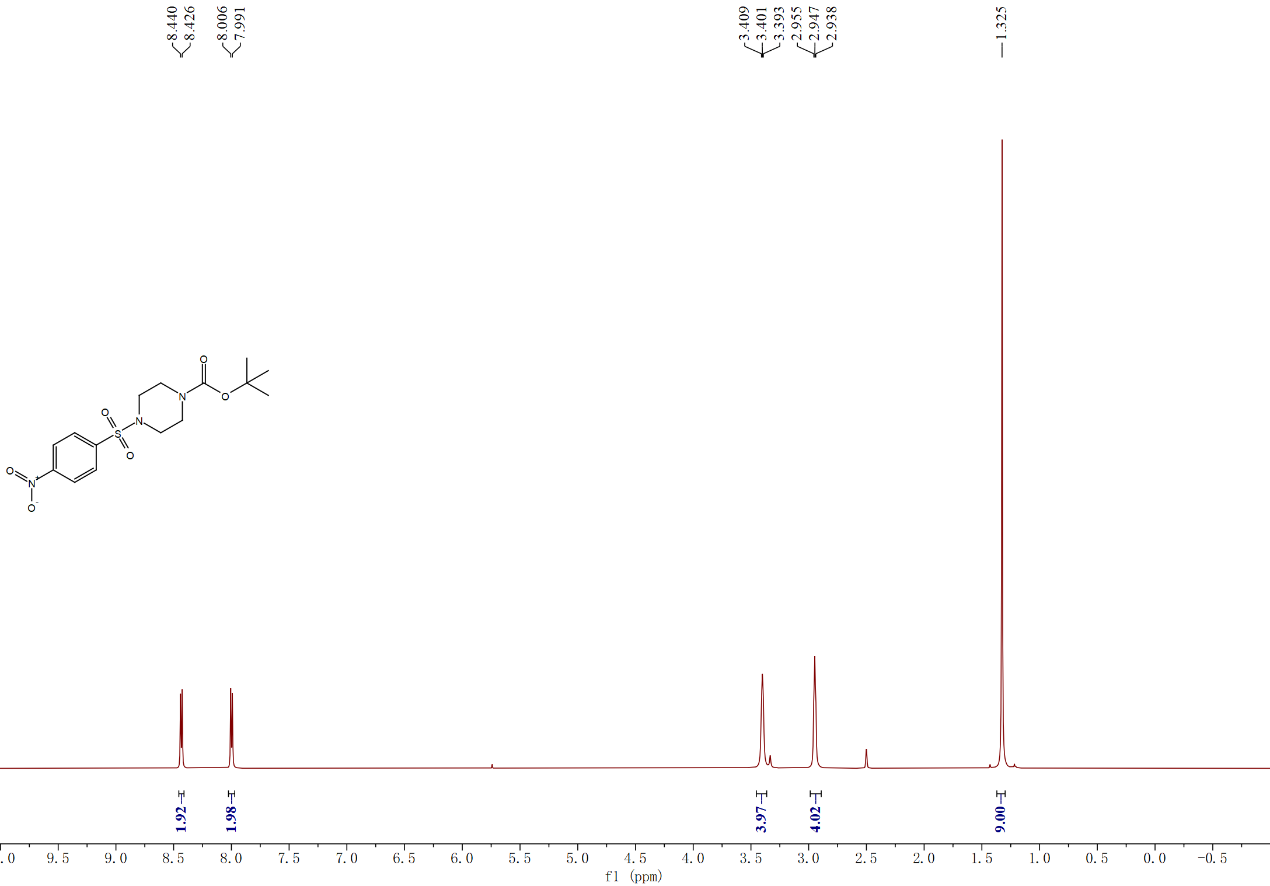
**

**
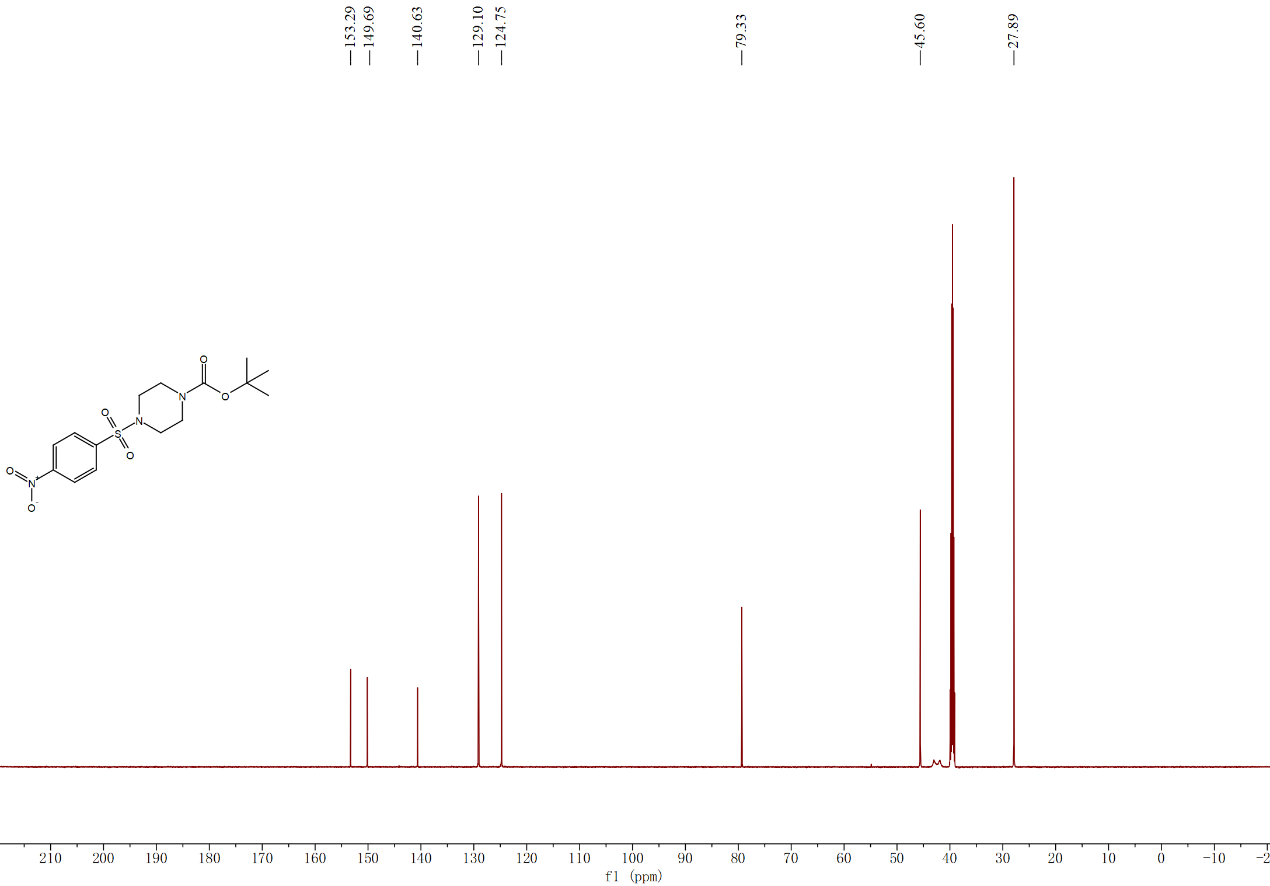
**

Compound **S4**

**
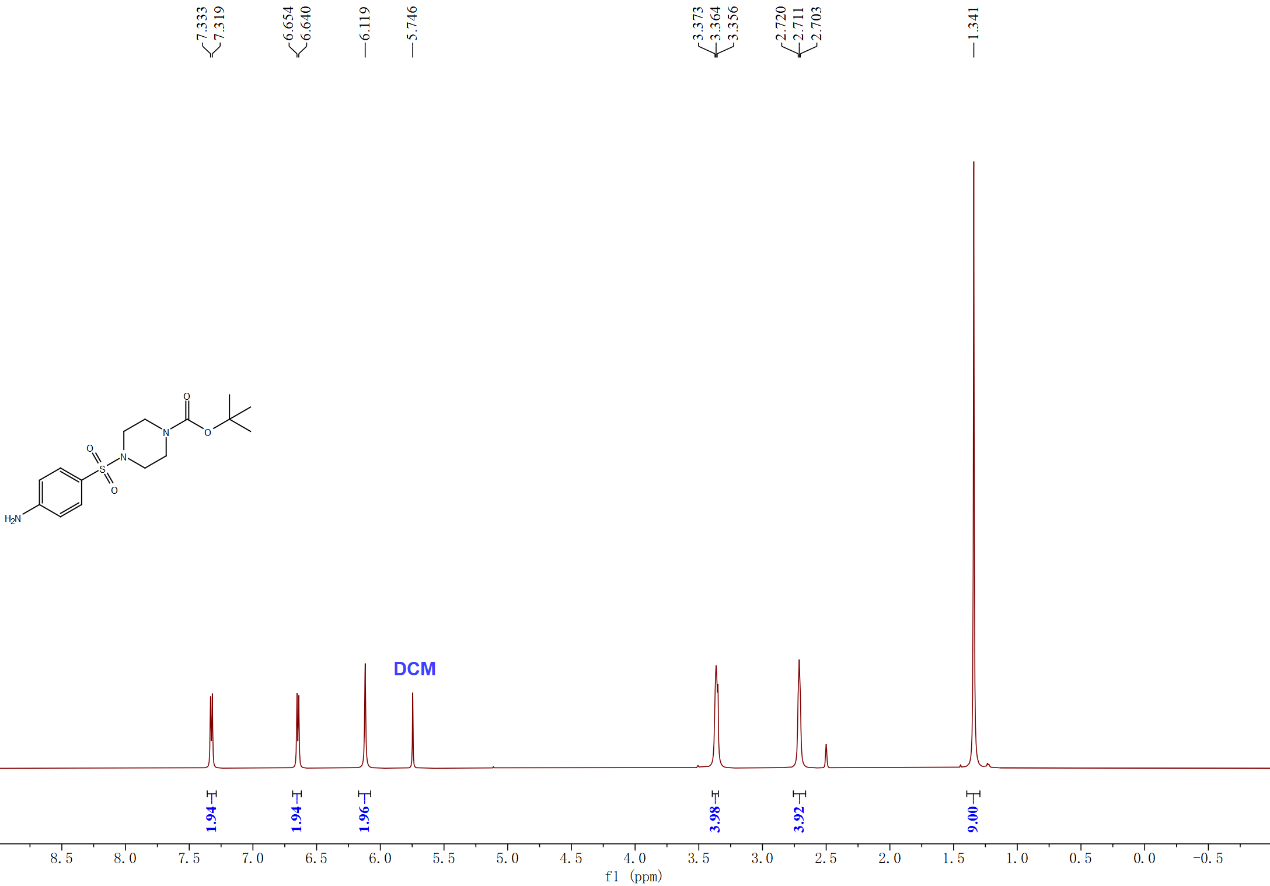
**

**
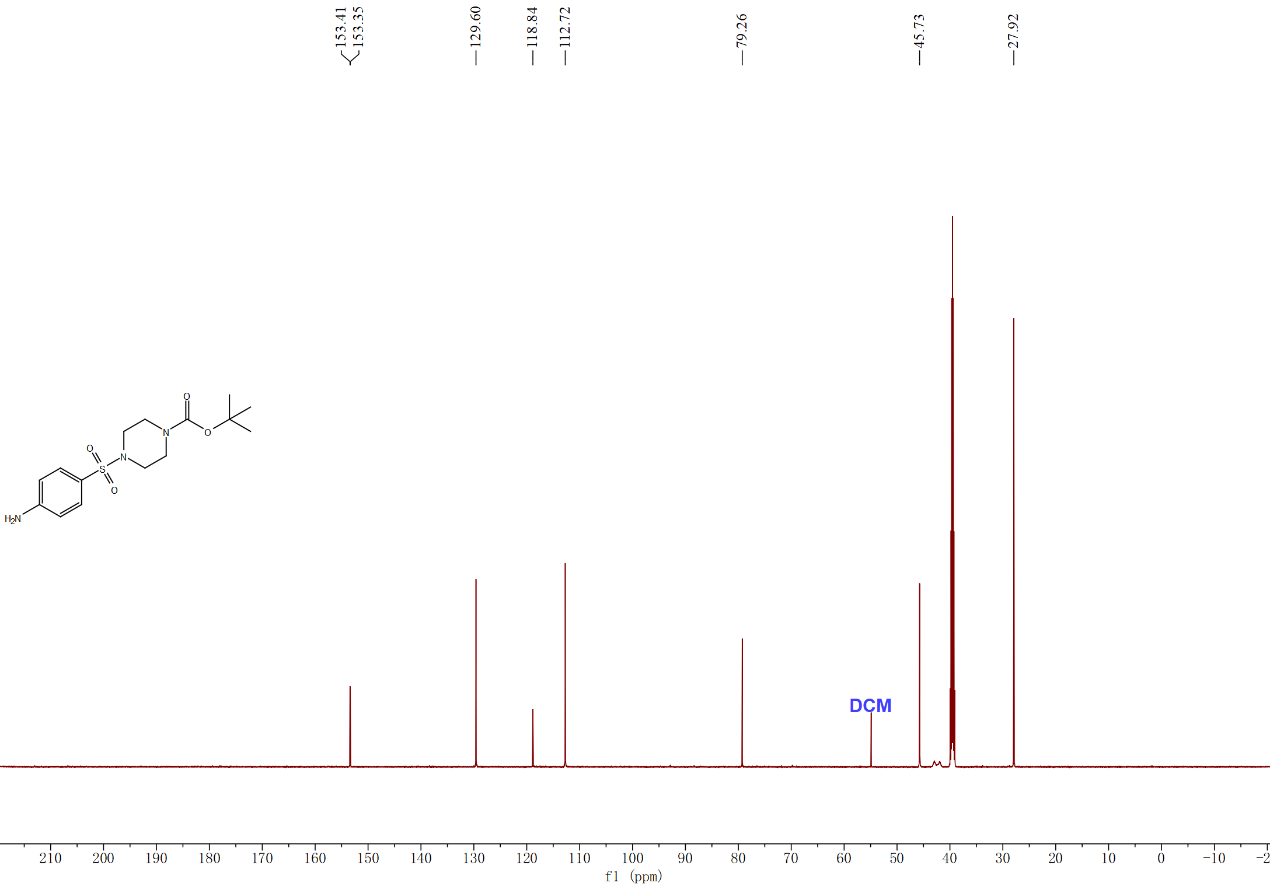
**

Compound **S5**

**
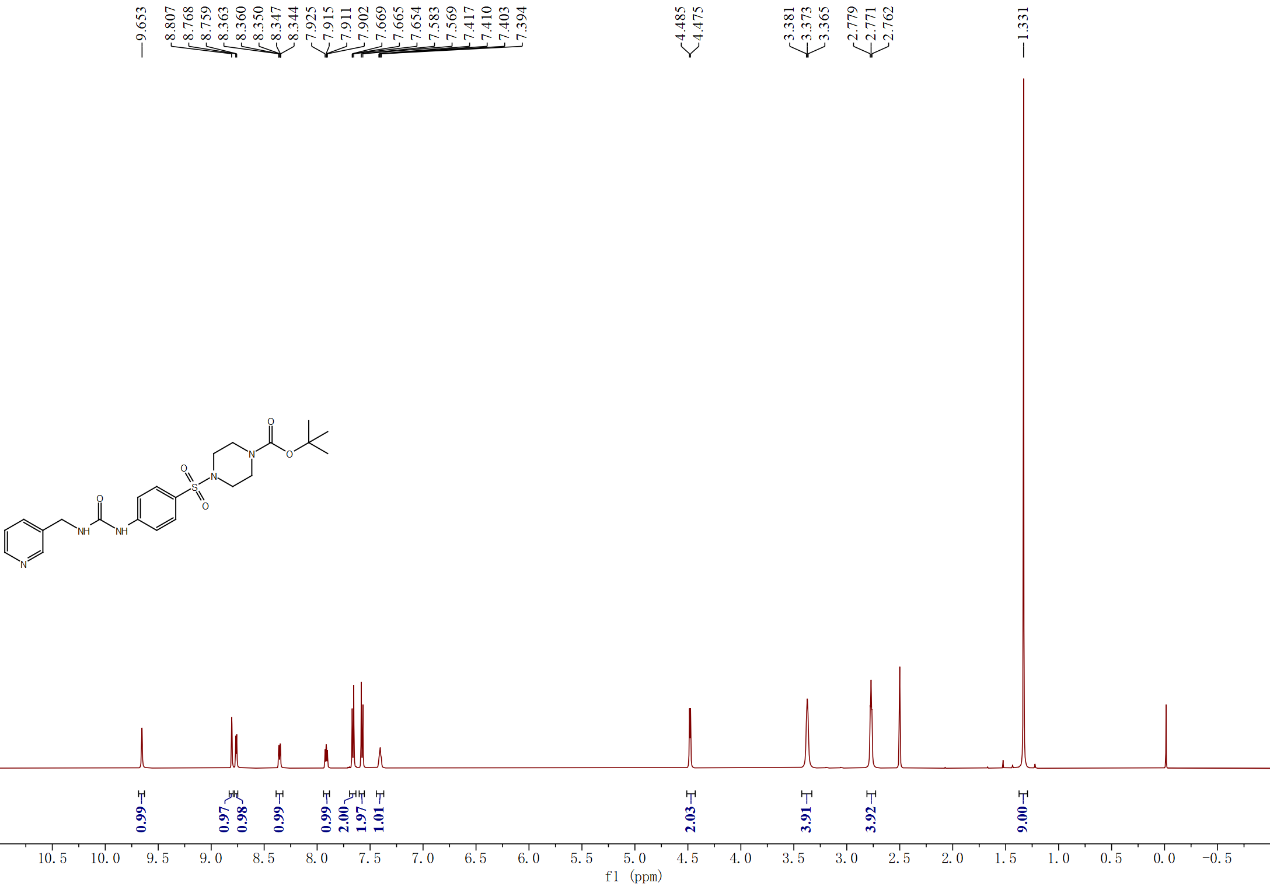
**

**
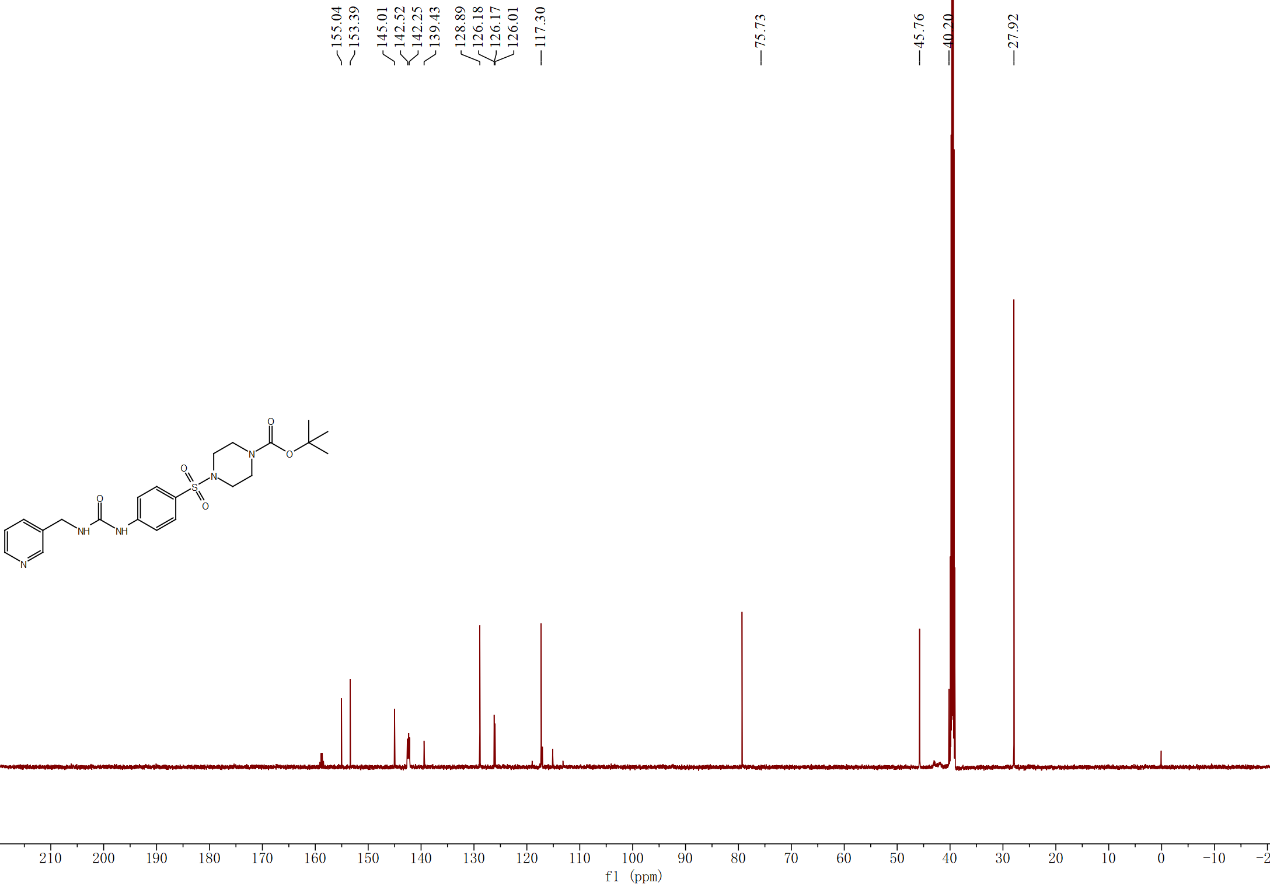
**

Compound **S7**

**
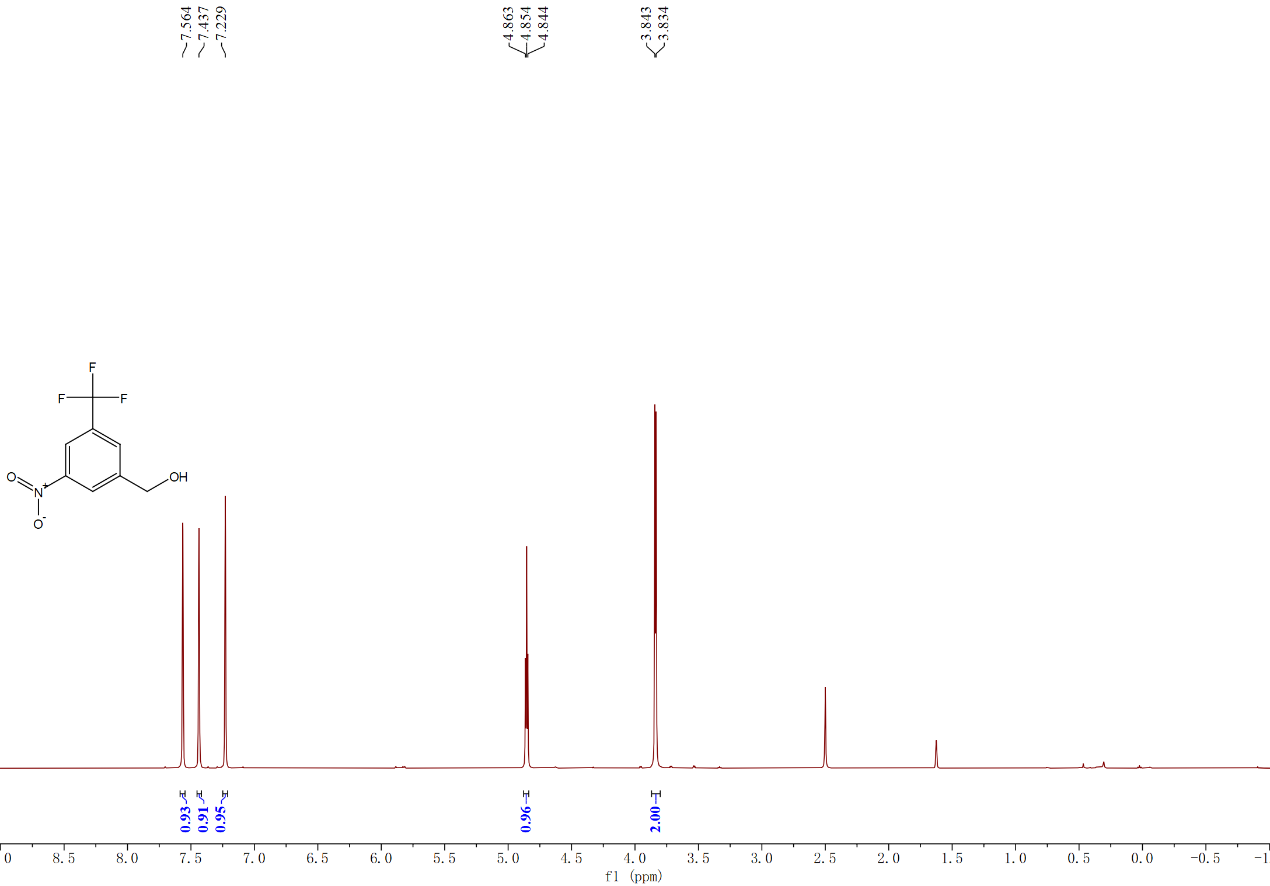
**

**
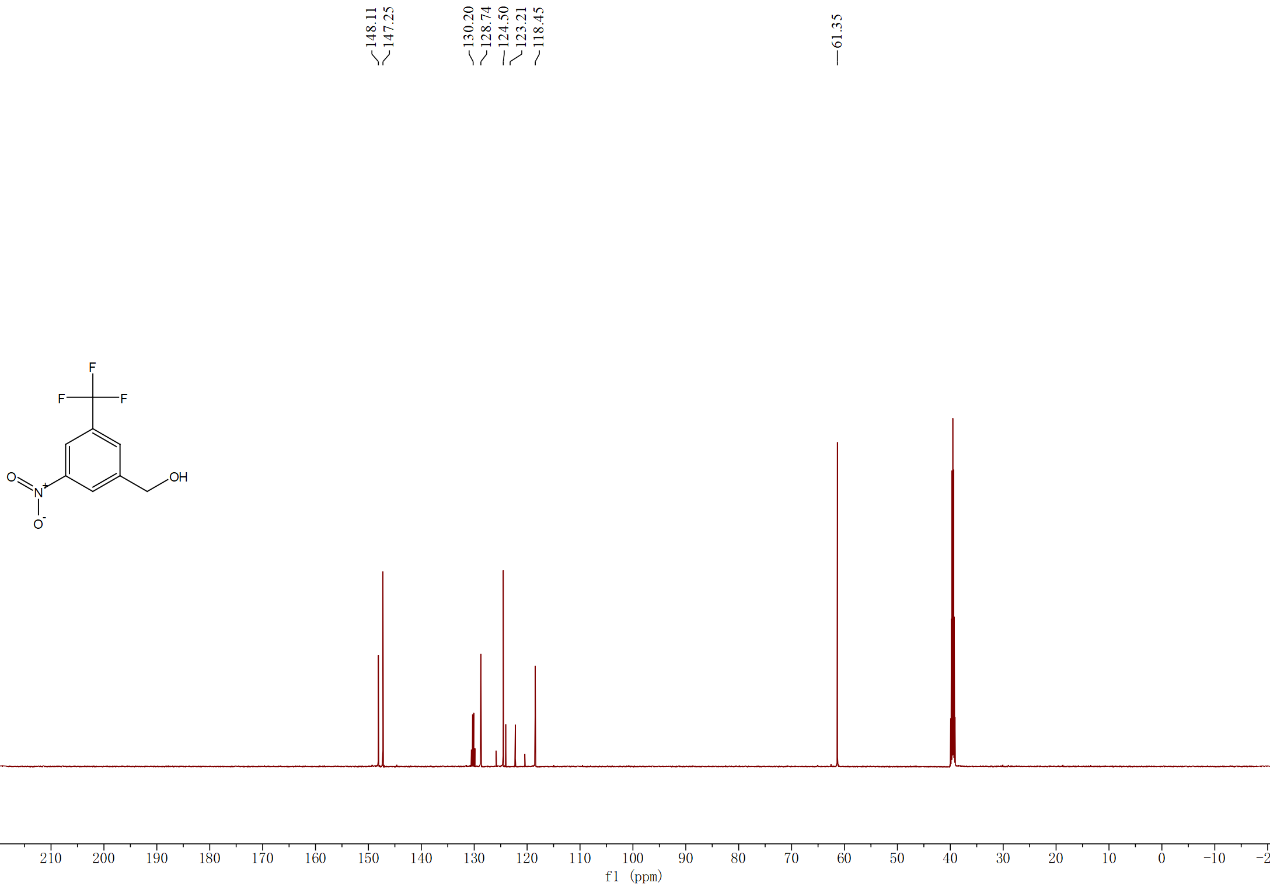
**

Compound **S8**

**
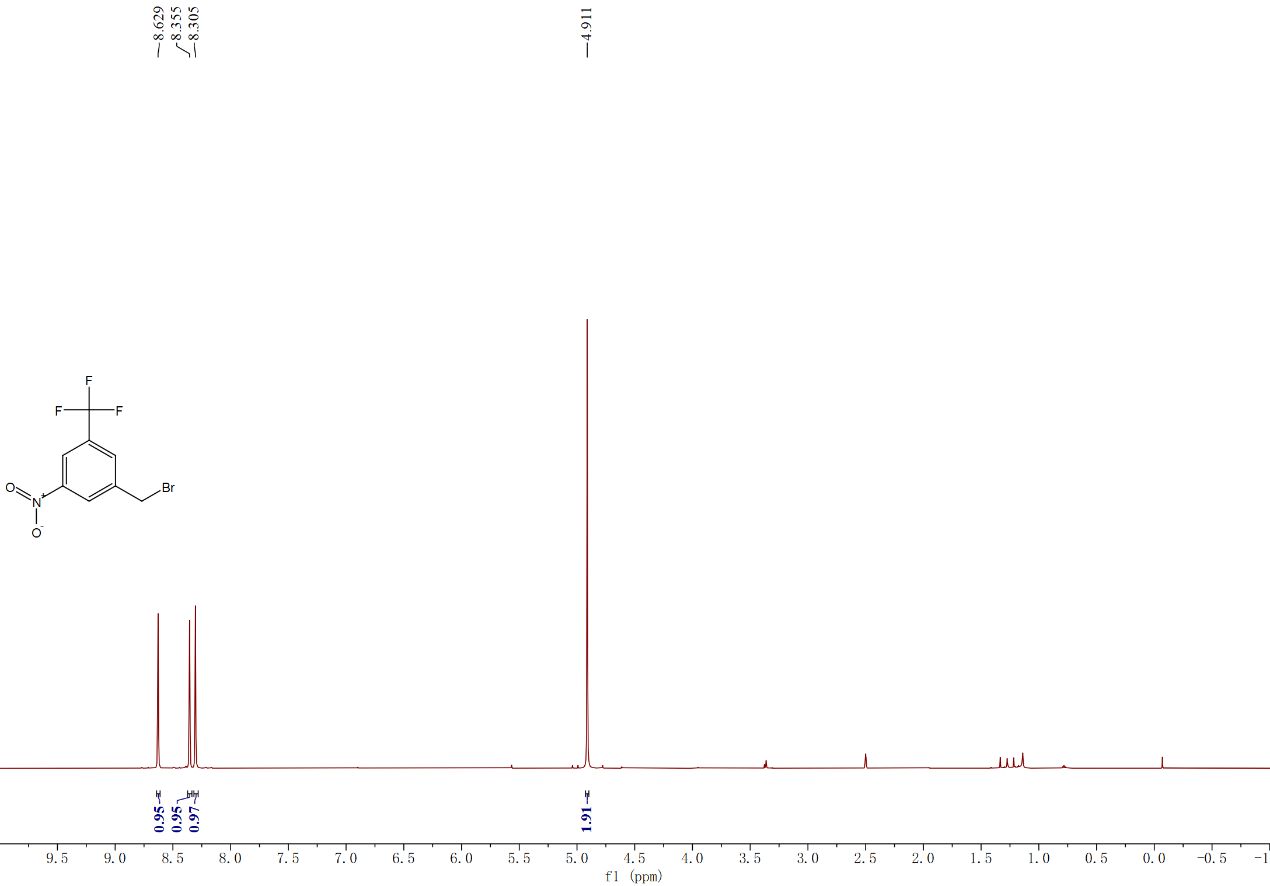
**

**
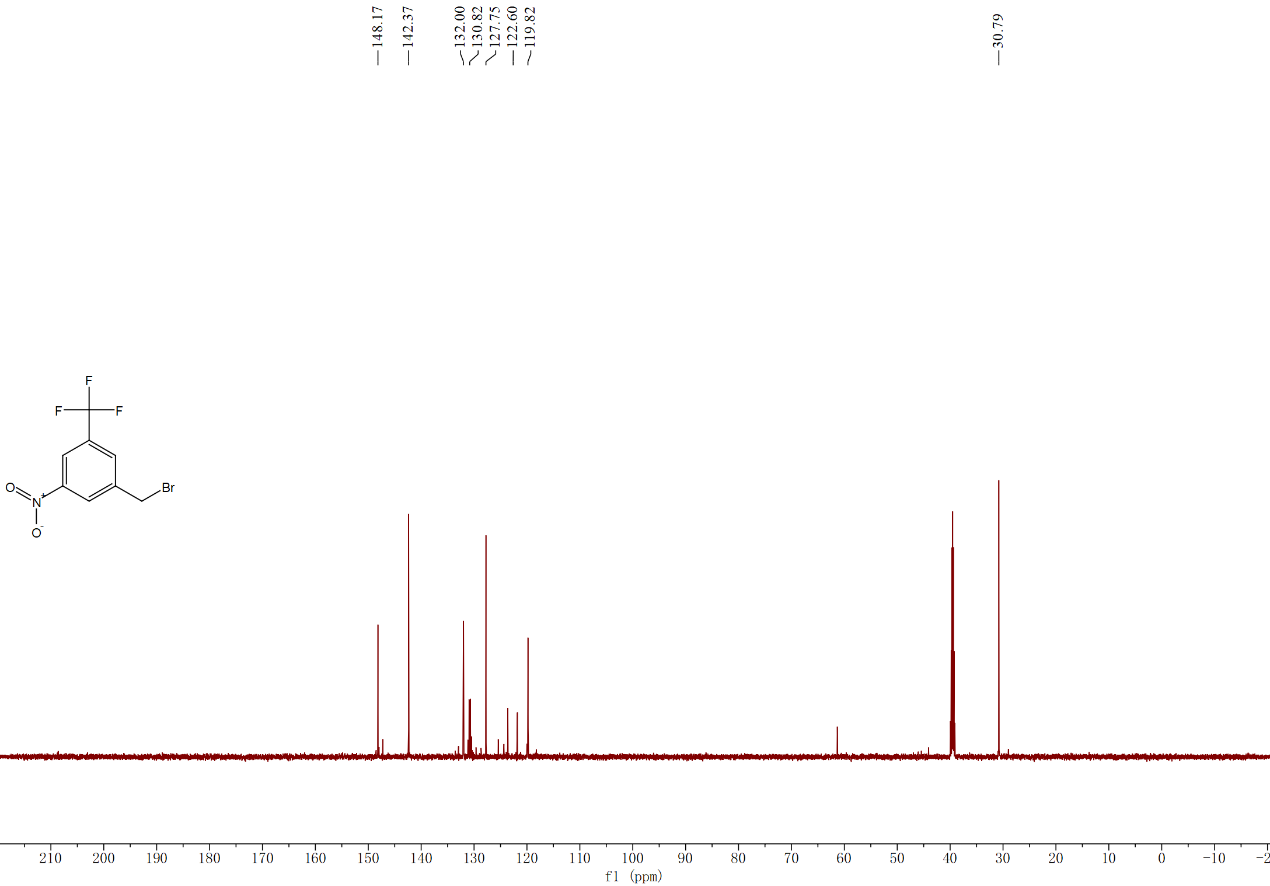
**

Compound **S9**

**
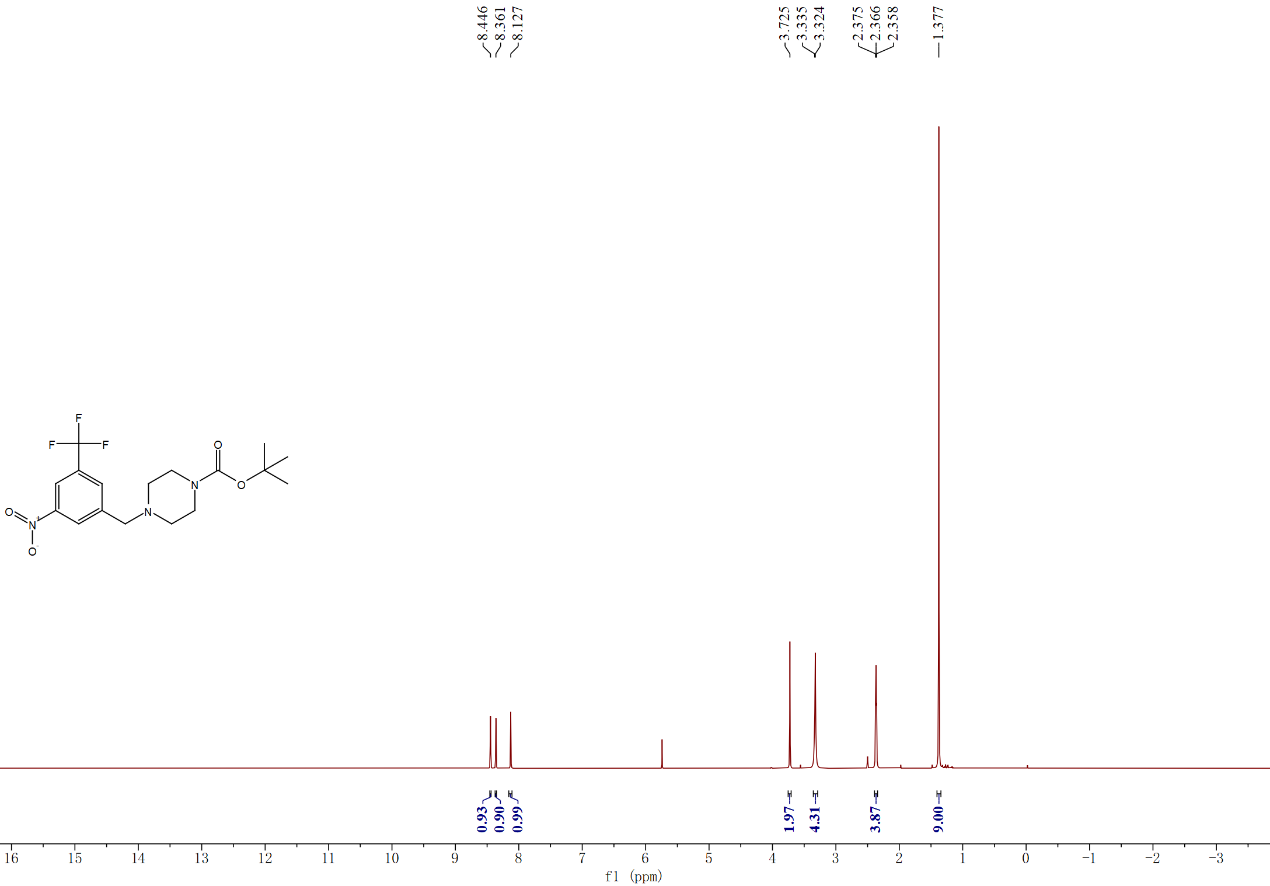
**

**
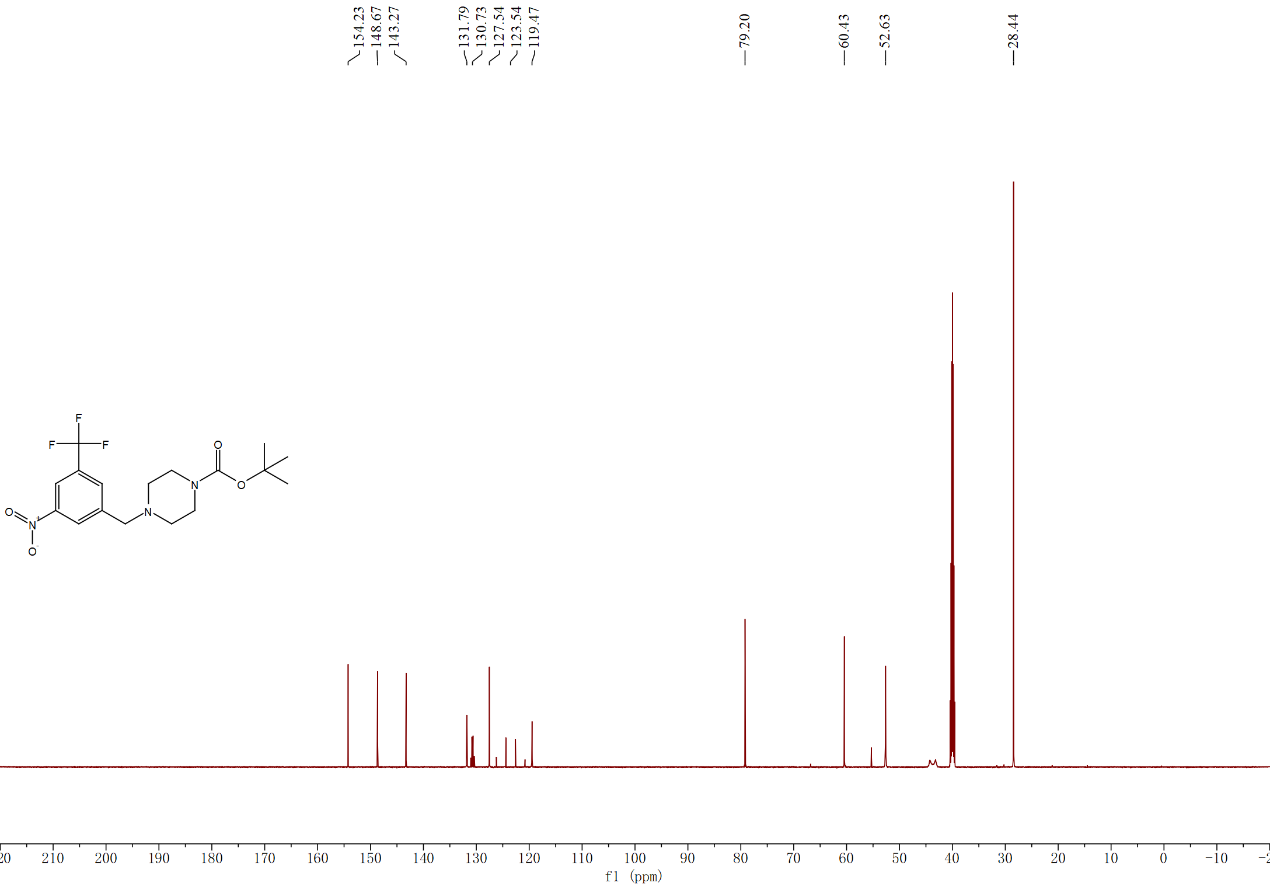
**

Compound **S10**

**
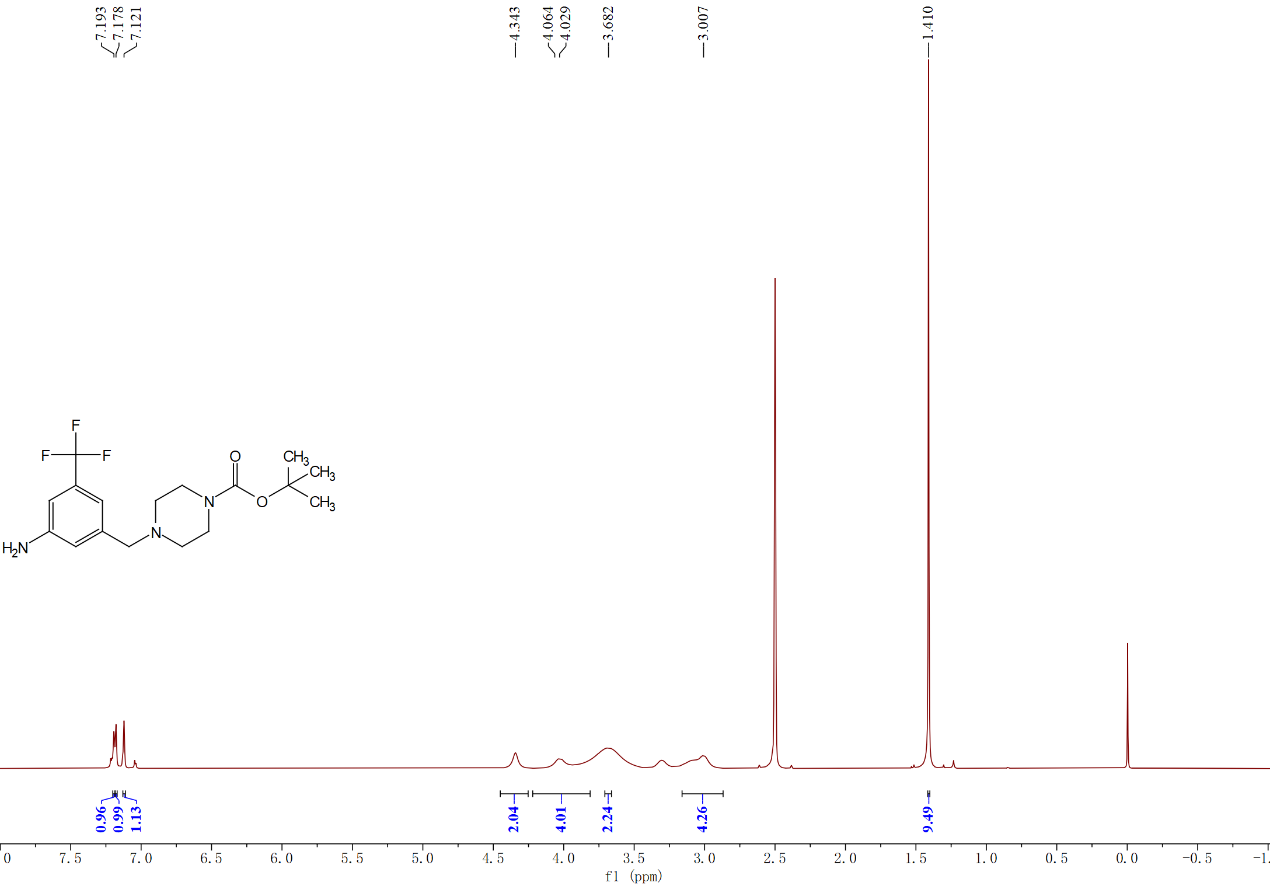
**

Compound **S12**

**
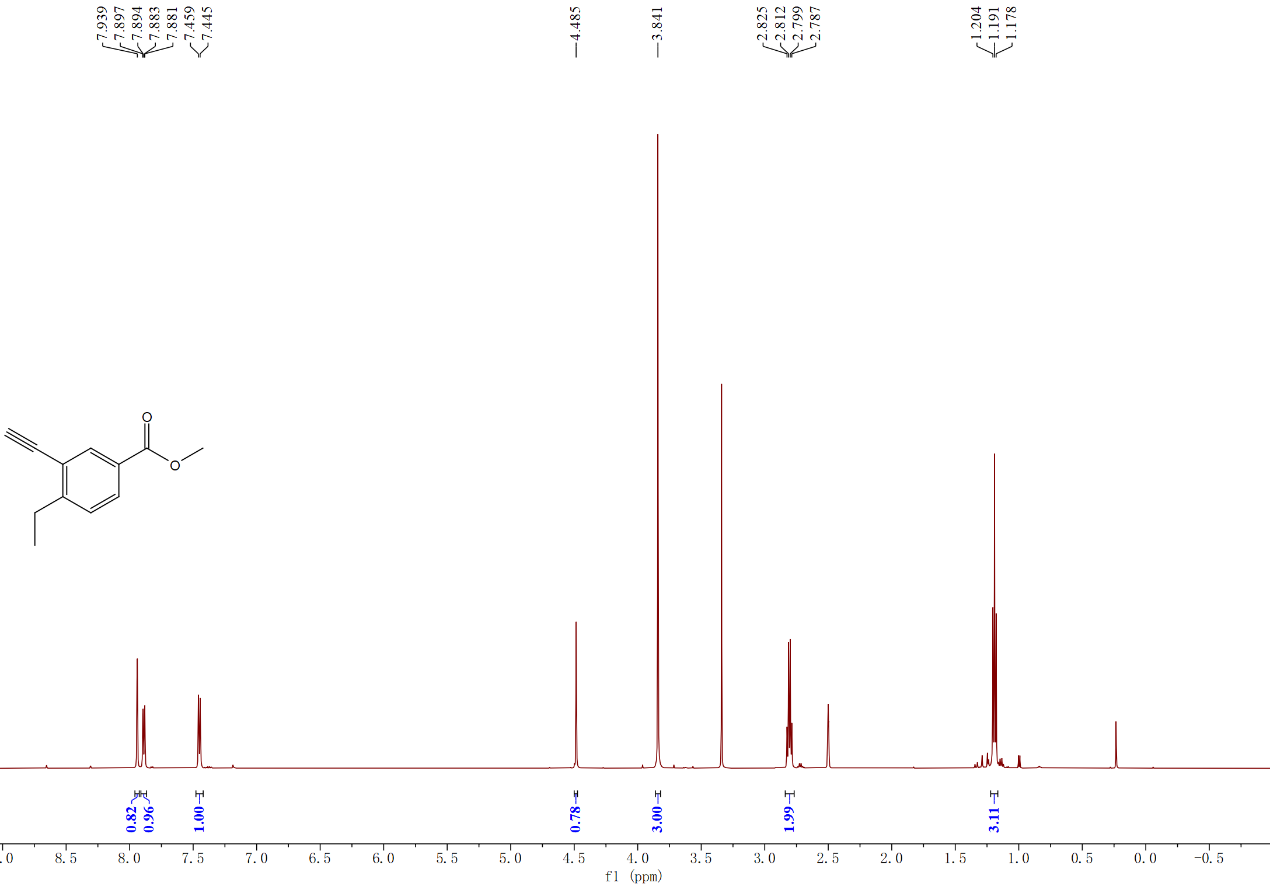
**

**
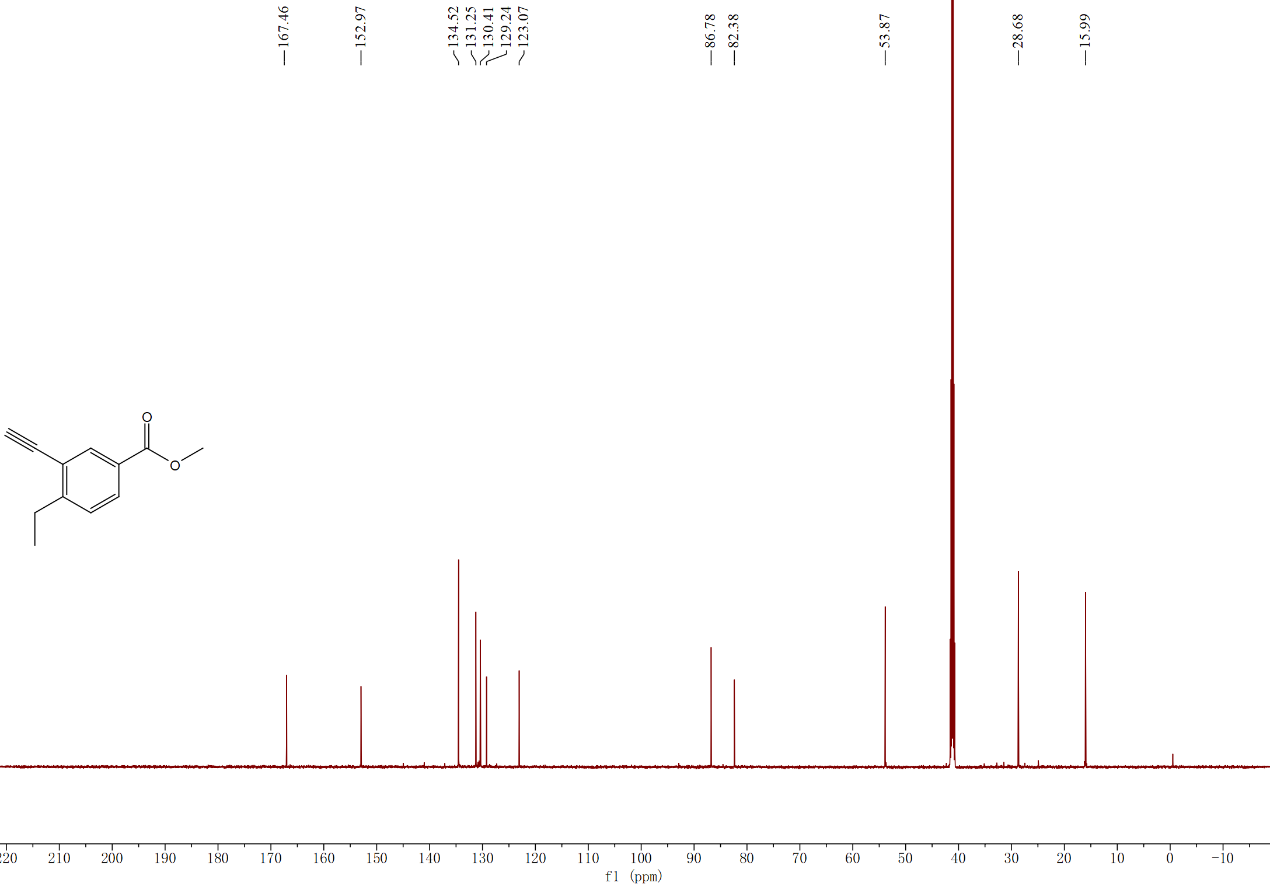
**

Compound **S13**

**
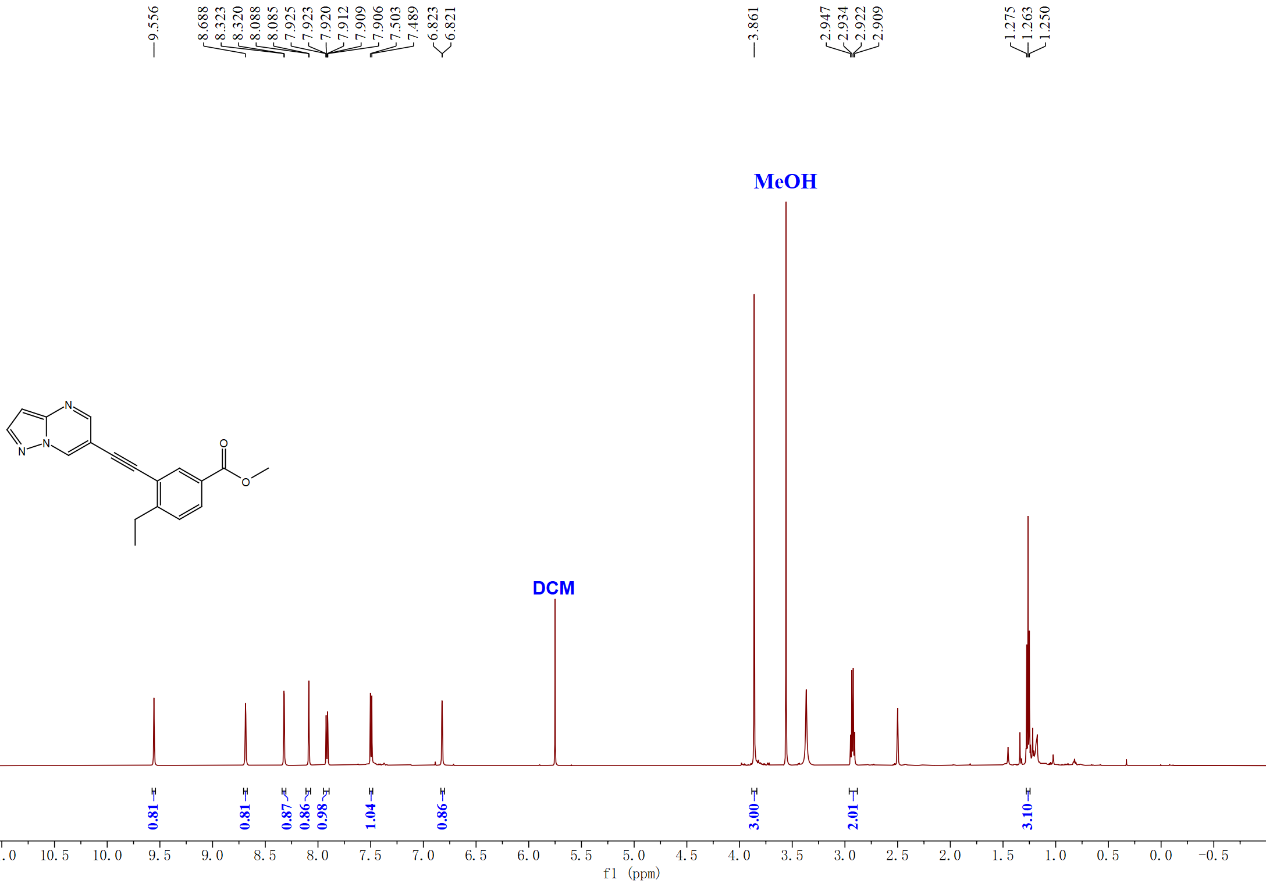
**

**
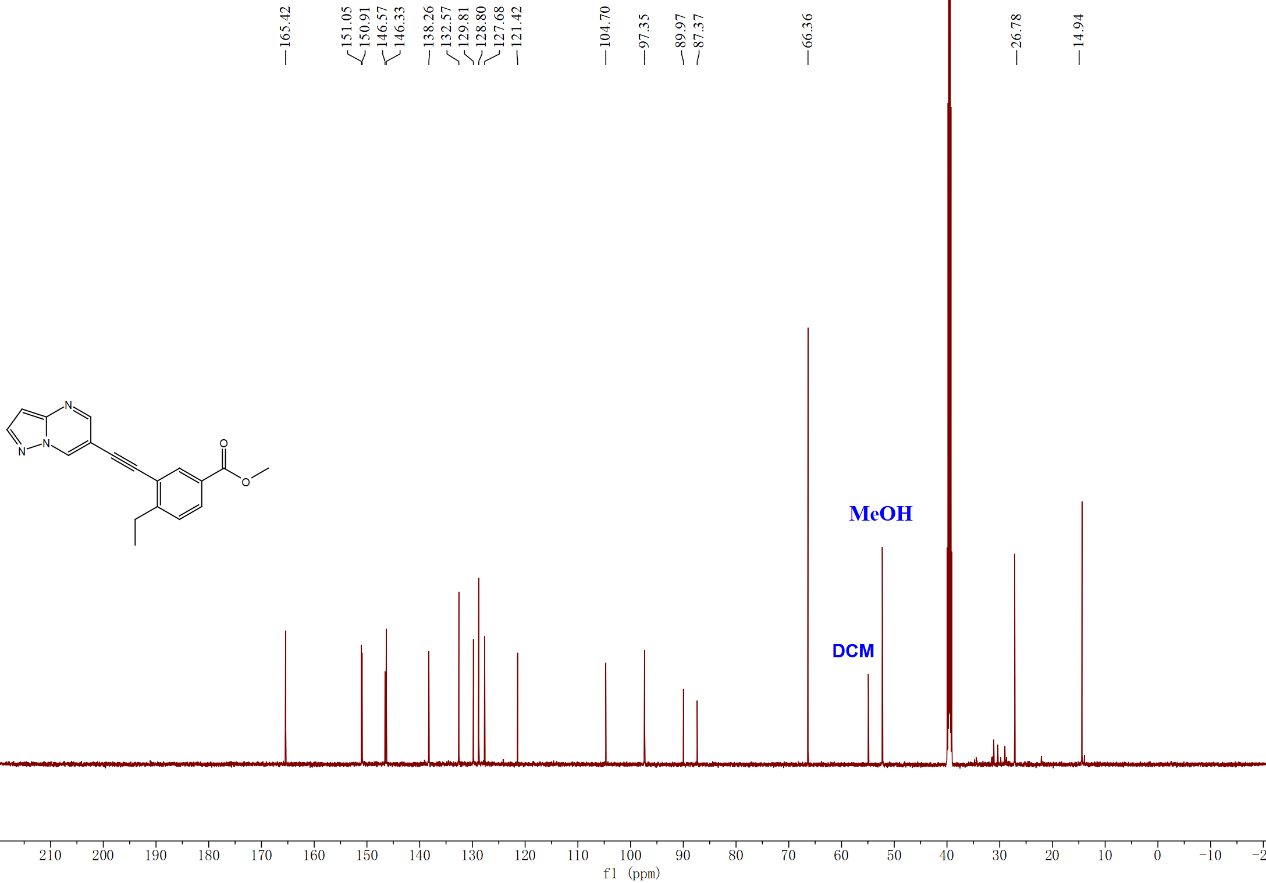
**

Compound **S14**

**
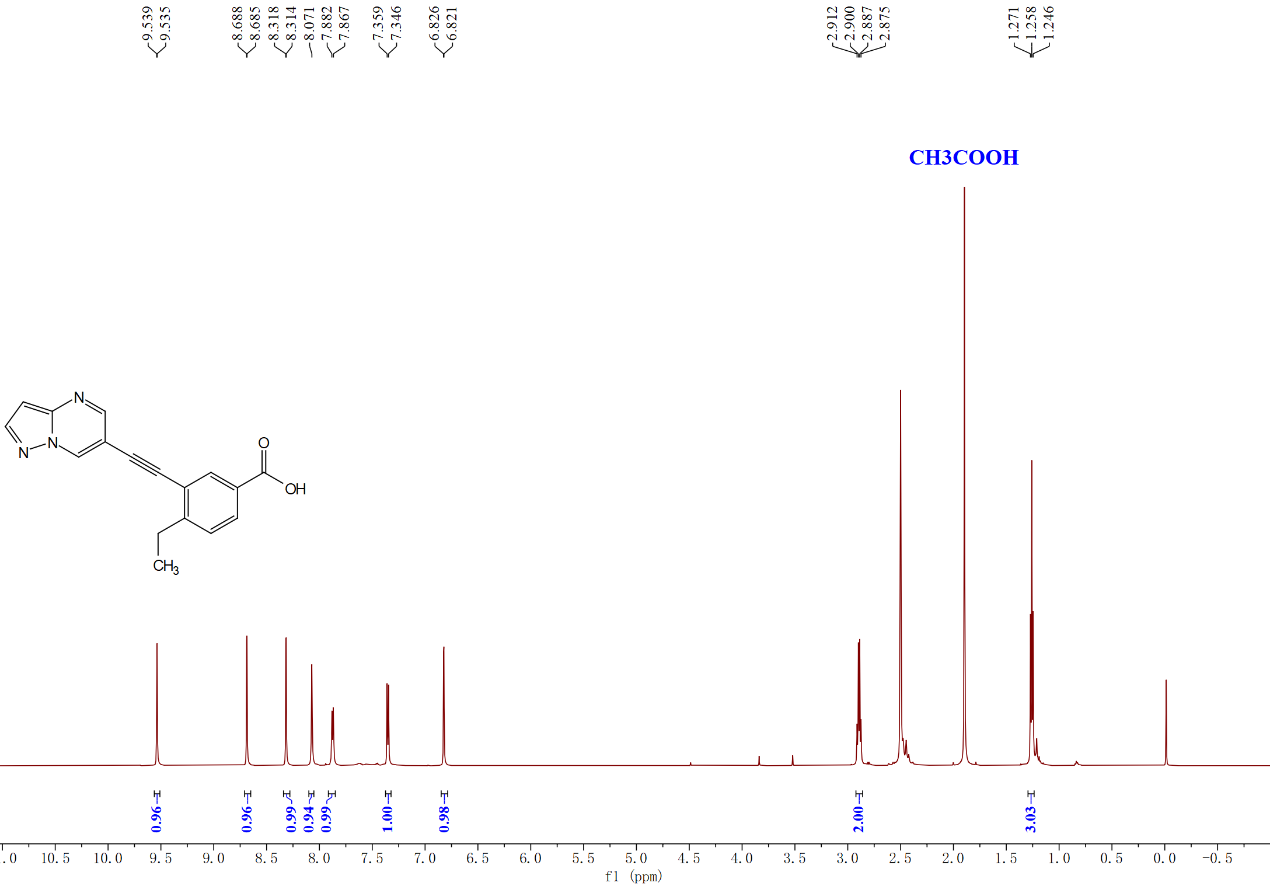
**

**
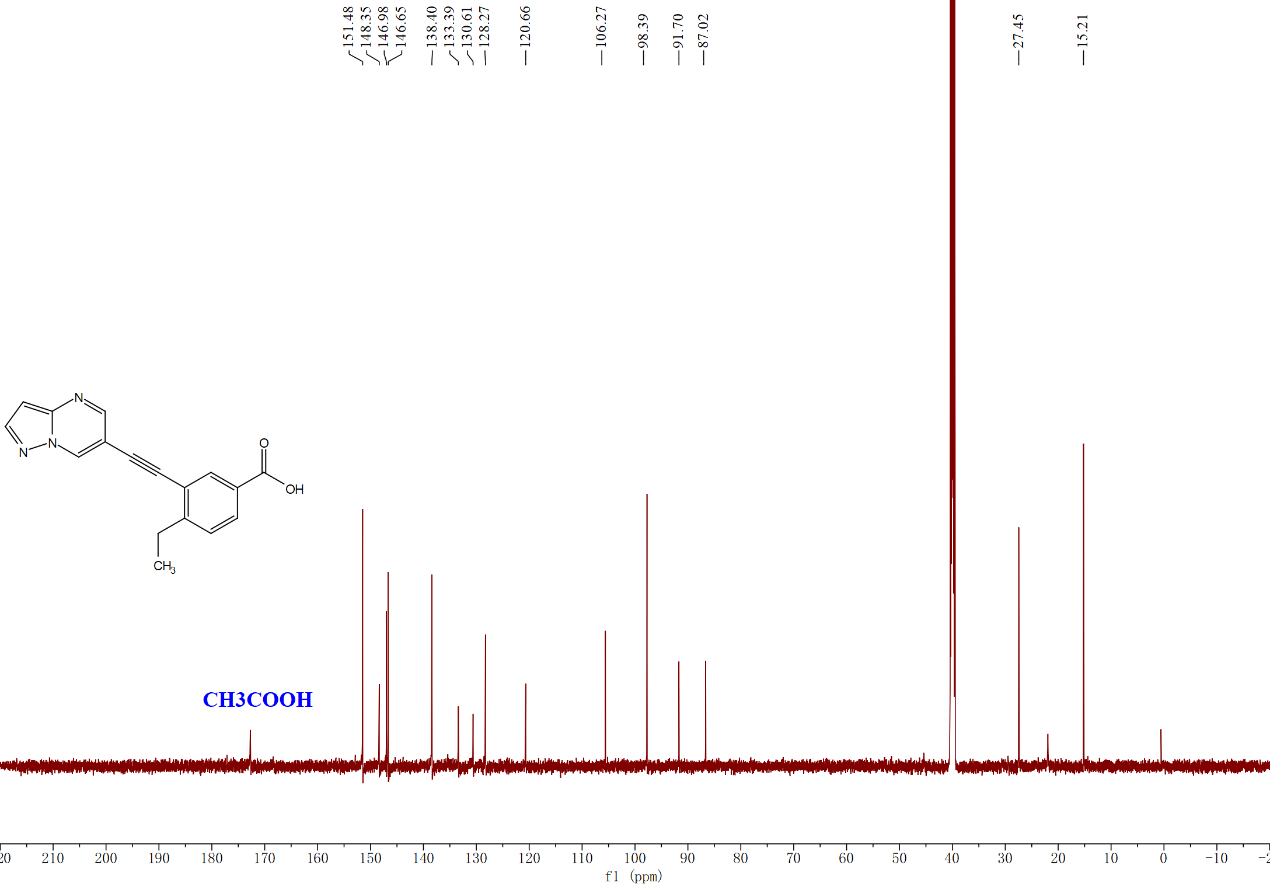
**
